# Vitamin B_12_ and hydrogen atom transfer cooperative catalysis as a hydride nucleophile mimic in epoxide ring opening

**DOI:** 10.1016/j.xcrp.2023.101372

**Published:** 2023-04-12

**Authors:** Brian E. Funk, Martin Pauze, Yen-Chu Lu, Austin J. Moser, Gemma Wolf, Julian G. West

**Affiliations:** 1 Department of Chemistry, Rice University, Houston, TX 77030, USA; 2 Lead contact

## Abstract

Epoxide ring-opening reactions have long been utilized to furnish alcohol products that are valuable in many subfields of chemistry. While many epoxide-opening reactions are known, the hydrogenative opening of epoxides via ionic means remains challenging because of harsh conditions and reactive hydride nucleophiles. Recent progress has shown that radical chemistry can achieve hydrogenative epoxide ring opening under relatively mild conditions; however, these methods invariably require oxophilic metal catalysts and sensitive reagents. In response to these challenges, we report a new approach to epoxide ring-opening hydrogenation using bio-inspired Earth-abundant vitamin B_12_ and thiol-centric hydrogen atom transfer (HAT) co-catalysis to produce Markovnikov alcohols under visible light irradiation. This powerful reaction system exhibits a broad substrate scope, including a number of electrophilic and reductively labile functionalities that would otherwise be susceptible to reduction or cleavage by hydride nucleophiles, and preliminary mechanistic experiments are consistent with a radical process.

## Graphical Abstract

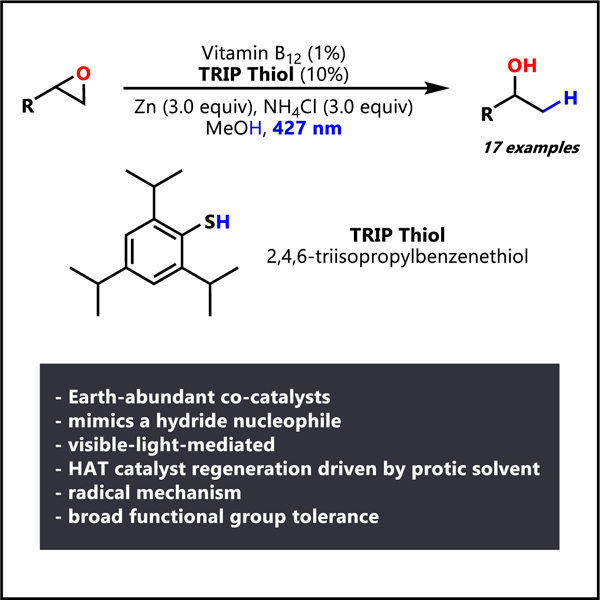

Here, Funk et al. develop a mild synthetic method for hydrogenative epoxide ring opening. The co-catalytic system uses Earth-abundant vitamin B_12_ and TRIP thiol to efficiently produce Markovnikov alcohols with good yield and broad functional group tolerance.

## INTRODUCTION

**Figure 1.**
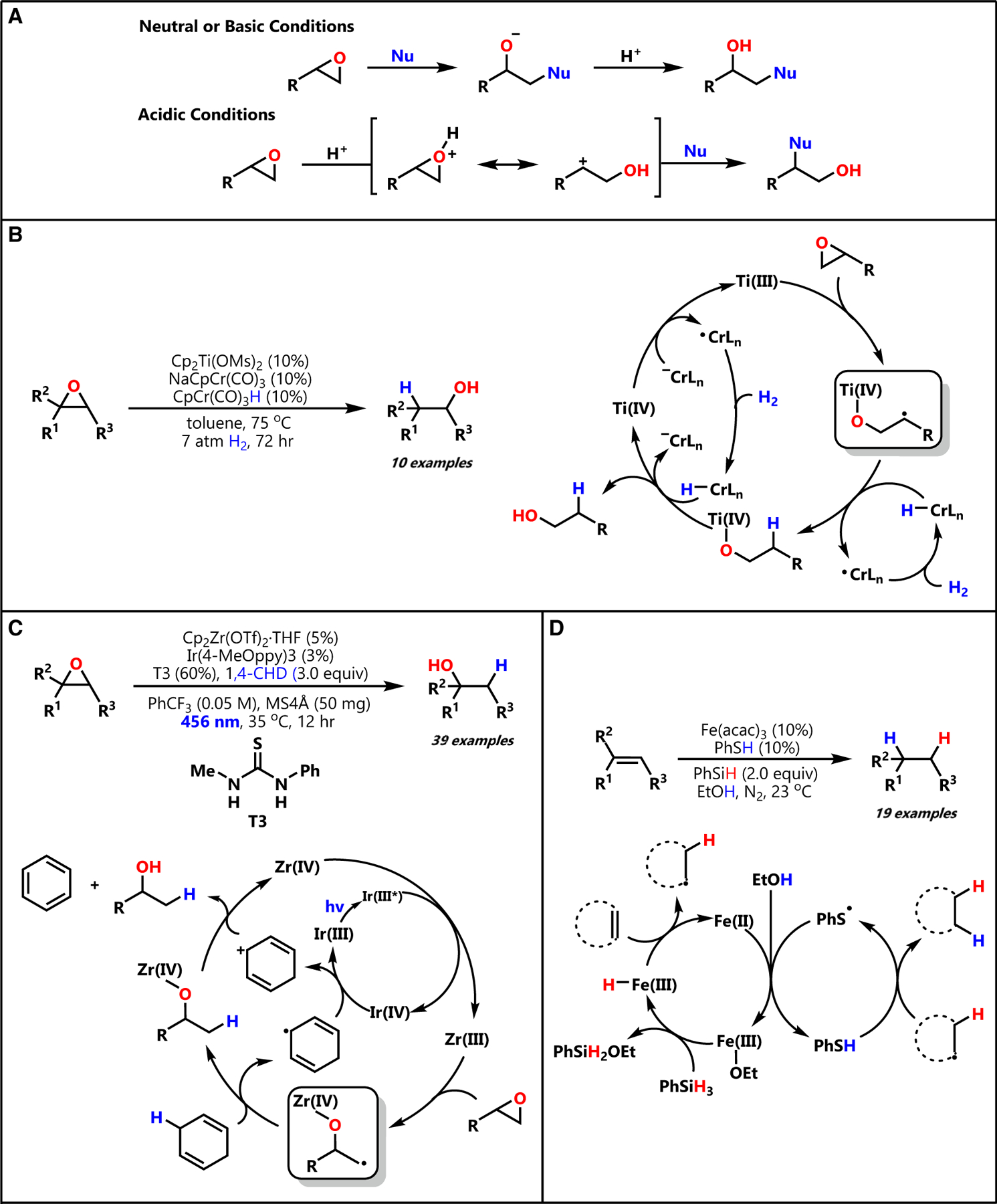
Literature precedent for metal-catalyzed ring-opening hydrogenation of epoxides (A) Condition-dependent regioselectivity of two-electron nucleophilic epoxide ring opening. (B) Gansäuer’s titanocene-catalyzed epoxide opening with Cr/H_2_ hydrogenation. (C)Yamaguchi’s zirconocene-catalyzed epoxide opening with 1,4-CHD hydrogen source. (D) Kattamuri’s thiol-centric hydrogen atom transfer catalysis applied in alkene hydrogenation.

The epoxide ring is a highly valuable structural motif in organic molecules due to its propensity to open upon attack by a nucleophile. The ring opening of epoxides has been well studied and is shown to be possible using a broad range of nucleophiles including alcohols, amines, thiols, halides, and hydrides.^1,2^ This level of nucleophile compatibility has established the epoxide ring as a gateway to a wide variety of functionalized alcohol products.^1–6^ The primary driving force for epoxide ring-opening reactions is thought to be the release of strain in the three-membered ring system,^3^ and the regioselectivity of nucleophilic attack on unsymmetrical epoxides is largely condition dependent (Figure 1A). In neutral or basic conditions, nucleophilic attack favors the less-substituted carbon of the ring, and this regioselectivity is believed to be sterically driven.^3–5^ In acidic conditions, preferential attack by nucleophiles at the more-substituted carbon is observed. As such behavior cannot be justified by steric interactions, it is proposed that acid activation of the epoxide ring promotes anti-Markovnikov ring opening by lowering the effective C–O bond strength at the more-substituted position, a phenomenon that can rationalized by carbocation stability.^3,5^ Although the regioselectivity of nucleophilic attack and the nucleophile compatibility of traditional epoxide ring-opening reactions is well established, challenges arise when applying them in synthetic pathways, especially using stronger nucleophiles like hydrides, for example, which exhibit a lack of chemoselectivity to the epoxide ring relative to other electrophilic functional groups and can be detrimental to reductively labile protecting groups. As such, there is a strong need to provide mild hydride nucleophile mimics to make epoxide ring opening more accessible in chemical synthesis.

The introduction of metal catalysis to epoxide ring opening has sparked significant progress in making the aforementioned hydride mimics a reality. Some early examples of epoxide ring-opening hydrogenation showed that rhodium catalysts could convert styrene oxide to 2-phenylethanol^7,8^; however, the substrate compatibility of these systems was limited to styrene oxides. More recently, as an effort was made to move away from noble-metal catalysts, Gansäuer and co-workers made significant strides investigating titanocene complexes as a means to catalyze epoxide ring opening.^9^ The unique ring-opening regioselectivity that they observed, opposite to that of traditional S_N_2, as well as the introduction of radical chemistry to these systems enabled by titanocene complexes marked an exciting advancement in metal-catalyzed epoxide ring opening.^9^ In 2019, Norton and Gansäuer reported a method to synthesize anti-Markovnikov alcohols from epoxides (Figure 1B), again employing a titanocene catalyst, and took advantage of the radical chemistry that arises by pairing this complex with chromium-mediated hydrogen atom transfer (HAT) in a co-catalytic system.^10^ The broad functional group tolerance demonstrated by their system demonstrates the versatility of HAT in realizing a hydride nucleophile mimic when paired with a co-catalyst able to achieve radical epoxide ring opening.^10^ While a beautiful contribution, this approach is only capable of producing anti-Markovnikov alcohols by virtue of the titanocene ring-opening regiochemistry and relies on exceptionally sensitive reduced titanium and chromium species as catalysts.

In 2022, Yamaguchi and co-workers presented a means of Markovnikov alcohol synthesis by epoxide ring opening and subsequent hydrogenation, this time catalyzed by zirconocene.^11^ There are many intriguing aspects of this work, among them demonstration of a Cp_2_Zr(OTf)_2_•THF complex as a regioselectivity complement to titanocenes in metal-catalyzed radical epoxide ring opening, with radical generation occurring at the less-substituted position of the epoxide ring (Figure 1C). Additionally, they demonstrated that a stoichiometric, organic hydrogen atom source, 1,4-cyclohexadiene (1,4-CHD), could be used to reduce both the carbon-centered radical resulting from epoxide opening and a terminal reductant for the zironocene catalyst. Finally, this reduction is driven by an iridium photoredox catalyst, showcasing the ability of visible light to drive epoxide-opening reactions. Despite being a significant advance in regioselective radical epoxide reduction, several challenges remain, including the need for a stoichiometric organic hydrogen atom donor, the use of a sensitive and oxophilic zirconocene complex, and reliance on a rare and unsustainable iridium photocatalyst to drive this reactivity.

Taking these precedents together, we wondered if we might overcome these challenges in radical epoxide reduction through strategic application of both thiol and vitamin B_12_ catalysis.

First, we wondered if the sensitive chromium HAT catalyst of Norton and Gansäuer or the stoichiometric 1,4-CHD HAT donor of Yamaguchi might be replaced by a simple thiol catalyst. Building on early work by Roberts^12,13^ on the use of thiols as HAT catalysts for radical chain reactions, our lab has recently demonstrated that thiols can be used as HAT co-catalysts in the presence of iron catalysts to achieve either alkene hydrogenation or decarboxylative protonation.^14,15^ We found the use of thiol co-catalysts to be key in these reactions, as they allow for the hydrogen atom donor to be continuously regenerated simply from a reducing co-catalyst and protic solvent (the proposed catalytic cycle for our alkene hydrogenation system is shown in Figure 1D). Further, thiols are relatively resistant to spontaneous oxidation, allowing for our previously developed reactions to be performed readily on the benchtop. Thus, we imagined that a thiol co-catalyst would be an ideal HAT donor to reduce the carbon-centered radical product of radical epoxide opening. However, the successful use of a catalytic thiol depends on finding a robust epoxide-opening co-catalyst able to function in polar, protic solvents.

**Figure 2.**
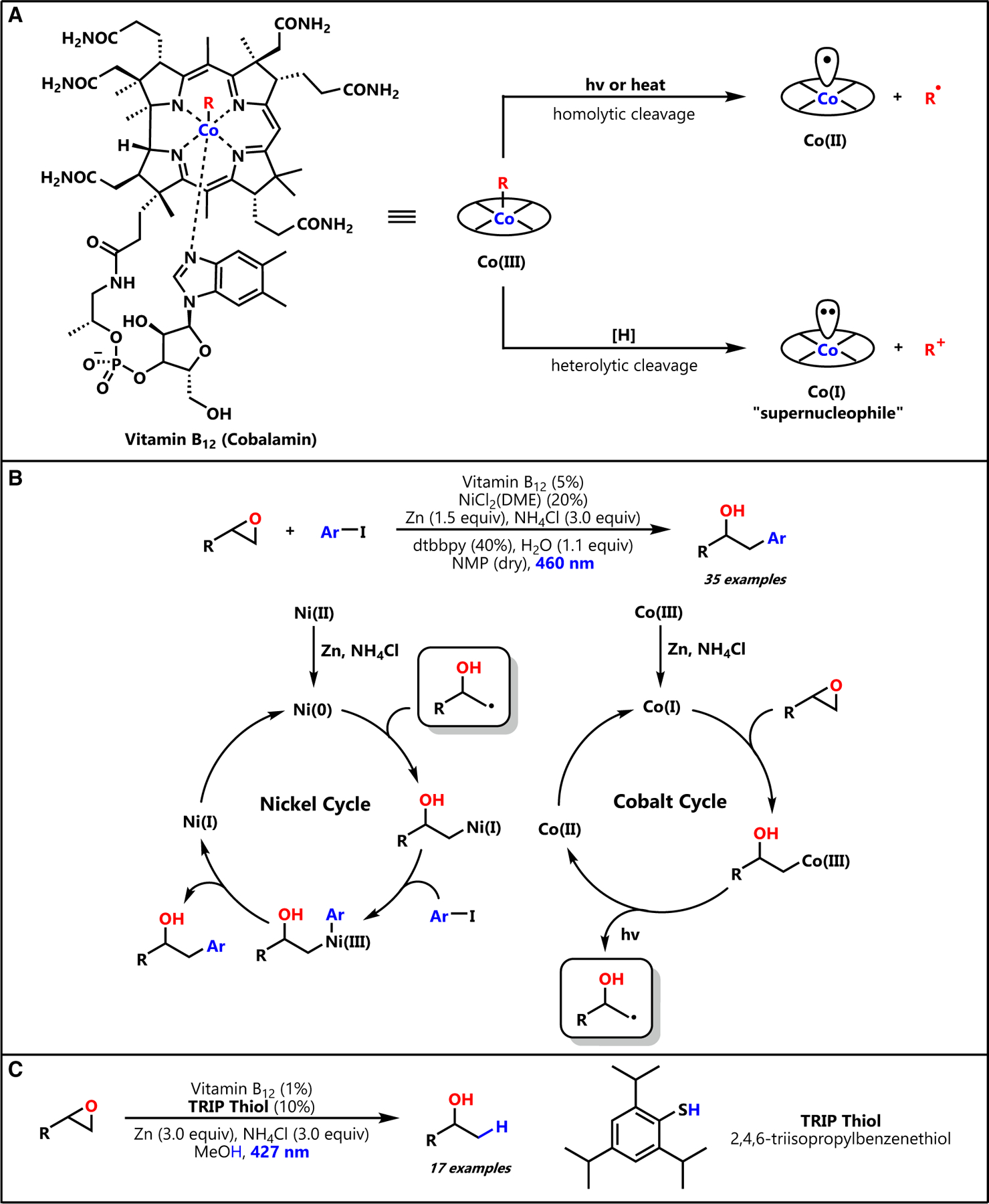
Vitamin B_12_ photoreactivity and its application in epoxide ring-opening reactions (A) Vitamin B_12_ structure and general reactivity. (B) Gryko’s vitamin B_12_-catalyzed epoxide ring opening as a precursor to nickel cross coupling. (C) This work: vitamin B_12_ and thiol HAT co-catalyzed epoxide ring-opening hydrogenation.

Toward generating this ring-opened radical intermediate, our attention immediately turned to vitamin B_12_ (VB_12_) photocatalysis, an exciting area of chemical catalysis that we have recently leveraged to achieve a mild olefination reaction.^16^ We were drawn to VB_12_ as a photocatalyst as it is a naturally occurring, widely available cobalt complex with a wide range of ionic and radical reactivity (Figure 2A) that has been used by nature and synthetic chemists alike to achieve many powerful transformations.^17–20^

Our work was inspired by the biological mechanism of photodetection in the CarH enzyme of *T. thermophilus* wherein light is detected through homolysis of a Co–C bond of the AdoCbl vitamer of VB_12_, transiently generating a carbon-centered radical that is desaturated through HAT to the Co(II) intermediate.^21^ This reactivity permits the mild conversion of organometallic Co(III) species to olefins under visible light irradiation. Concurrently, we were cognizant of the ‘‘supernucleophilicity’’ of the Co(I) form of VB_12_, enabling this intermediate to readily alkylate with a wide variety of electrophiles to generate the requisite Co(III) organometallic adducts.^17,22^ Intriguingly, the Co(I) supernucleophile has also been shown to readily open epoxides at the less-substituted position, starting with Scheffold’s enantioselective isomerization of meso epoxides,^23^ aziridines,^24^ and cyclopropanes^25^ to ring-opened alkenes, presumably through a similar homolysis/HAT cascade of the organometallic Co(III) species. Excitingly, Gryko and co-workers recently reported a reaction that extends this strategy to arylate epoxides using VB_12_ photolysis and nickel co-catalysis (Figure 2B).^26^ Importantly, this study modulates the reaction conditions to permit cage escape of the carbon-centered radical formed after Co–C bond photolysis, allowing for this intermediate to be intercepted by the nickel catalyst and cross coupled with a suitable aryl electrophile. Not only is this epoxide opening/photolysis/cage escape cascade exactly the reactivity we imagined for an epoxide-opening co-catalyst, but the demonstrated compatibility of VB_12_ with polar, protic solvents such as methanol suggested that it might enable epoxide reduction under significantly milder conditions.

In combining these two reactivity manifolds, we enable photocatalytic reduction of epoxides using cooperative VB_12_ and thiol co-catalysis to function as a mild hydride nucleophile mimic. (Figure 2C). This method permits the direct synthesis of Markovnikov alcohols from epoxides in hitherto-inaccessible polar, protic solvents using only Earth-abundant element catalysts and simple inorganic reagents. Herein, we report the first demonstration of cooperative VB_12_ and thiol co-catalysis to achieve mild epoxide reduction with high efficiency and broad scope.

## RESULTS AND DISCUSSION

### Reaction development and optimization

We first set out to investigate the potential of a hydrogenative epoxide ring-opening reaction co-catalyzed by VB_12_ and HAT. To our delight, catalytic amounts of VB_12_ (CNCbl, 1%) and 2,4,6-triisopropylbenzenethiol (TRIP Thiol, 10%), and a stoichiometric reducing system of Zn (3 equiv) and NH_4_Cl (3 equiv) enabled the ring-opening hydrogenation of (2,3-epoxypropyl)benzene (**1A**) when irradiated with blue light (427 nm) and produced the desired Markovnikov alcohol product (**2A**) in 84% yield (Table 1, entry 1). The presence of photocatalytic VB_12_ and irradiation by light were found to be critical to reaction progress (Table 1, entries 2–3). Further, VB_12_ appears uniquely equipped to catalyze this reaction, as our efforts with synthetic Co(II) (salen) as the photocatalyst resulted in diminished, though non-zero, yields (Table 1, entries 4–5). We found the HAT co-catalyst to be similarly vital to product formation (Table 1, entry 6). Interestingly, presumably due to reducing conditions, we observed that disulfides are a viable precursor to thiol HAT donors. Further investigation revealed bulkier HAT catalysts are more efficient in producing the desired alcohol product. In fact, diphenyl disulfide was discovered to act as a competitive nucleophile in the epoxide ring opening to form a thioether side product, leading to reduced Markovnikov alcohol yield (52%) relative to bis(2,4,6-triisopropylphenyl) disulfide (TRIP disulfide, 87%) (Table 1, entries 7 and 9). Though TRIP disulfide proved to be an effective HAT co-catalyst, we opted to use TRIP thiol directly, as it proceeds with almost identical yield. Excitingly, a minimal amount of VB_12_ (1%) was sufficient to catalyze the epoxide ring opening. Though ethanol was explored as a solvent, due to the limited solubility of VB_12_, we selected methanol as our reaction solvent.

### VB_12_ and thiol HAT co-catalyzed epoxide ring opening

**Figure 3.**
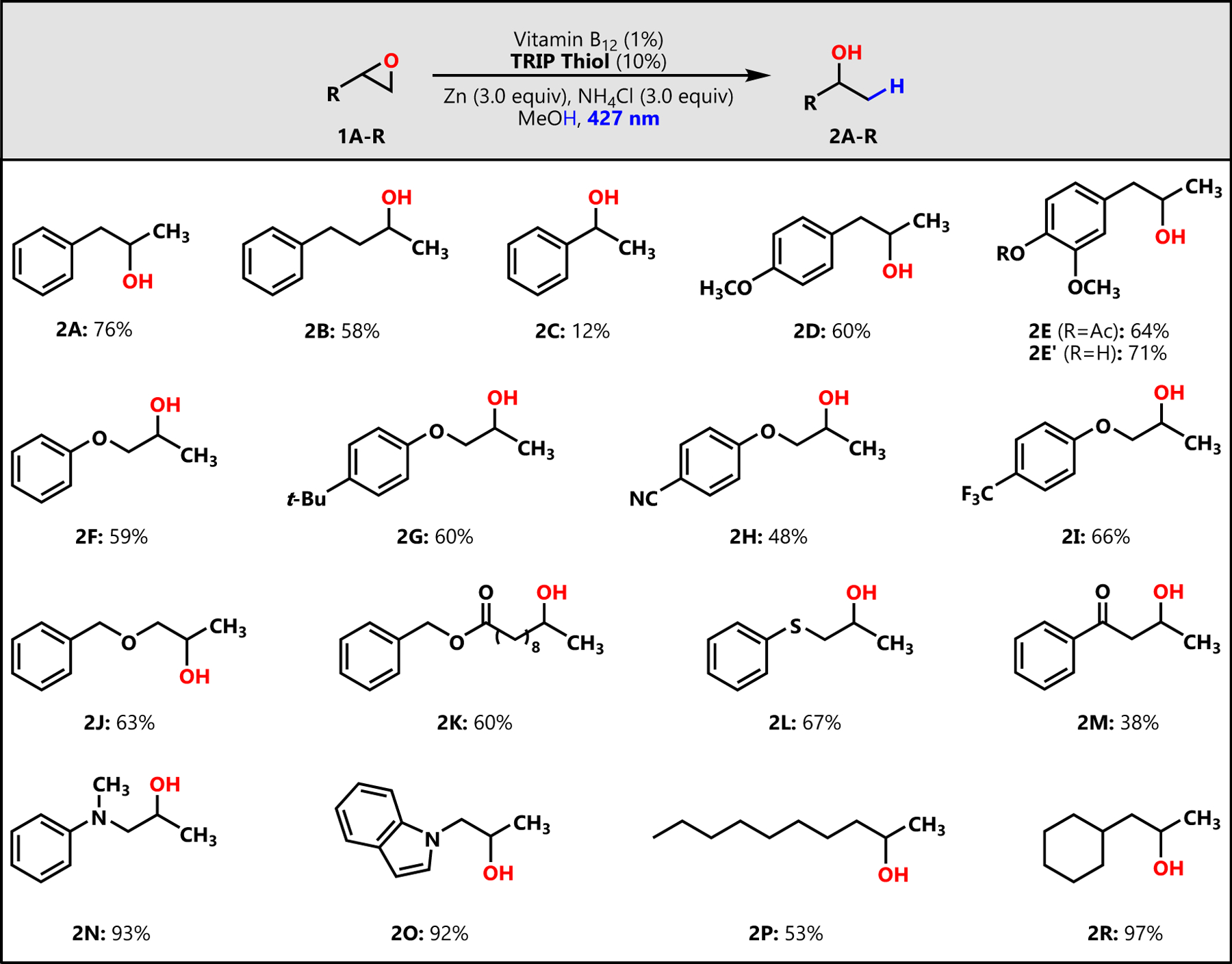
Substrate scope of vitamin B_12_ and HAT co-catalyzed epoxide ring opening Reaction conditions: epoxide (0.2–0.4 mmol, 1 equiv), vitamin B_12_ (cyanocobalamin, 1 mol %), TRIP thiol (2,4,6-triisopropylbenzenethiol, 10 mol %), Zn (3 equiv), NH_4_Cl (3 equiv), MeOH (0.1 mmol epoxide/mL), 427 nm LED (Kessil), 2–48 h, isolated yield. Products **2E** (2 h) and **2E**′ (16 h) both form by reaction of epoxide **1E**.

With optimized conditions in hand, we looked to explore the substrate scope and functional group tolerance of our hydride nucleophile mimic (Figure 3). We found that a range of terminal epoxides react under these conditions and afford Markovnikov alcohol products in moderate to excellent yields (up to 97%). To our delight, we observed that in addition to aliphatic and hydrocarbon epoxides, our system tolerates diverse functional groups including ethers (**1F** and **1G**), tertiary amines (**1N**), heterocycles (**1O**), fluorine-containing moieties (**1I**), and sulfides (**1L**). Further, epoxides containing electrophilic groups like nitriles (**1H**) and ketones (**1M**) that would otherwise be susceptible to attack by hydride nucleophiles were not reduced in our reaction and produced alcohols **2H** and **2M** in moderate yields (48% and 38%, respectively). Substrates with reductively labile protecting groups like benzyl ethers and benzyl esters (**1J-K**) were tolerated and performed well in forming alcohols **2J** (63% yield) and **2K** (60% yield).

Interestingly, our system is conditionally tolerant of acetyl groups. When reacted in our system, epoxide **1E** retained its *p*-acetyl motif after total conversion of starting material at 2 h and formed **2E** in 64% yield. However, when **1E** was allowed to react for 16 h, the acetyl group was cleaved, leading to the *p*-hydroxyl group in alcohol **2E′**, which we isolated in 71% yield. The reaction of styrene oxide (**1C**) was particularly inefficient, producing the desired 1-phenylethanol (**2C**) in only 12% yield. Upon further investigation of the reaction profile for **1C**, it was observed that styrene and ethyl benzene were the major products, with the reaction of **1C** affording approximately 30% (nuclear magnetic resonance [NMR] yield) of each. We also assessed the reaction profiles of **1A**, **1E**, **1K**, and **1O**. The results revealed that in addition to hydrogenative epoxide ring opening, our system enables a minor deoxygenation side pathway that forms terminal olefins from the epoxide substrates. With the exception of **1C**, however, our system heavily favors the presented ring opening such that the typical ratio of alcohol to olefin is 4:1 or higher. Finally, we wondered whether our reaction system would be effective for cyclic, internal, or tri-substituted epoxides. As such, cyclooctene oxide (**S8**) and (3-(2-(3,3-dimethyloxiran-2-yl)ethyl)-3-methyloxiran-2-yl)methyl benzoate (**S9**) were reacted under our optimized conditions. In either case, we observed no conversion of starting material after 16 h, which suggests that the scope of this system is limited to terminal epoxides, likely due to VB_12_’s notably large molecular framework.

### Mechanistic considerations

**Figure 4.**
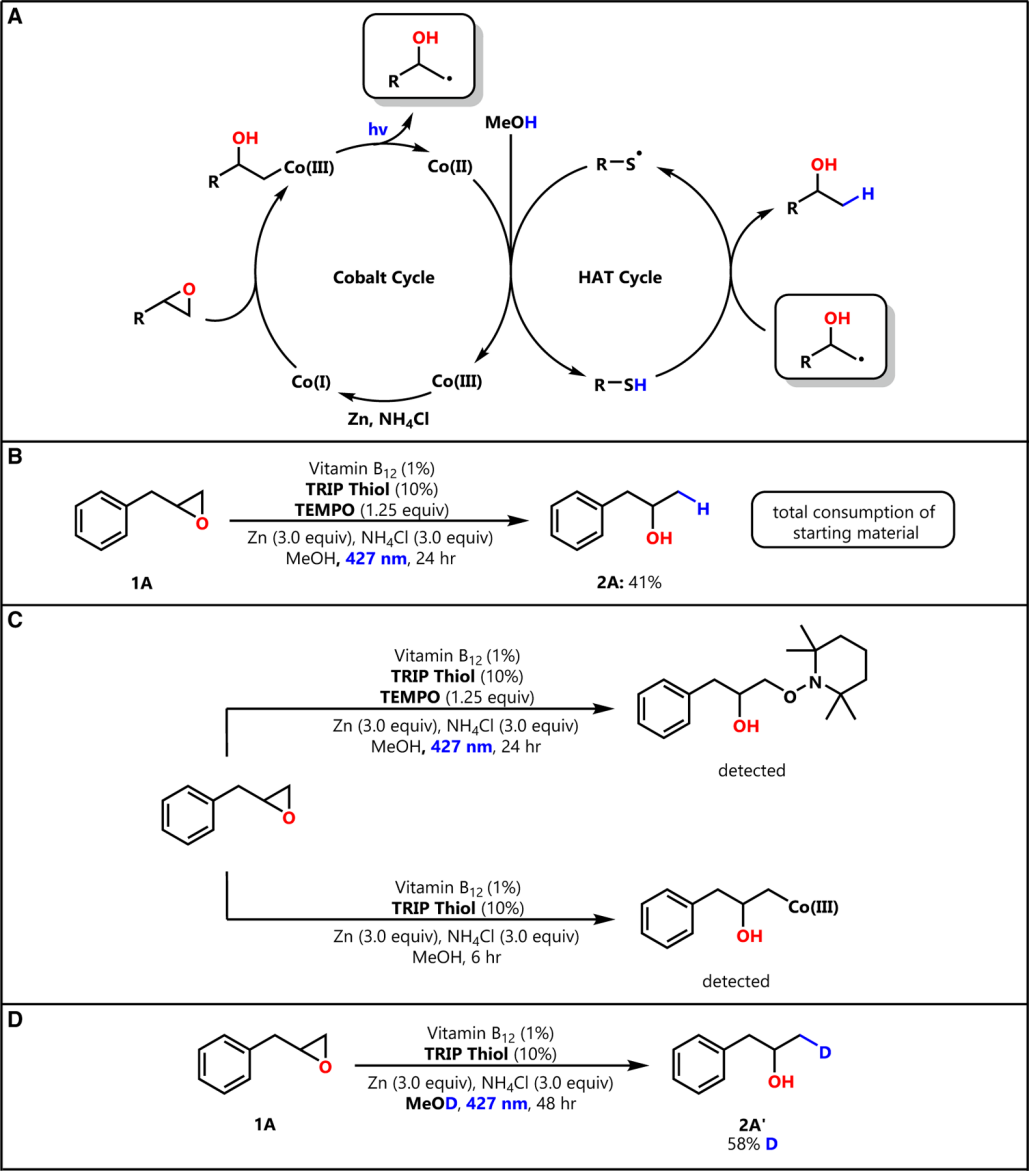
Mechanistic investigations (A) Proposed mechanism; see supplemental experimental procedures (pages S6–S13) for more details. (B) TEMPO radical trap inhibits product formation. (C) Key mechanistic intermediates detected. (D) Deuterated methanol solvent leads to deuterium incorporation at hydrogenation site.

Encouraged by the functional group tolerance and effectiveness of our hydrogenative epoxide ring opening, we sought to gain more insight into the underlying mechanism (Figure 4A). First, we hoped to gauge the likelihood of a radical mechanism, a critical aspect of our mechanistic design. Further, we wished to gain insight into the role that methanol plays, if any, in the proposed thiol HAT cycle.

Hoping to gain support of the radical nature of the mechanism, we conducted a radical trap experiment with TEMPO. We found that the addition of stoichiometric (1.25 equiv) TEMPO led to significantly diminished product yield (41%) relative to the control (84%) (Figure 4B). This result is consistent with a radical mechanism impacted by TEMPO inhibition. Additionally, we were able to detect the resulting TEMPO adduct from the crude reaction mixture by liquid chromatography with mass spectrometry (LCMS) analysis (Figure 4C). Although this result is strongly supportive of a radical mechanism, we are cognizant that TEMPO radical trap results must be interpreted with caution.^27^

Emboldened by this TEMPO result, we sought to detect the key radical intermediate of the reaction with a radical clock experiment (see supplemental experimental procedures). Using the radical clock substrate 1,2-epoxy-5-hexene, we sought to observe 3-methylcyclopentanol, expected to be the 5-*exo*-trig cyclization product forming as a result of radical generation at the terminal site of the epoxide ring. Gas chromatography (GC)-MS analysis of the crude product produced a contributing product signal with a significant match to the known mass spectrum of 3-methylcyclopentanol (Figures S2–S4). Although seemingly supportive of our proposed mechanism, our inability to purify 3-methylcyclopentanol and visualize it using NMR prompted further experimentation. We reacted **1A** according to our optimized conditions but did not irradiate the system with light. In this manner, we sought to detect the cobalt adduct (Figure 4C) that forms upon nucleophilic attack by Co(I) on the epoxide. This intermediate is short lived under our optimized conditions, as the Co–C bond undergoes photolysis; however, in the absence of light, we expected this adduct to remain intact. To our delight, we were able to observe this intermediate by LCMS analysis of the crude reaction mixture.

Finally, we investigated the extent to which methanol was involved in facilitating catalytic HAT by TRIP Thiol through solvent isotope studies. In accordance with our earlier findings in thiol-catalyzed HAT, we expected methanol to be active in exchanging its protic hydrogen with our thiol co-catalysts.^13,14^ Consistent with our previous studies, the reaction of epoxide **2A** in deuterated methanol resulted in high (58%) levels of deuterium incorporation at the site of the new C–H bond (Figure 4D). The lack of deuteration observed at any other site in **2A′** is similarly consistent with HAT occurring via the thiol catalyst. The moderate extent of deuterium incorporation is consistent with the competitive proton donors (e.g., NH_4_Cl) in our system. Together, the results of the TEMPO trap, intermediate detection, radical clock, and deuterium-labeling experiments are consistent with our proposed mechanism.

We envisioned the mechanism of this dually catalytic system beginning with the Co(III) state of VB_12_ (Figure 4A). In the presence of Zn/NH_4_Cl, we expected that this species may be reduced to Co(I), a supernucleophile known to readily react with electrophiles to produce Co(III) alkyl intermediates.^22^ We envisioned that nucleophilic attack on an epoxide by Co(I) would open the three-membered ring at the Markovnikov position through Co(III)–C bond formation, consistent with the findings of Gryko and co-workers.^26^ Moving forward, we imagined that this Co(III) intermediate may be photolyzed by visible light irradiation to produce a Co(II) metalloradical as well as a carbon-centered radical at the Markovnikov site of the epoxide. We envisioned that this alkyl radical intermediate would be readily reduced by our thiol co-catalyst, a species known to undergo efficient HAT with carbon-centered radicals due to its electron-rich nature and low S–H bond strength,^12,28,29^ to produce a Markovnikov alcohol and leave behind a thiyl radical. Building off our previous work with alkene hydrogenation^14^ wherein the thiol HAT catalyst was regenerated by protic solvent in the presence of a reduced iron (Fe(II)) species, we imagine that this thiyl radical may be converted back to a thiol by our protic methanol solvent in the presence of reduced cobalt (Co(II)) leftover from previous photolysis to complete both catalytic cycles. Together, these pathways demonstrate a means to produce Markovnikov alcohols using VB_12_ and HAT co-catalysis under mild conditions that mimic hydride nucleophiles.

In summary, we have developed a hydrogenative epoxide ring-opening reaction co-catalyzed by VB_12_ and thiol HAT. This system presents a new method for producing Markovnikov alcohols from terminal epoxides in a mild manner that mimics hydride nucleophiles and displays tolerance of electrophilic and reductively labile functional groups and proceeds with Earth-abundant element catalysts in wet, protic solvents. Our preliminary mechanistic studies support our proposed mechanism, highlighted by VB_12_-photocatalyzed epoxide ring opening and radical generation followed by catalytic thiol HAT. The development of this reaction marks the first demonstration of synergistic VB_12_ photocatalysis and thiol HAT catalysis, and our laboratory is actively investigating new applications of this powerful system.

## EXPERIMENTAL PROCEDURES

### Resource availability

#### Lead contact

Further information and requests for resources should be directed to and will be fulfilled by the lead contact, Julian G. West (jgwest@rice.edu).

#### Materials availability

This study did not require the design nor use of reagents or catalysts unique to our laboratory. All chemicals were purchased from commercial suppliers and used without further purification. For more information on materials used, chemical vendors, and general methods followed, please reference the supplemental experimental procedures in Document S1.

#### Data and code availability

The primary data supporting the findings of this investigation are available in Document S1. Additionally, mass spectrometry data of newly characterized substrates and products are available from the lead contact upon reasonable request.

### General procedure for VB_12_-HAT co-catalyzed epoxide ring opening

To an 8 mL septum screw-capped vial equipped with magnetic stir bar were added epoxide (1 equiv), VB_12_ (cyanocobalamin, 1 mol %), Zn (3 equiv), and NH_4_Cl (3 equiv). TRIP thiol was then added from a 0.1 mmol/mL stock solution in methanol (10 mol %) followed by the methanol solvent (0.1 mmol epoxide/mL methanol). This solution was sparged with a nitrogen balloon for 10 min, and the vial was twice sealed with parafilm. The mixture was then stirred and irradiated with a 427 nm LED (Kessil). After reaction completion, the reaction mixture was concentrated *in vacuo*, resuspended in dichloromethane, and sonicated before being passed through a cotton pipette filter. The filtrate was concentrated *in vacuo* and purified by silica gel column chromatography or preparative TLC to isolate the alcohol products.

## Supplementary Material

1

## Figures and Tables

**Table 1.** Reaction optimization

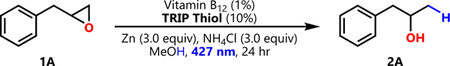		
Entry	Deviation from optimized conditions	Yield (%)

1	none	84
2	no light	trace
3	no Vitamin B_12_	trace
4	cobalt(II) (salen) (1%) as photocatalyst	48
5	cobalt(II) (salen) (10%) as photocatalyst	66
6	no HAT donor	trace
7	diphenyl disulfide (5%) as HAT donor	52
8	TRIP disulfide (1%) as HAT donor	73
9	TRIP disulfide (5%) as HAT donor	87
10	TRIP disulfide (10%) as HAT donor	65
11	5% vitamin B_12_ & TRIP disulfide (5%) as HAT donor	68
12	5% vitamin B_12_ & TRIP disulfide (10%) as HAT donor	77

Reaction conditions: **1A** (0.2 mmol), vitamin B_12_ (cyanocobalamin, 1 mol %), TRIP thiol (2,4,6-triisopropylbenzenethiol, 10 mol %), Zn (0.6 mmol), NH_4_Cl (0.6 mmol), MeOH (2 mL), 427 nm LED (Kessil), 24 h, yield measured by ^1^H NMR using 1,3,5-trimethoxybenzene as an internal standard.

## Highlights

VB_12_ and thiol HAT co-catalysis enables hydrogenative epoxide ring opening

VB_12_ and TRIP thiol combine for an Earth-abundant co-catalytic system

Exceptionally mild conditions indicated by broad substrate scope

Mechanistic inquiries suggest a radical process with MeOH-driven thiol regeneration
