# Supplementary material for: Vitamin B12 and hydrogen atom transfer cooperative catalysis as a hydride nucleophile mimic in epoxide ring opening
Source: Cell Rep Phys Sci. Author manuscript; Available in PMC 2023 May 25. (PMC10210593; doi:10.1016/j.xcrp.2023.101372)
Supplement: 1 [file NIHMS1894097-supplement-1.pdf]

Cell Reports Physical Science, Volume *4*

Supplemental information

Vitamin B_{12} and hydrogen atom transfer cooperative catalysis as a hydride nucleophile mimic in epoxide ring opening

Brian E. Funk, Martin Pauze, Yen-Chu Lu, Austin J. Moser, Gemma Wolf, and Julian G. West

Supplemental Experimental Procedures

Table of Contents

1. General Information S3
2. Reaction Set-Up S4
3. Reaction Optimization S5
4. Mechanistic Considerations S6
5. Mass Balance Studies for Select Scope Compounds S14
5. Syntheses of Epoxide and Oxetane Substrates S21
6. Vitamin B_{12} Catalyzed Epoxide Ring Opening S27
9. ^{1}H NMR, ^{13}C NMR, and ^{19}F NMR Spectra S33
10. Supplemental References S67

General Information

Unless otherwise specified, all chemicals were purchased from Sigma Aldrich and used without any further purification. Styrene oxide, benzyl bromide, Dess Martin periodinane (DMP), and bis(2,4,6-triisopropylphenyl) disulfide (TRIP disulfide) were purchased from Oakwood Chemical. Benzaldehyde, potassium carbonate, ammonium chloride, and potassium hydroxide were purchased from Thermo Scientific. N-methyl aniline and diphenyl disulfide were purchased from Alfa Aesar. 2,4,6-triisopropylbenzenethiol was purchased from Ambeed. Epichlorohydrin was purchased from TCI. Zinc dust was purchased from Strem Chemicals. Allyl bromide was purchased from VWR. Anhydrous solvents and inert environments are used only when explicitly stated.

Silica gel column chromatography was carried out using Silicycle P60 silica gel (40-63 µm, 230-400 mesh). Additionally, column chromatography was conducted using a CombiFlash NextGen 300 Auto-Column equipped with RediSepRf Silver 24-gram Flash Column; for CombiFlash purification, samples are dry loaded in P60 silica gel (40-63 µm, 230-400 mesh) purchased from Silicycle®. Analytical thin-layer chromatography (TLC) was performed with Silicycle F-254 glass plates (250 µm). Analytical TLC was analyzed using short-wave UV light (254 nm) as a visualizing agent as well as $KMnO_4$ and Phosphomolybdic acid (PMA) as heat-developing stains prepared in our laboratory. Preparative TLC was conducted using Uniplate UV254 glass plates (1000 µm).

GC-MS chromatograms were collected on an Agilent 8860 GC and Agilent 5977B GC/MSD equipped with an Agilent J&W HP-5ms Ultra Inert (5%-phenyl)-methylpolysiloxane column (30 m x 0.25 mm x 0.25 µm film thickness). ALS injector used an injection volume of 1 µL and a temperature of 300 ^{0}C. Flow rate of H_2 carrier gas was maintained at a constant pressure of 1 psi. Oven temperature began and was held at 40 ^{0}C for two minutes, increased to 200 ^{0}C at a rate of 5 ^{0}C/min, then increased to 350 ^{0}C at a rate of 50 ^{0}C/min for a total run time of 37 minutes. The mass spectrometer was operated under standard conditions, and the instrument was tuned (via Agilent EI auto-tune routine) with a source temperature of 230 ^{0}C and a quadrupole temperature of 150 ^{0}C. In scan mode, the mass range was *m/z* = 25-550. Data analysis was performed using OpenLab CDS and MassHunter software.

^{1}H, ^{13}C, and ^{19}F NMR spectra were acquired on a Bruker 600 Avance Spectrometer operating at 600 MHz for ^{1}H NMR, 151 MHz for ^{13}C NMR, and 594 MHz for ^{19}F NMR. The spectra were calibrated based on residual non-deuterated solvent peaks ($CDCl_3$, δ 7.26 ppm in ^{1}H NMR and δ 77.0 ppm in ^{13}C NMR). Peak multiplicities are abbreviated as follows: s = singlet, d = doublet, t = triplet, q = quartet, m = multiplet, bs = broad singlet, dd = doublet of doublets, dt = doublet of triplets, dq = doublet of quartets, ddt = doublet of doublet of triplets, dqd = doublet of quartet of doublets.

Reaction Set-Up

A 427-nm, blue LED light (Kessil®, PR160L) was used at 100% intensity to irradiate the reactions. The reaction vial was positioned approximately 3 cm from the surface of the lamp such that the solvent-containing subsection was centered in the light beam. Though not explicitly shown, a three-sided "igloo" (tin foil taped to the inside of a cardboard 'C') was placed surrounding the stir plate-LED apparatus to contain the light. For all reactions (0.2 mmol and 0.4 mmol scale), 8-mL vials with screw-top septa-caps (Chemglass® CG-4909, 17x60 mm clear borosilicate glass w/ TFE septa) were used. No cooling fans nor light filters were employed for this reaction apparatus (**Figure S1**).

The spectral irradiance of the Kessil PRL160L-427 is available at the following address: https://kessil.com/products/science_PR160L.php

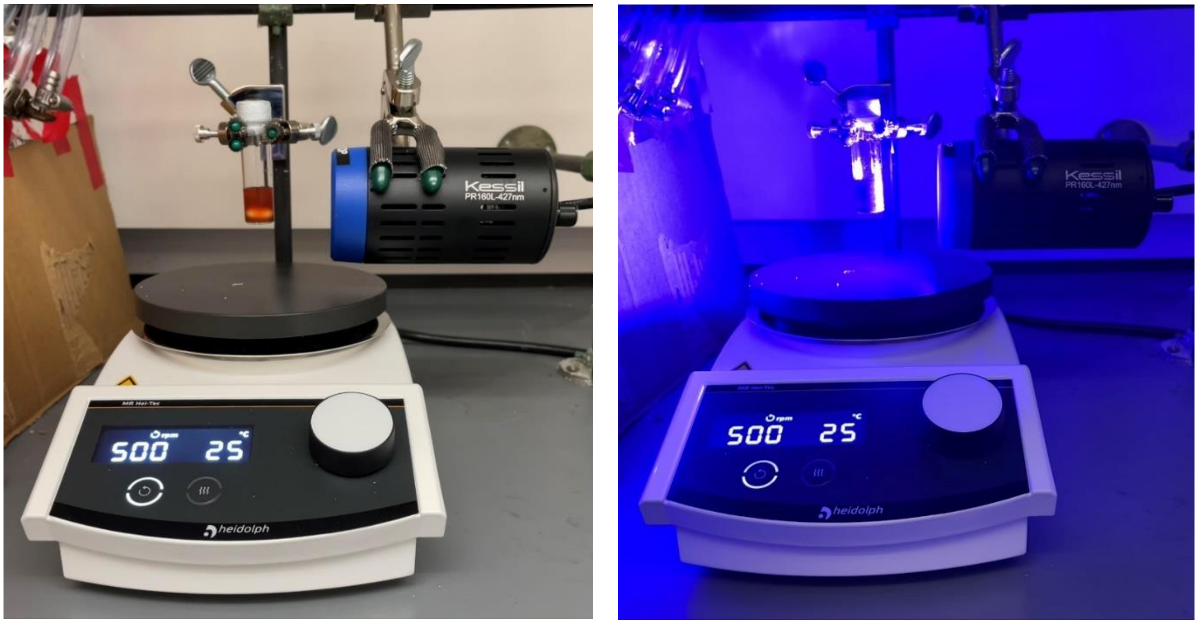

Figure S1: Vitamin B_{12}-Catalyzed Epoxide Ring Opening Reaction Set-Up

Reaction Optimization

General Procedure 1

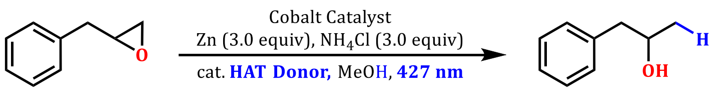

1A **2A**

To an 8 mL septum screw-capped vial equipped with magnetic stir bar, was added (2,3-epoxypropyl)benzene (26.8 mg, 0.2 mmol, 1.0 equiv), Cobalt Catalyst, Zn (39.2 mg, 0.6 mmol, 3.0 equiv), NH_4Cl (32.1 mg, 0.6 mmol, 3.0 equiv), hydrogen atom transfer (HAT) donor, and methanol solvent (2 mL). The resultant mixture was sparged with a nitrogen balloon for 10 min and the vial was sealed with parafilm. The mixture was then stirred and irradiated with a 427 nm LED (Kessil®) for 24 hr. The reaction mixture was then concentrated *in vacuo*, re-suspended in dichloromethane, and sonicated before being passed through a cotton pipette filter. The resulting filtrate was concentrated *in vacuo*. Yield was determined by ^1H NMR analysis using 1,3,5-trimethoxybenzene as an internal standard.

Entry	Cobalt Catalyst	HAT Donor	Yield (%)
1	Vitamin B_{12} (5 mol %)	TRIP disulfide (10 mol %)	77
2	Vitamin B_{12} (5 mol %)	TRIP disulfide (5 mol %)	68
3	Vitamin B_{12} (1 mol %)	TRIP disulfide (10 mol %)	65
4	Vitamin B_{12} (1 mol %)	TRIP disulfide (5 mol %)	**87**
5[c]	Vitamin B_{12} (1 mol %)	Diphenyl disulfide (5 mol %)	52
6	Vitamin B_{12} (1 mol %)	TRIP disulfide (1 mol %)	73
7	Vitamin B_{12} (1 mol %)	TRIP thiol (10 mol %)	**84**
8	Cobalt(II)(salen) (1 mol %)	TRIP thiol (10 mol %)	48
9	Cobalt(II)(salen) (10 mol %)	TRIP thiol (10 mol %)	66
10[a]	none	TRIP thiol (10 mol %)	trace
11[a]	Vitamin B_{12} (1 mol %)	none	trace
12[a,b]	Vitamin B_{12} (1 mol %)	TRIP thiol (10 mol %)	trace

[a]Control reaction. [b]No irradiation. [c]See **Scheme S1** below.

Table S1. Summary of Cobalt Catalyst, HAT Donor, and Catalytic Loading Optimization, including Control Reactions

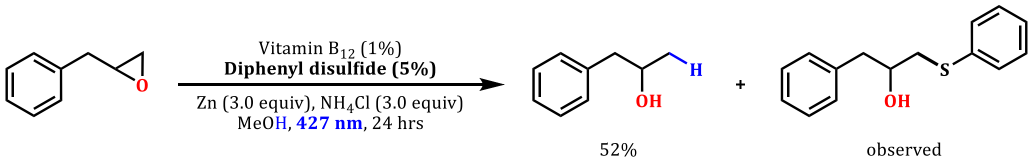

Scheme S1. Diphenyl Disulfide Acts as Competitive Nucleophile for Epoxide Ring Opening

Mechanistic Considerations

TEMPO Radical Inhibition Experiment

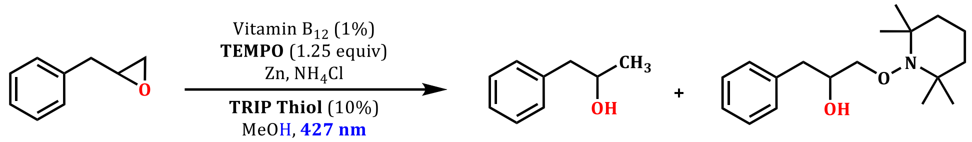

To an 8 mL septum screw-capped vial equipped with magnetic stir bar was added (2,3-epoxypropyl)benzene (53.7 mg, 0.400 mmol, 1.0 equiv), Vitamin B_{12} (5.4 mg, 1 mol %), Zn (78.5 mg, 1.201 mmol, 3.0 equiv), NH_4Cl (64.2 mg, 1.200 mmol, 3.0 equiv), and TEMPO (78.2 mg, 0.500 mmol, 1.25 equiv). 2,4,6-triisopropylbenzene thiol ("TRIP thiol") was then added from a 0.1 mmol/mL stock solution in deuterated methanol (0.4 mL, 10 mol %) followed by addition of the deuterated methanol solvent (3.6 mL). This solution was sparged with a nitrogen balloon for 10 min and the vial was sealed with parafilm. The mixture was then stirred and irradiated with a 427 nm LED (Kessil®) for 48 hr. The reaction mixture was then concentrated *in vacuo*, re-suspended in dichloromethane, and sonicated before being passed through a cotton pipette filter. The filtrate was concentrated *in vacuo*. Yield was determined by ^{1}H NMR analysis using 1,3,5-trimethoxybenzene as an internal standard.

Entry	TEMPO Equiv	Yield (%)
1	0.0	84
2	1.25	41

The significant decrease in yield when TEMPO is present relative to the control is consistent with radical inhibition, and suggests the reaction proceeds through a radical mechanism. However, we are cognizant of the findings of Sen and coworkers demonstrating that non-radical reactions can be inhibited by TEMPO, showing that these results must be interpreted with care.[1]

TEMPO- and Cobalt(III)-Adduct Detection Chromatograms

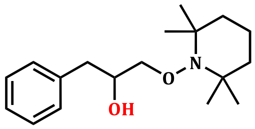

detected
m/z 292.2376
calc for $[M+H]^+$ 292.2272

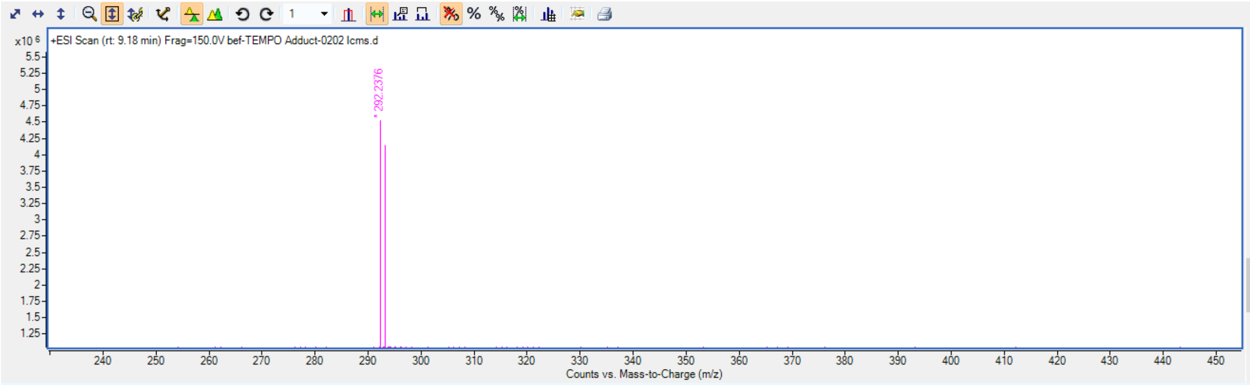

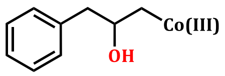

detected
m/z 1465.8
calc for $[M+H]^+$ 1465.6570

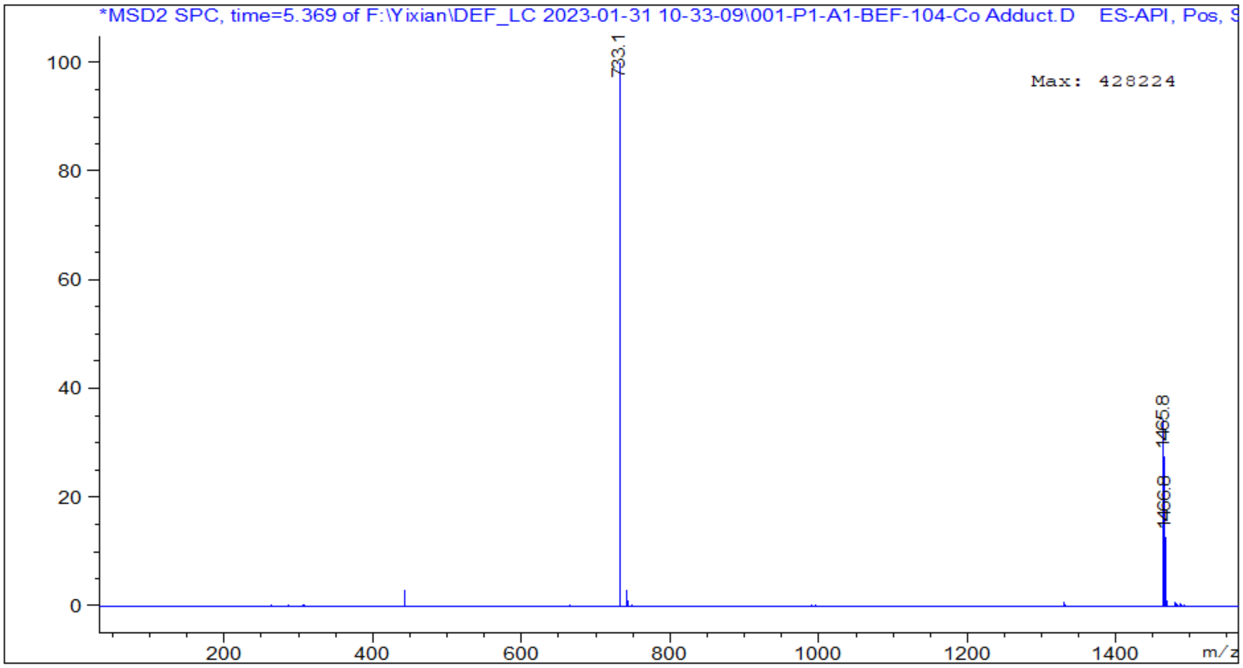

Radical Clock Experiment

To further investigate the proposed radical mechanism of our reaction, a radical clock experiment was conducted with 1,2-epoxy-5-hexene as the substrate. If indeed a radical is formed at the alpha position to the alcohol upon light-mediated homolytic cleavage of a Co-C bond, the intermediate species would be primed to undergo a 5-exo-trig and/or 6-endo-trig radical cyclization because of its position relative to the olefin in the starting material.

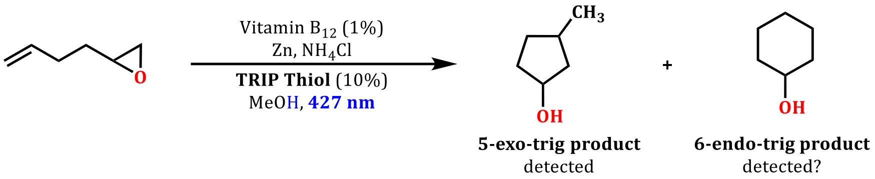

To an 8 mL septum screw-capped vial equipped with magnetic stir bar was added 1,2-epoxy-5-hexene (19.7 mg, 0.201 mmol, 1.0 equiv), Vitamin B_{12} (cyanocobalamin, 2.7 mg, 1 mol %), Zn (39.2 mg, 0.599 mmol, 3.0 equiv), and NH_4Cl (32.1 mg, 0.600 mmol, 3.0 equiv). 2,4,6-triisopropylbenzene thiol ("TRIP thiol") was then added from a 0.1 mmol/mL stock solution in deuterated methanol (0.2 mL, 10 mol %) followed by addition of the deuterated methanol solvent (1.8 mL). This solution was sparged with a nitrogen balloon for 10 min and the vial was sealed with parafilm. The mixture was then stirred and irradiated with a 427 nm LED (Kessil®) for 48 hr. The reaction mixture was then concentrated *in vacuo*, suspended in dichloromethane, and sonicated before being passed through a cotton pipette filter. The filtrate was concentrated *in vacuo* and the crude product was analyzed by GC-MS (**Figure S2**).

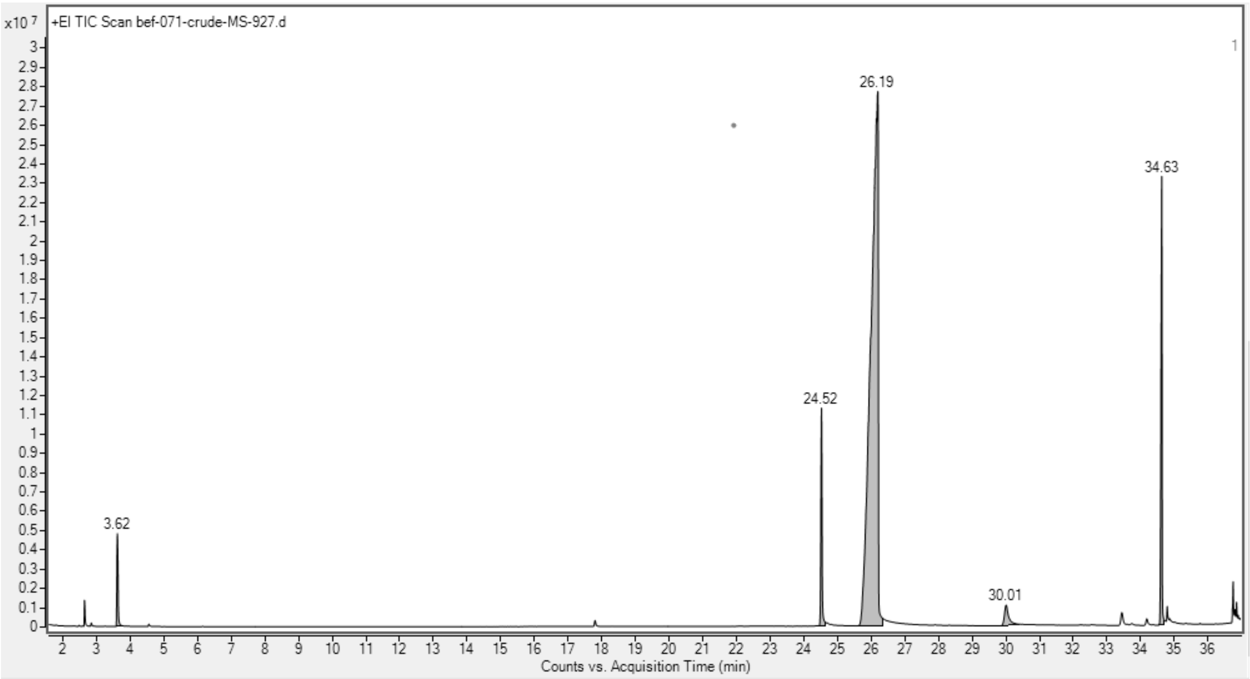

Figure S2. GC-MS Chromatogram of Radical Clock Experiment

Notably, the signal at t = 3.617 min displayed a mass spectrum (**Figure S3**) that very closely resembles (73% match with the NIST mass spectrum library of organic molecules) that of 3-methylcyclopentanol (**Figure S4**).[2] This strongly suggests that 1) a radical is generated at the terminal carbon of the epoxide substrate during the reaction mechanism, and 2) the 5-exo-trig radical cyclization product, 3-methycyclopropanol is present in the crude reaction mixture when 1,2-epoxy-5-hexene is subjected to our epoxide ring-opening reaction.

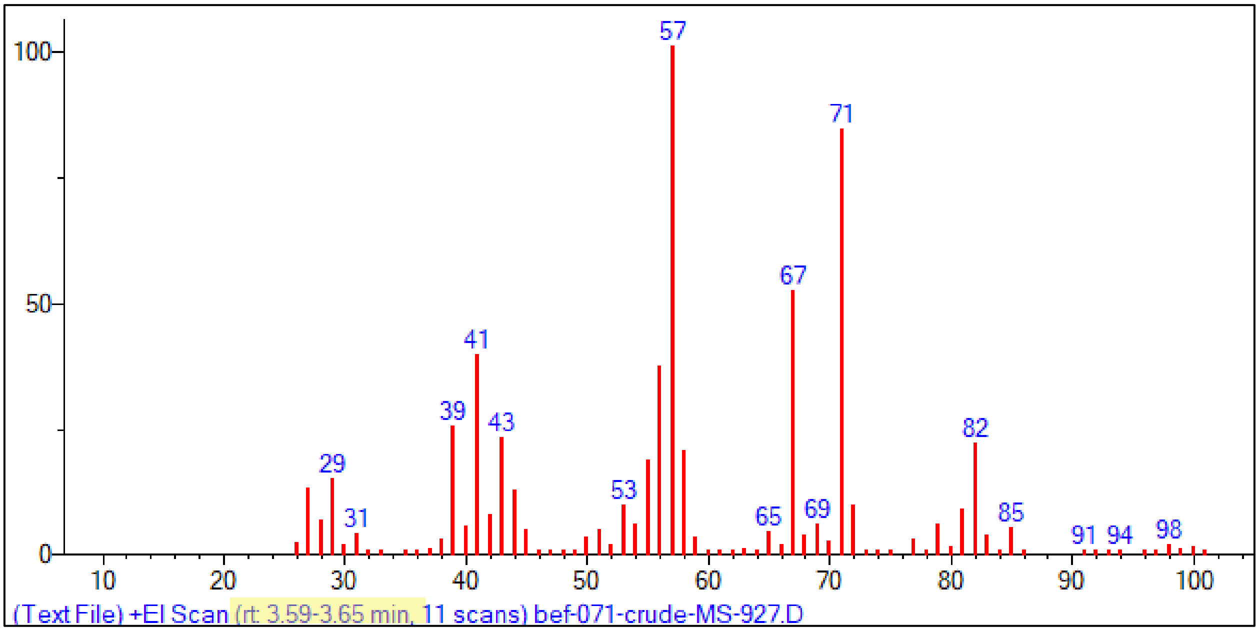

Figure S3. Mass Spectrum of Component Acquired at 3.617 Minutes

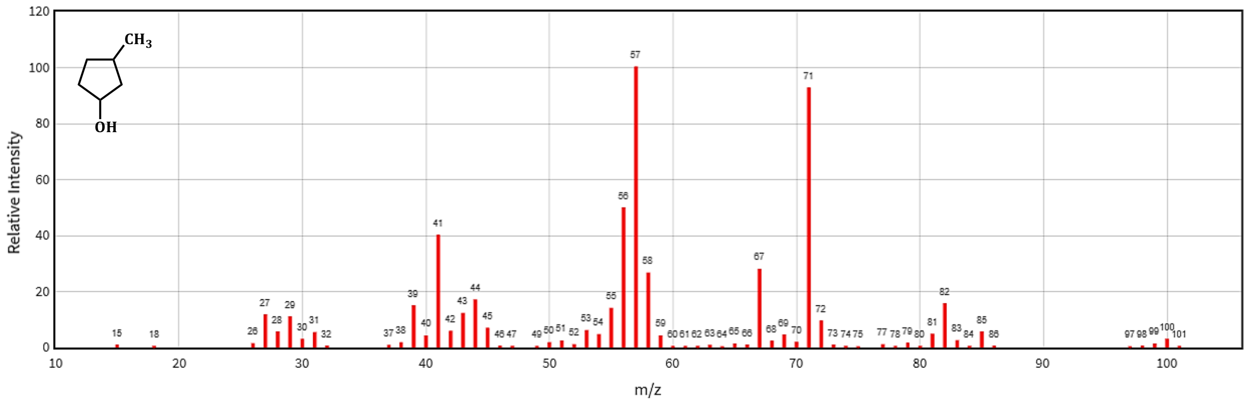

Figure S4. Mass Spectrum of 3-Methylcyclopropanol, adapted from NIST WebBook [2]

Additionally, a standard of cyclohexanol was analyzed on GC-MS resulting in the chromatogram shown below (**Figure S5**). The standard had an acquisition time of 4.582 minutes. This is significant because a minor signal is present at ~4.57 minutes in the chromatogram of the radical clock reaction (**Figure S6**) which gives reasonable evidence that the 6-endo-trig cyclization byproduct is also present.

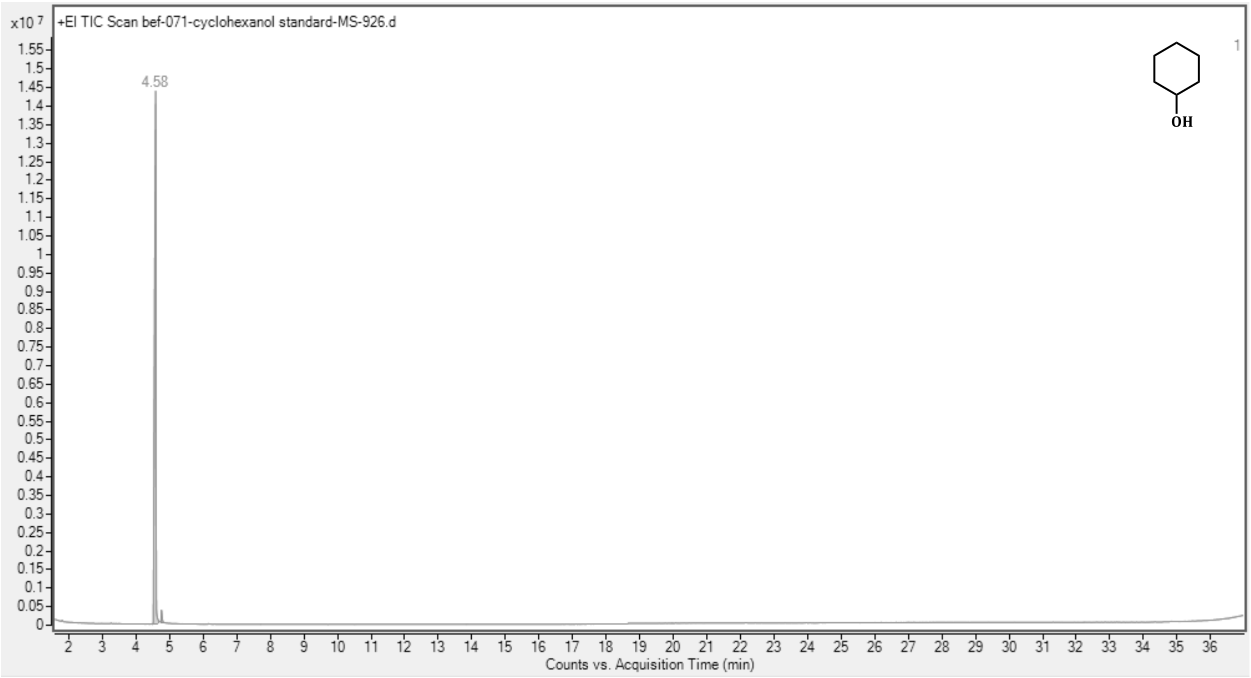

Figure S5. GC-MS Chromatogram of 1-Cyclohexanol

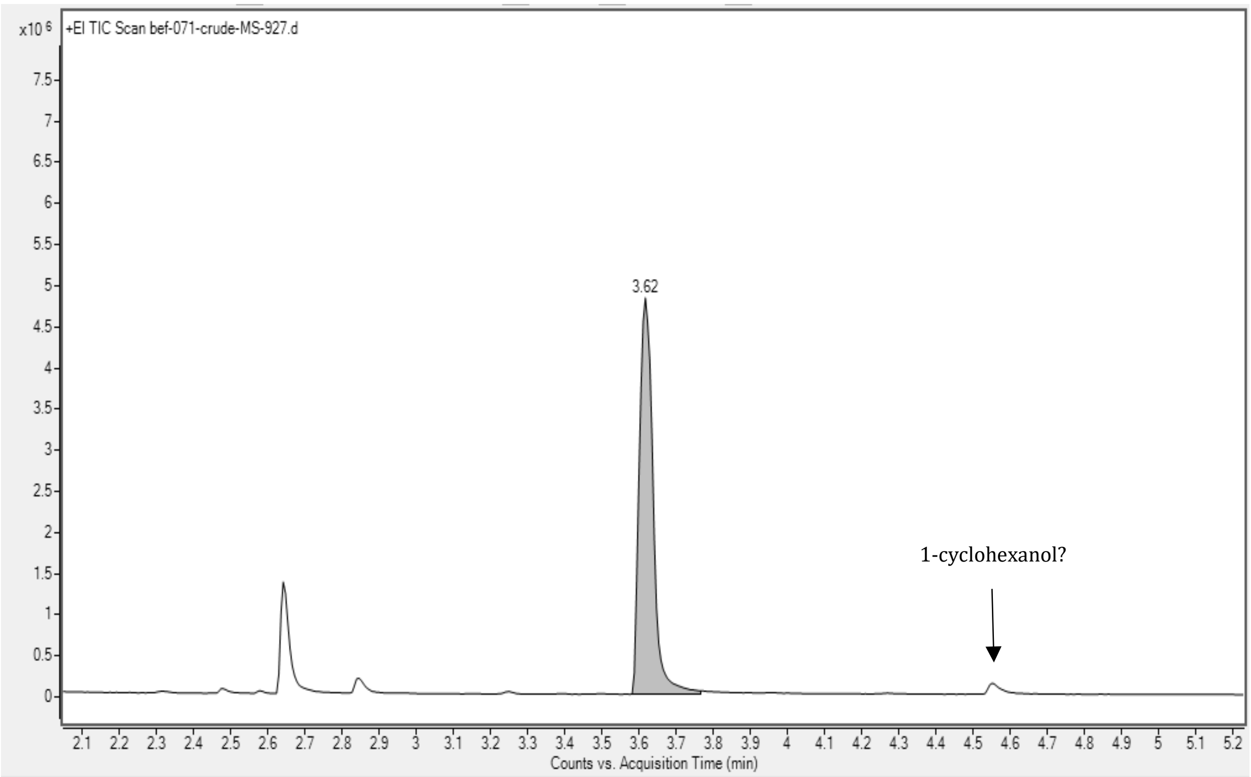

Figure S6. GC-MS Chromatogram of Radical Clock Experiment (Zoomed)

Deuterium Labeling Experiment

We wondered if perhaps the methanol solvent was active in replenishing the thiol with hydrogen to continue the catalytic HAT process, as opposed to solely being supplied by NH_4Cl, the strongest acid in the reaction system. To gain some clarity in this matter, we sought to run the control reaction in deuterated methanol. Presumably if the methanol solvent was serving as a proton source in the HAT catalytic cycle, we would see deuterium incorporation of the product when reacted in a deuterated methanol solvent.

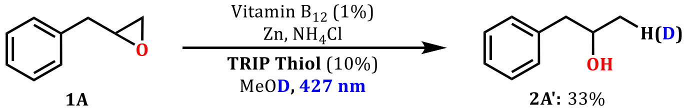

1A **2A'**: 33% 58% D

To an 8 mL septum screw-capped vial equipped with magnetic stir bar was added (2,3-epoxypropyl)benzene (54.2 mg, 0.404 mmol, 1.0 equiv), Vitamin B_{12} (cyanocobalamin, 5.4 mg, 1 mol %), Zn (78.5 mg, 1.201 mmol, 3.0 equiv), and NH_4Cl (64.2 mg, 1.200 mmol, 3.0 equiv). 2,4,6-triisopropylbenzene thiol ("TRIP thiol") was then added from a 0.1 mmol/mL stock solution in deuterated methanol (0.4 mL, 10 mol %) followed by addition of the deuterated methanol solvent (3.6 mL). This solution was sparged with a nitrogen balloon for 10 min and the vial was sealed with parafilm. The mixture was then stirred and irradiated with a 427 nm LED (Kessil®) for 24 hr. The reaction mixture was then concentrated *in vacuo*, suspended in dichloromethane, and sonicated before being passed through a cotton pipette filter. The filtrate was concentrated *in vacuo* and purified by silica gel column chromatography (40% ethyl acetate in hexane) to afford the alcohol product (**2A'**) as a pale yellow oil (17.9 mg, 33%).

The NMR spectra of the isolated product (**Figures S7** and **S8**) strongly suggest deuterium incorporation. Relative to the ^1H NMR spectrum of the unlabeled alcohol (**Figure S9**), the methyl proton peak displays a notable decrease in integration 2.42 which indicates approximately 58% deuterium incorporation. Additionally, in the ^{13}C NMR spectrum of the deuterated alcohol, two signals are visible corresponding to the

terminal carbon of the alkyl chain. One signal occurs at 22.8 ppm, identical to that seen in the unlabeled alcohol's ^{13}C NMR (**Figure S10**). However, a 1:1:1 triplet centered at 22.5 ppm is also observed; this is further evidence of partial deuterium incorporation in the alcohol product when the epoxide is reacted in deuterated methanol.

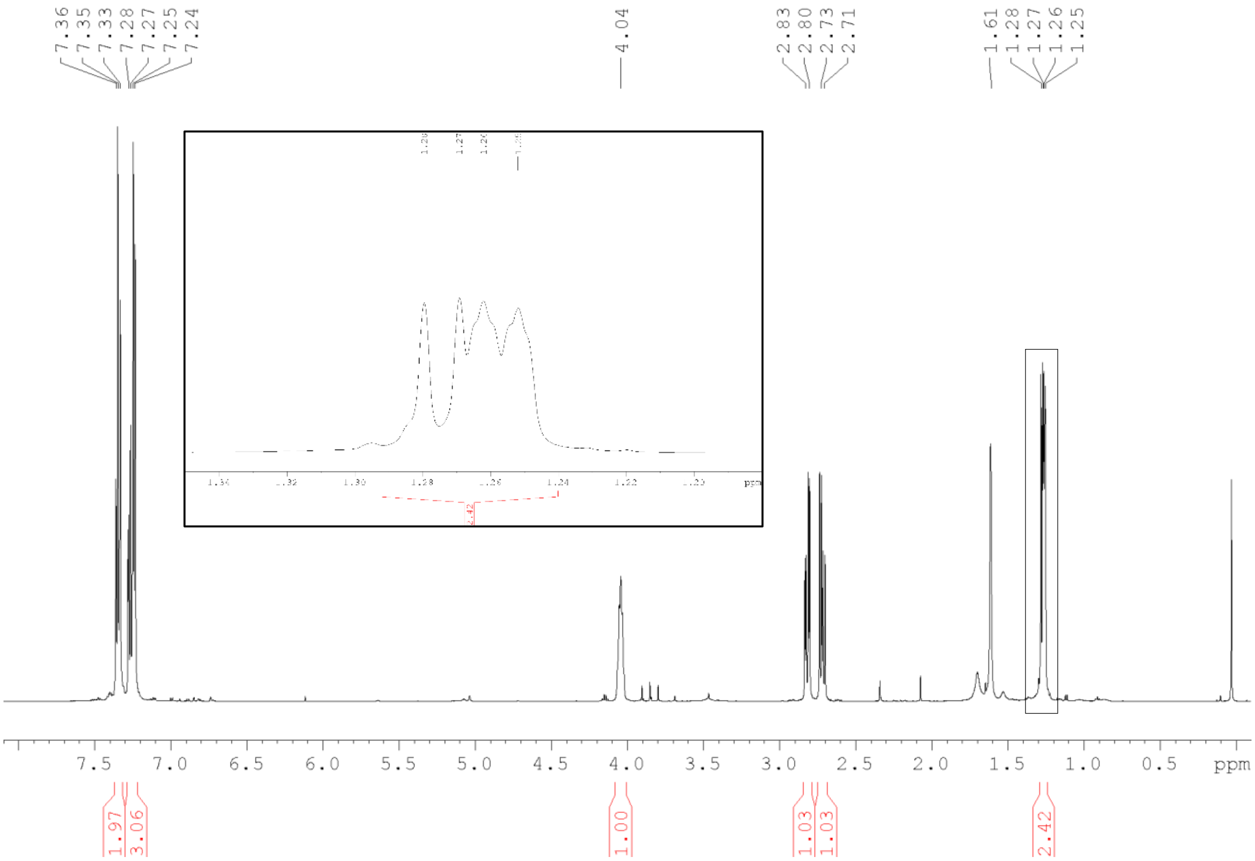

Figure S7. ^{1}H NMR of Deuterium-Labeled 3-Phenyl-2-Propanol (**2A'**)

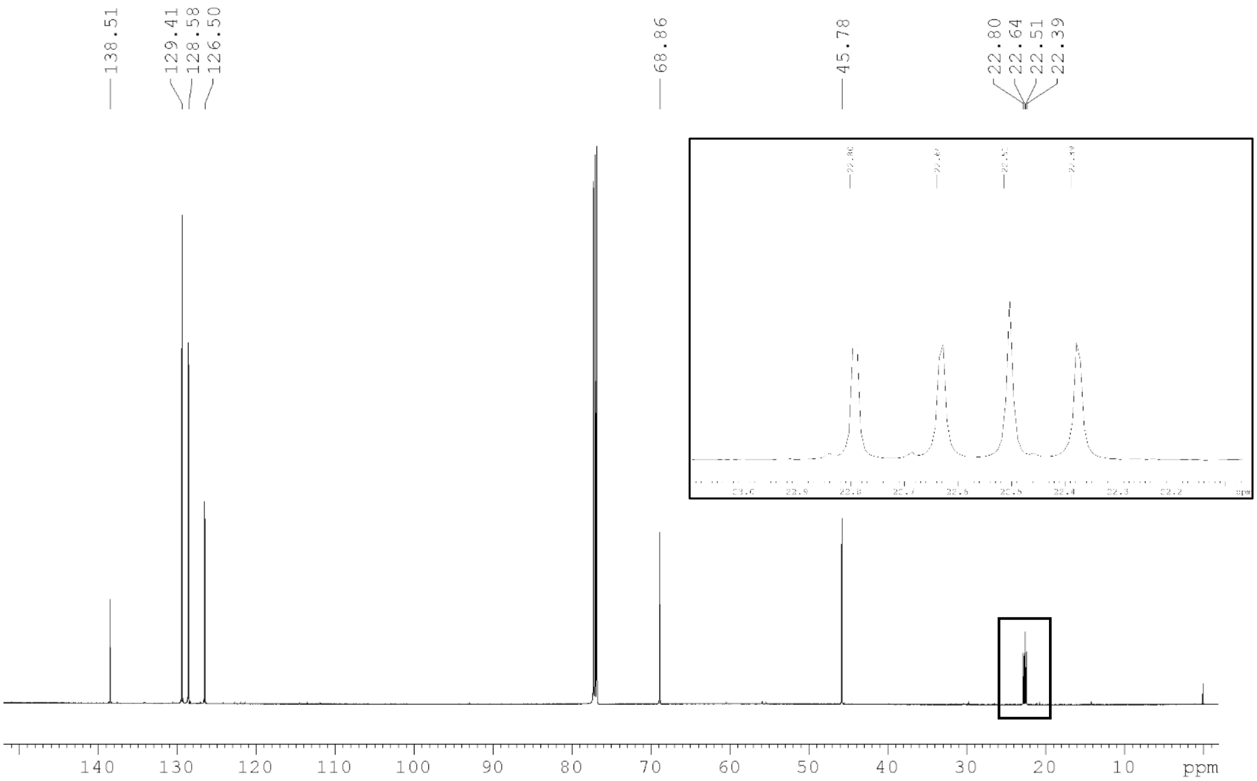

Figure S8. ^{13}C NMR Spectrum of Deuterium-Labeled 3-Phenyl-2-Propanol (**2A'**)

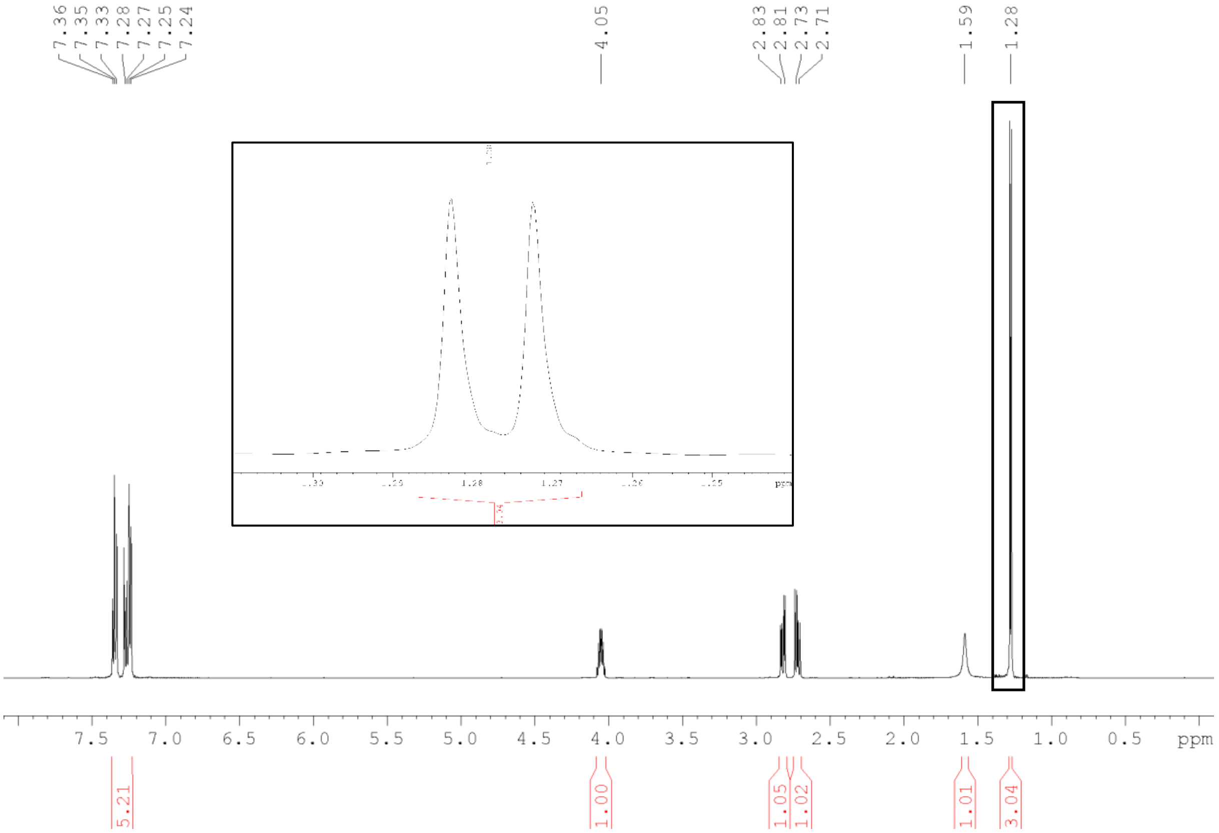

Figure S9. ^{1}H NMR Spectrum of 3-Phenyl-2-Propanol (**2A**)

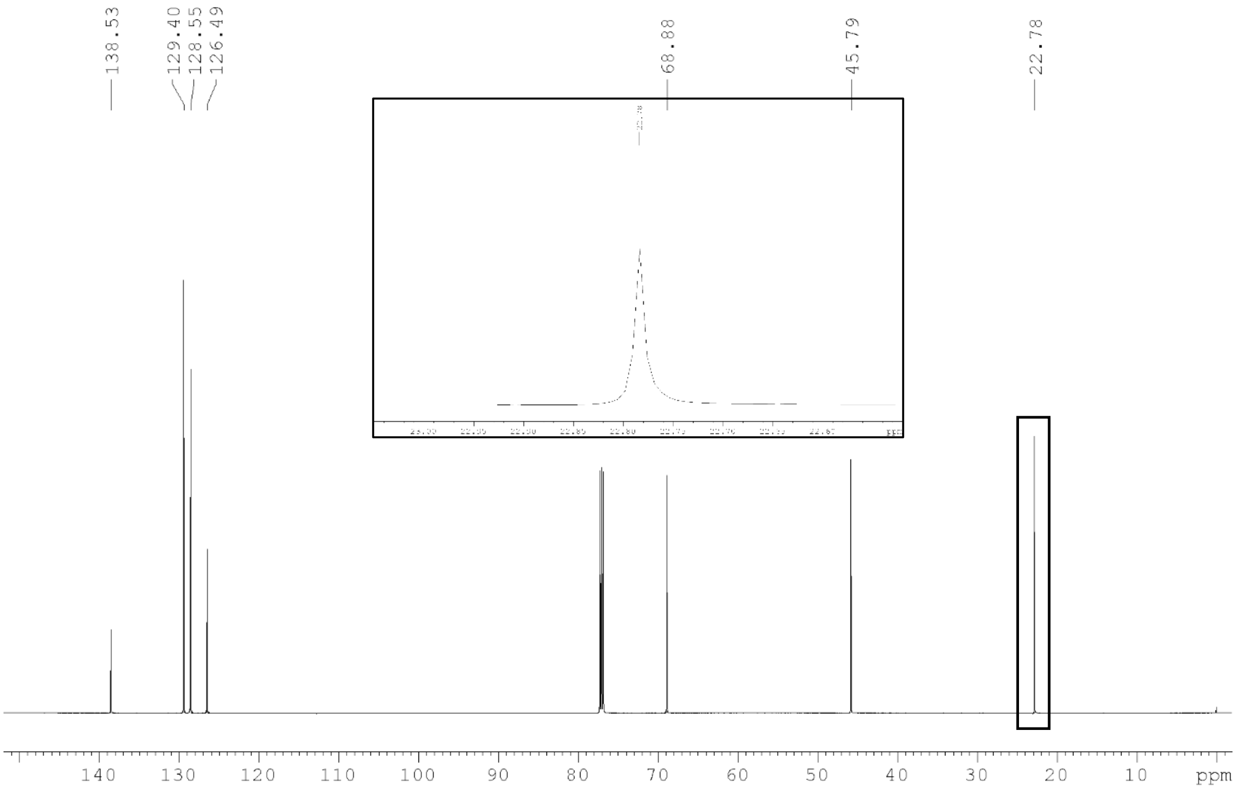

Figure S10. ^{13}C NMR Spectrum of 3-Phenyl-2-Propanol (**2A**)

Mass Balance Studies for Select Scope Compounds

For each selected epoxide substrate: Epoxide (0.4 mmol, 1.0 equiv), Vitamin B_{12} (5.4 mg, 0.004 mmol, 1 mol %), Zn (78.4 mg, 1.2 mmol, 3.0 equiv), and NH_4Cl (64.2 mg, 1.2 mmol, 3.0 equiv) were added to an 8-mL septa-capped vial fitted with a stir bar. 2,4,6-triisopropylbenzene thiol ("TRIP thiol") was then added from a 0.1 mmol/mL stock solution in methanol (0.4 mL, 0.04 mmol, 10 mol %) followed by the methanol solvent (3.6 mL). Additionally, 1,2,4,5-tetramethylbenzene (arbitrary amount) was added for use as an internal standard (^{1}H NMR δ 6.89 ppm, 2H; 2.18 ppm, 12H). This solution was sparged with a nitrogen balloon for 10 min and the vial was sealed with parafilm. The mixture was then stirred and irradiated with a 427 nm LED (Kessil®) for 1-30 hrs.

For each time point, a 100 µL aliquot was taken from the reaction mixture and dissolved in 800 µL of $CDCl_3$. This solution was extracted with 3 mL of H_2O; the organic layer was removed and used directly to acquire a ^{1}H NMR spectrum.

Reaction of 2,3-epoxypropylbenzene (1A)

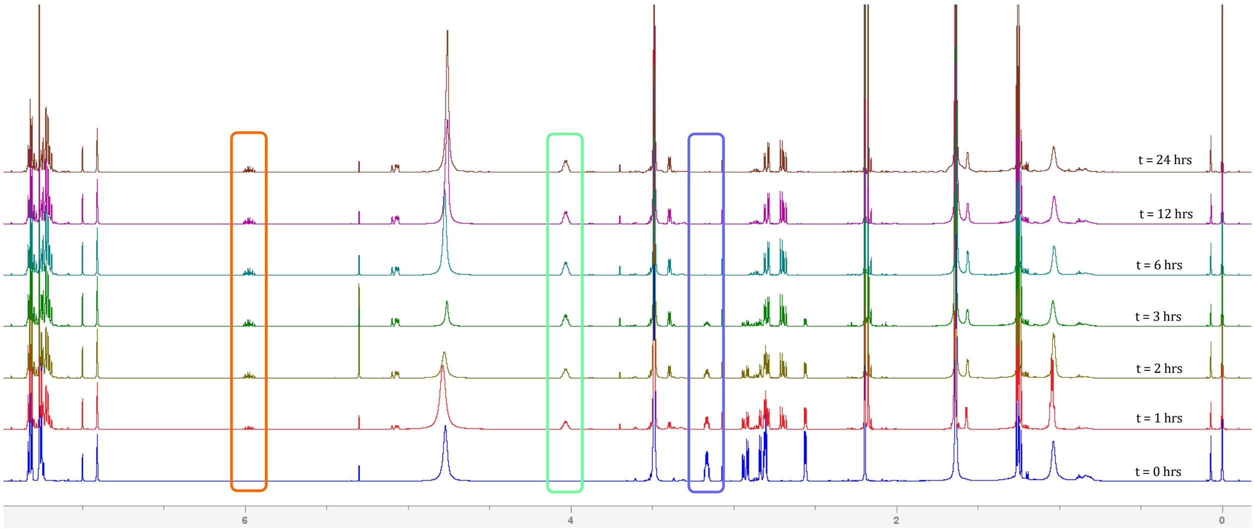

Time (hrs)	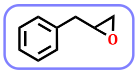	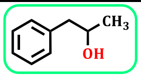	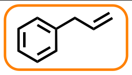
0	98.3%	0	0
1	44.5%	50.5%	4.9%
2	29.2%	54.5%	14.3%
3	14.2%	64.3%	16.5%
6	0	76.7%	18.6%
12	0	76.8%	18.7%
24	0	77.2%	18.5%

Reaction of 2-phenyl oxirane (1C)

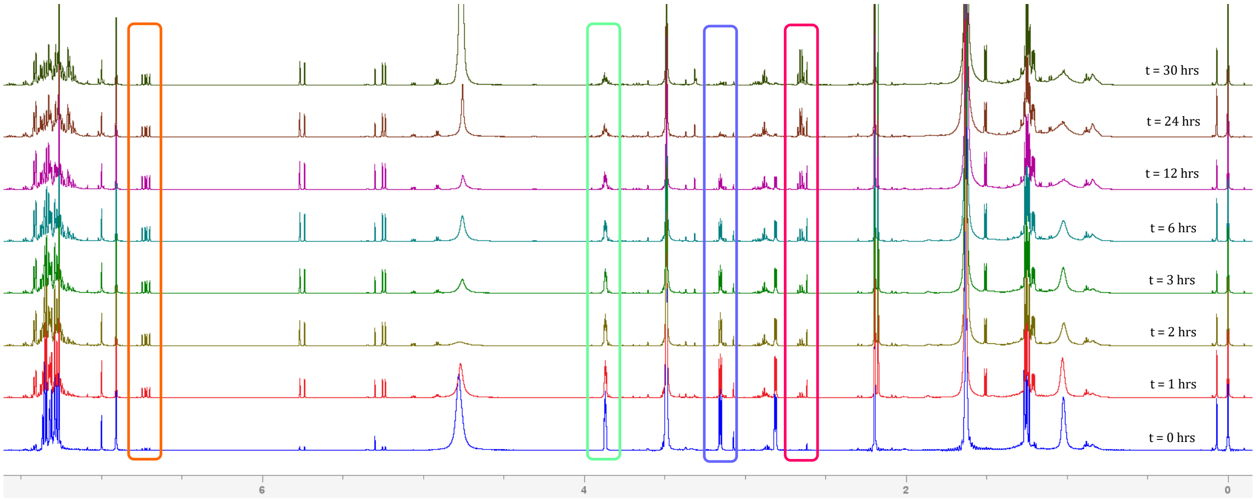

Time (hrs)	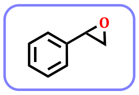	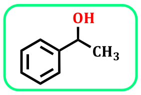	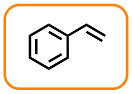	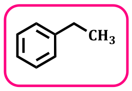
0	95.3%	0	4.7%	0
1	58.2%	9.9%	21.2%	4.1%
2	44.8%	9.5%	26.2%	5.8%
3	39.3%	9.7%	29.7%	7.6%
6	29.7%	10.7%	31.6%	10.6%
12	18.1%	9.6%	31.1%	15.6%
24	5.8%	14.5%	27.2%	26.6%
30	4.5%	13.1%	24.6%	29.9%

Reaction of *o*-acetyleugenol oxide (1E)

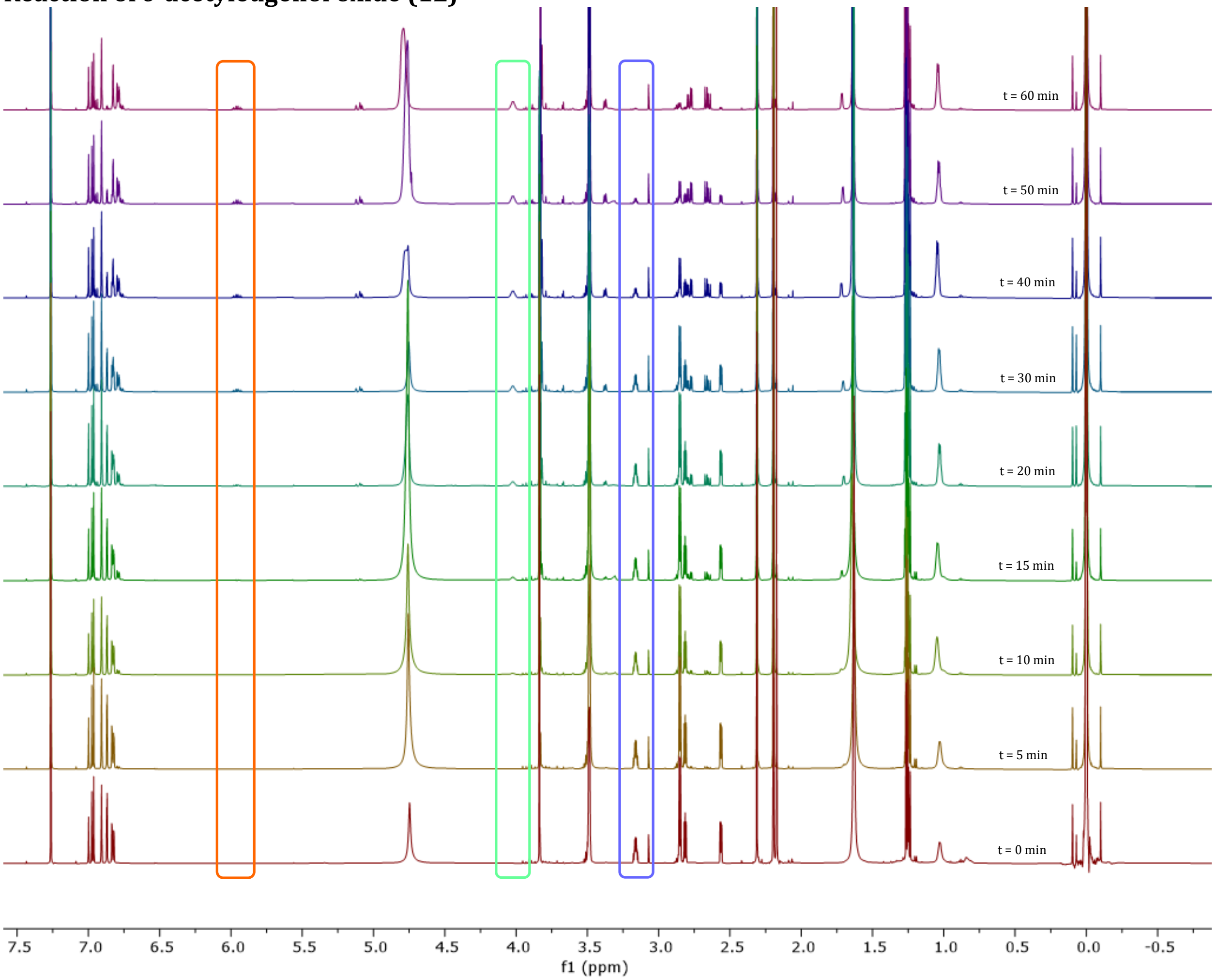

Time (min)	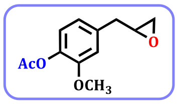	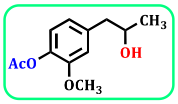	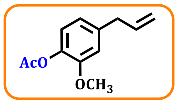
0	100%	0	0
5	93.3%	5.1%	0
10	85.7%	12.0%	0
15	75.4%	20.2%	0
20	65.3%	24.1%	1.4%
30	50.8%	36.1%	5.5%
40	33.3%	47.4%	8.8%
50	19.0%	56.0%	10.1%
60	7.0%	69.1%	13.0%

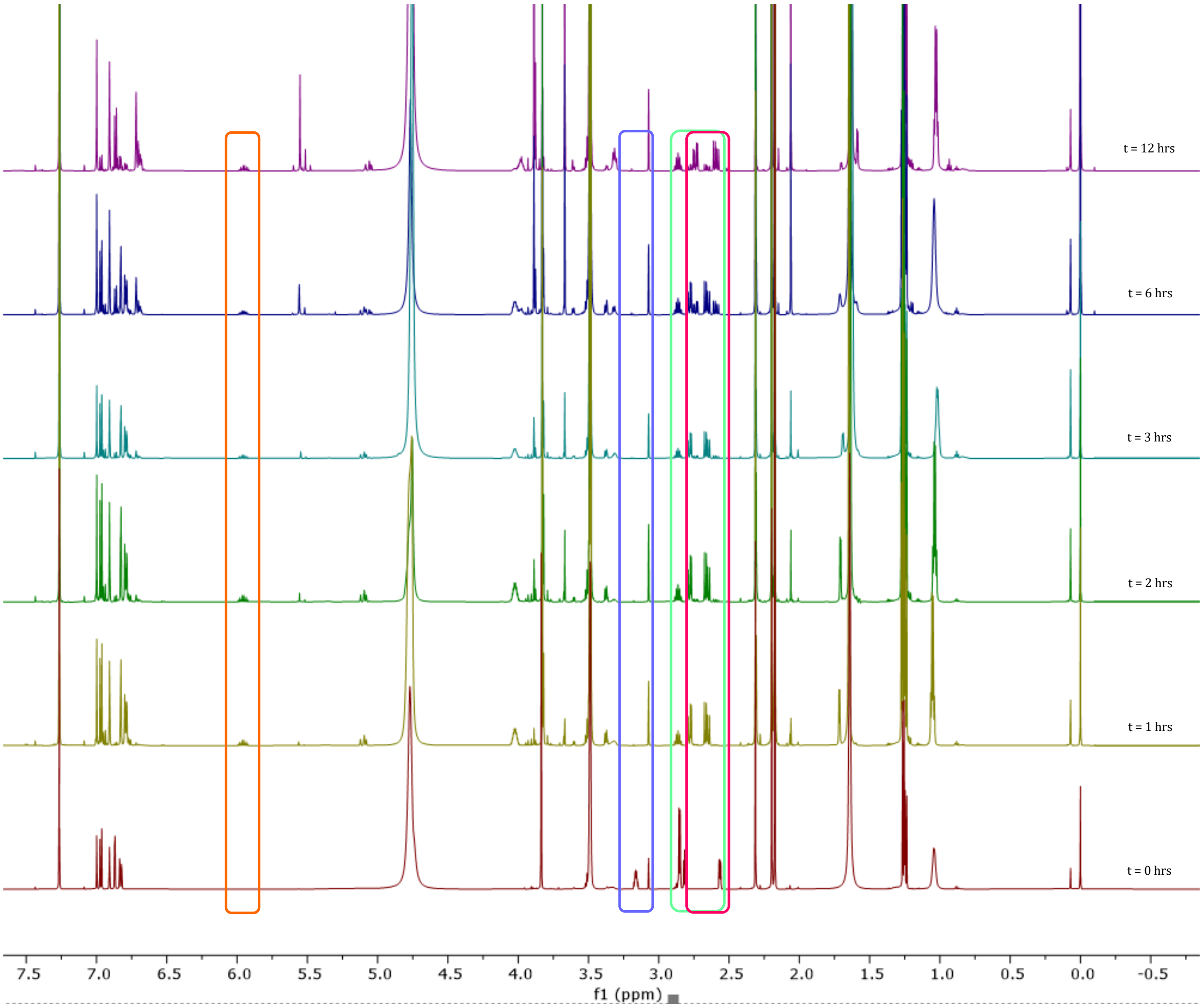

Time (hrs)	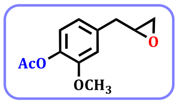	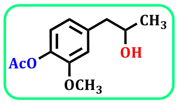	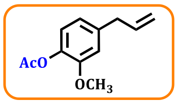	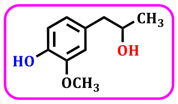
0	100%	0	0	0
1	0	75.7%	9.1%	1.1%
2	0	74.3%	10.2%	3.3%
3	0	66.7%	4.4%	5.9%
6	0	20.2%	8.0%	49.5%
12	0	9.0%	7.7%	41.1%

Reaction of benzyl-10,11-epoxy-undecanoate (1K)

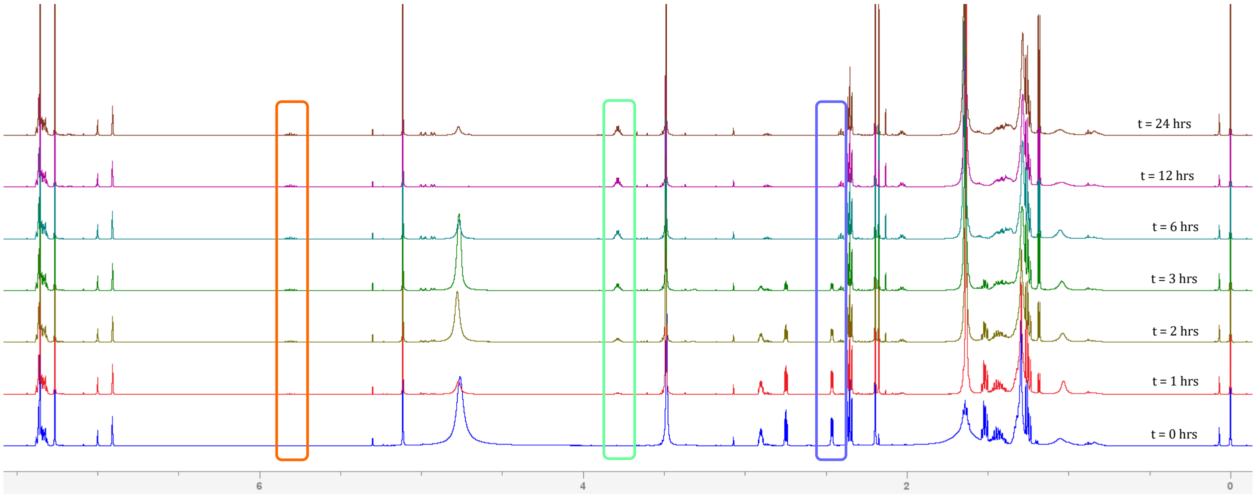

Time (hrs)	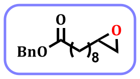	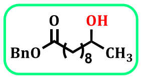	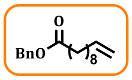
0	100%	0	0
1	82.4%	12.9%	4.7%
2	58.6%	34.6%	8.3%
3	29.1%	59.6%	10.7%
6	0	81.3%	13.8%
12	0	76.8%	13.4%
24	0	76.3%	13.4%

Reaction of 1-(oxiran-2-ylmethyl)-1H-indole (1O)

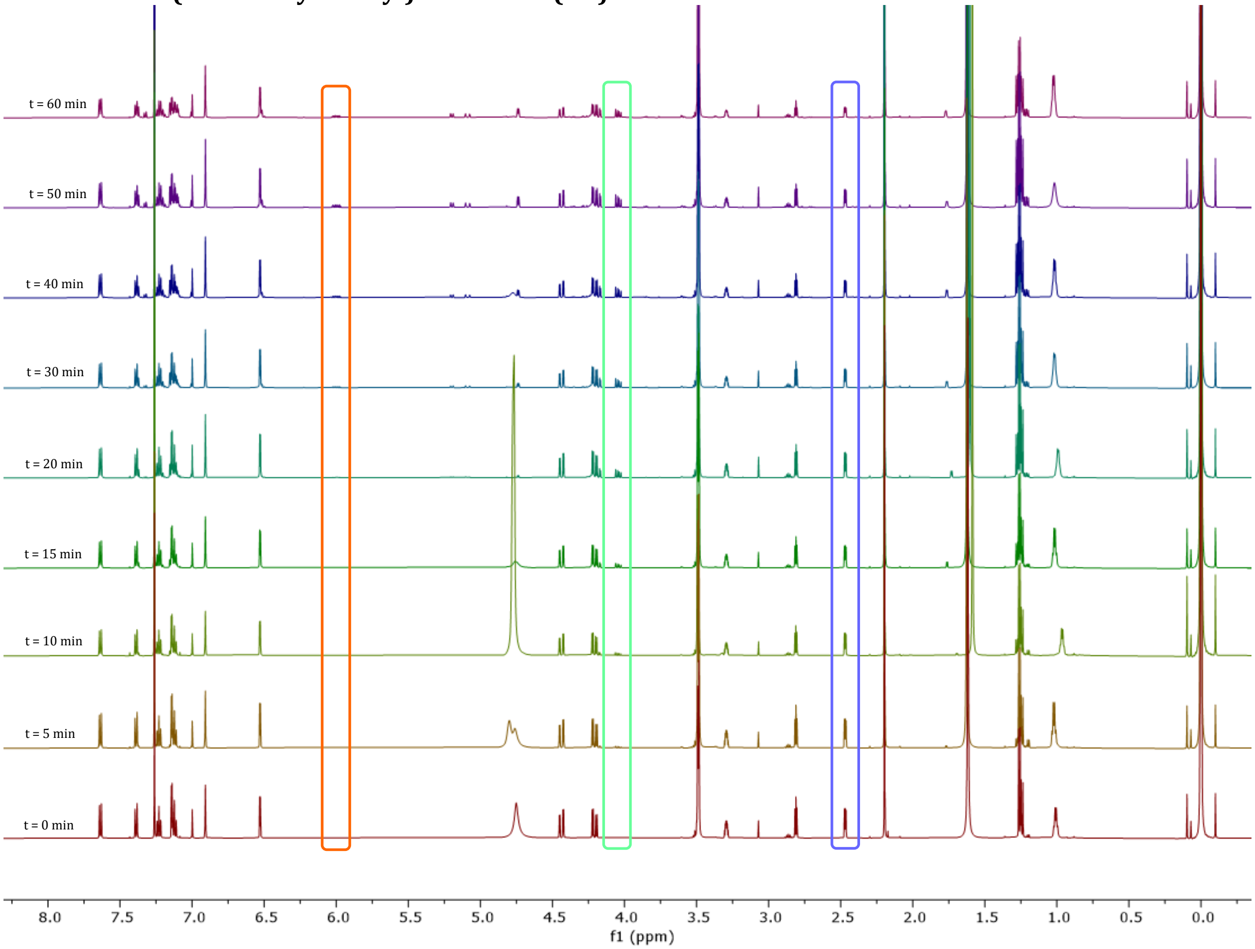

Time (min)	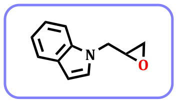	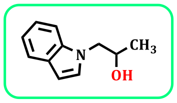	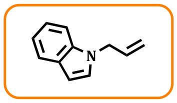
0	100%	0	0
5	94.0%	5.7%	0
10	90.0%	10.8%	0
15	79.2%	16.3%	0
20	69.6%	21.5%	2.0%
30	58.4%	26.4%	5.5%
40	51.0%	29.2%	8.7%
50	45.5%	29.9%	12.3%
60	40.1%	31.3%	15.1%

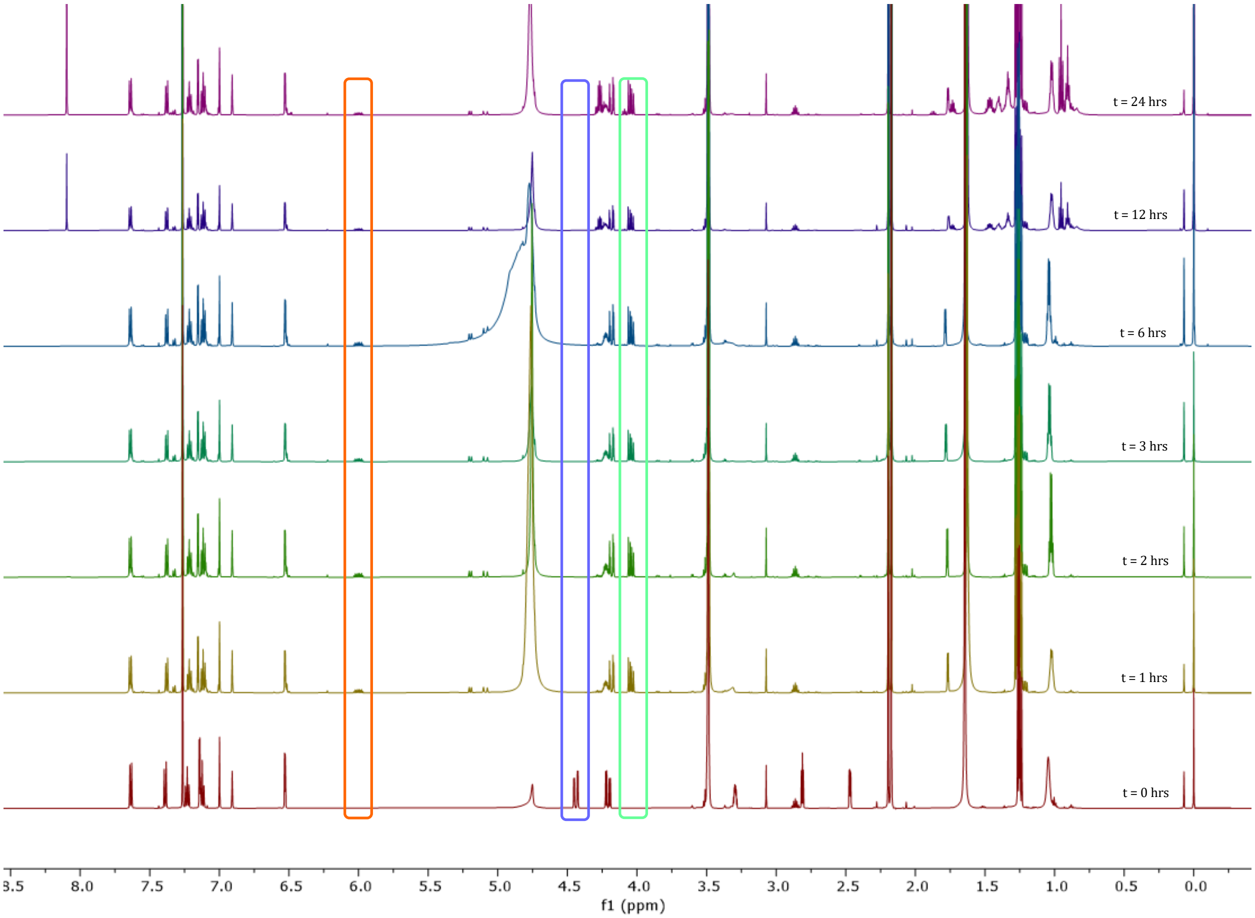

**Time (hr)**	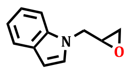	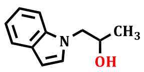	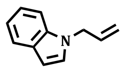
0	100%	0	0
1	0	74.3%	10.4%
2	0	74.0%	12.1%
3	0	68.7%	10.9%
6	0	74.9%	12.0%
12	0	70.9%	8.8%
24	0	74.7%	6.9%

Syntheses of Epoxide and Oxetane Substrates

General Procedure 2

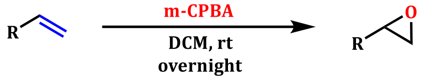

3-chloroperbenzoic acid (2.5 equiv) was added to a 150-mL round bottom flask equipped with stir bar and dissolved in dichloromethane (15 mL). The flask was sealed with a rubber septum and equipped with a vent needle. In a separate round bottom flask, alkene (1.0 equiv) was dissolved in dichloromethane (10 mL). The alkene/DCM solution was slowly added (over ~1 min) to the *m*-CPBA mixture via syringe and the reaction was allowed to stir overnight. The reaction was quenched with sat. $NaHCO_3$ and allowed to stir for 20 min and then extracted with dichloromethane. The combined organic layers were washed with brine, dried over Na_2SO_4, filtered and concentrated *in vacuo*. The epoxides were isolated using silica gel column chromatography.

1,2-epoxy-4-phenylbutane (1B)

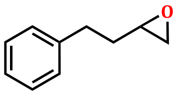

1B

4-phenyl-1-butene (787.8 mg, 5.96 mmol) was reacted according to **General Procedure 2.** Purification by silica gel column chromatography (20% ethyl acetate in hexane) afforded epoxide **1B** (680.5 mg, 76%) as a colorless oil. ^{1}H NMR (600 MHz, $CDCl_3$): δ 7.31-7.28 (m, 2H), 7.23-7.18 (m, 3H), 2.98-2.94 (m, 1H), 2.86-2.80 (m, 1H), 2.79-2.72 (m, 2H), 2.48 (dd, J = 4.8, 2.7 Hz, 1H), 1.92-1.80 (m, 2H); ^{13}C NMR (151 MHz, $CDCl_3$): δ 141.3, 128.5, 128.4, 126.0, 51.8, 47.3, 34.3, 32.3. The spectra are consistent with those reported in literature.[3]

Estragole oxide (1D)

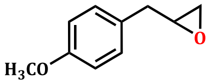

1D

Estragole (502.3 mg, 3.39 mmol) was reacted according to **General Procedure 2.** Purification by silica gel column chromatography (20% ethyl acetate in hexane) afforded epoxide **1D** (418.6 mg, 75%) as a colorless oil. ^{1}H NMR (600 MHz, $CDCl_3$) δ 7.20-7.14 (m, 2H), 6.86 (d, J = 8.6 Hz, 2H), 3.80 (s, 3H), 3.14-3.10 (m, 1H), 2.87 (dd, J = 14.6, 5.6 Hz, 1H), 2.83-2.74 (m, 2H), 2.54 (dd, J = 5.0, 2.6 Hz, 1H); ^{13}C NMR (151 MHz, $CDCl_3$) δ 158.4, 130.0, 129.1, 113.9, 55.3, 52.7, 46.8, 37.8. The spectra are consistent with those reported in literature.[4]

O-acetyleugenol oxide (1E)

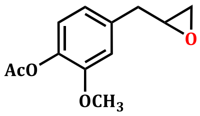

1E

Eugenol acetate (490.9 mg, 2.38 mmol) was reacted according to **General Procedure 2.** Purification by silica gel column chromatography (40% ethyl acetate in hexane) afforded epoxide **1E** (412.6 mg, 78%). ^{1}H NMR (600 MHz, $CDCl_3$) δ 6.99 (d, J = 8.0 Hz, 1H), 6.89 (s, 1H), 6.85 (d, J = 7.9 Hz, 1H), 3.86 (s, 3H), 3.18 (dt, J = 5.8, 2.6 Hz, 1H), 2.87 (d, J = 5.5 Hz, 2H), 2.84 (t, J = 4.4 Hz, 1H), 2.59 (dd, J = 5.1, 2.6 Hz, 1H), 2.34 (s, 3H); ^{13}C NMR (151 MHz, $CDCl_3$) δ 169.3, 150.9, 138.4, 136.3, 122.7, 121.1, 113.1, 55.9, 52.4, 46.9, 38.7, 20.7. The spectra are consistent with those reported in literature.[5]

1,2-epoxydecane (1P)

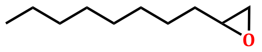

1P

1-decene (846.8 mg, 6.04 mmol) was reacted according to **General Procedure 2**. Purification by silica gel column chromatography (5% ethyl acetate in hexane) afforded epoxide **1P** (407.8 mg, 43% yield) as a colorless oil. ^{1}H NMR (600 MHz, $CDCl_3$): δ 2.92-2.88 (m, 1H), 2.74 (dd, *J* = 4.9, 4.0 Hz, 1H), 2.46 (dd, *J* = 5.1, 2.8 Hz, 1H), 1.56-1.39 (m, 4H), 1.38-1.23 (m, 10H), 0.88 (t, *J* = 7.1 Hz, 3H); ^{13}C NMR (151 MHz, $CDCl_3$): δ 52.4, 47.2, 32.5, 31.9, 29.5, 29.5, 29.2, 26.0, 22.7, 14.1. The spectra are consistent with those reported in literature.[6]

Cyclohexyl-3 epoxy-1,2 propane (1Q)

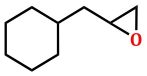

1Q

Allyl cyclohexane (1.003 g, 8.07 mmol,) was reacted according to **General Procedure 2**. Purification by silica gel column chromatography (100% hexane) afforded epoxide **1Q** as a colorless oil (932.3 mg, 82%). ^{1}H NMR (600 MHz, $CDCl_3$) δ 2.99-2.94 (m, 1H), 2.80-2.77 (t, J = 4.6 Hz, 1H), 2.46-2.44 (m, 1H), 1.85-1.63 (m, 5H), 1.57-1.49 (m, 1H), 1.48-1.43 (m, 1H), 1.42-1.37 (m, 1H), 1.31-1.24 (m, 2H), 1.21-1.14 (m, 1H), 1.01-0.96 (m, 2H); ^{13}C NMR (151 MHz, $CDCl_3$) δ 51.1, 47.4, 40.4, 35.9, 33.7, 33.2, 26.4, 26.3, 26.2. The spectra are consistent with those reported in literature.[7]

General Procedure 3

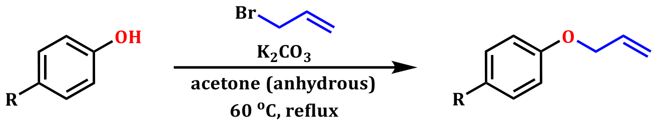

To a 100-mL, two-necked round bottom flask equipped with a magnetic stir bar was added *p*-substituted phenol (1.0 equiv) and potassium carbonate (2.0 equiv). One neck was plugged with a rubber septum and the flask was placed under nitrogen using Schlenk technique. Anhydrous acetone (15 mL) and allyl bromide (1.1 equiv) were added via syringe and the reaction solution was refluxed (oil bath) overnight. After cooling to room temperature, the solvent was concentrated *in vacuo*. The resulting residue was diluted in 30 mL (each) of ethyl acetate and H_2O, then extracted with ethyl acetate. Combined organic layers were washed with brine, dried over Na_2SO_4, filtered, and concentrated *in vacuo*.[8] The resulting crude product was used in **General Procedure 4** without further purification.

General Procedure 4

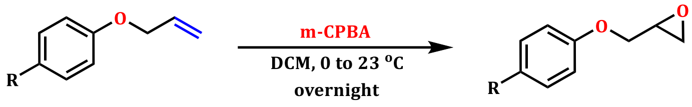

To a 100-mL round bottom flask equipped with a magnetic stir bar was added 3-chloroperbenzoic acid (1.8 equiv) and dichloromethane (15 mL), and the mixture was stirred until *m*-CPBA fully dissolved. The reaction was cooled to 0 °C (ice bath) and allyl ether (crude from **General Procedure 3**) was added. The reaction flask was sealed with a septum/vent needle and stirred overnight. Reaction was quenched with 10 mL H_2O and 10 mL aqueous NaOH (2M), then extracted with dichloromethane. The combined organic layers were washed with brine, dried over Na_2SO_4, filtered, and concentrated *in vacuo*.[8] The epoxide products were purified by silica gel column chromatography.

4-tert-butylphenyl 2,3-epoxypropyl ether (1G)

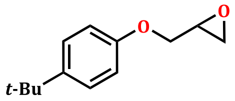

1G

4-tert-butylphenol (1.200 g, 7.98 mmol) was reacted according to **General Procedures 3 and 4**. Purification by silica gel column chromatography (10% ethyl acetate in hexane) afforded epoxide **1G** (1.268 g, 77% yield over 2 steps) as a yellow oil. ^{1}H NMR (600 MHz, $CDCl_3$): δ 7.35-7.31 (m, 2H), 6.91-6.87 (m, 2H), 4.21 (dd, J = 11.0, 3.2 Hz, 1H), 3.99 (dd, J = 10.9, 5.6 Hz, 1H), 3.39-3.35 (m, 1H), 2.92 (t, J = 4.7 Hz, 1H), 2.78 (dd, J = 5.1, 2.8 Hz, 1H), 1.32 (s, 9H); ^{13}C NMR (151 MHz, $CDCl_3$): δ 156.2, 144.0, 126.3, 114.1, 68.8, 50.2, 44.8, 34.1, 31.5. The spectra are consistent with those reported in literature.[9]

4-(2-oxiranylmethoxy) benzonitrile (1H)

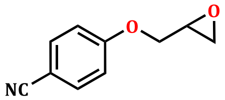

1H

4-cyanophenol (594.8 mg, 4.99 mmol) was reacted according to **General Procedures 3 and 4**. Purification by silica gel column chromatography (20% ethyl acetate in hexane) afforded epoxide **1H** (321.5 mg, 37% yield over 2 steps) as a white solid. ^{1}H NMR (600 MHz, $CDCl_3$): δ 7.62-7.57 (m, 2H), 7.00-6.96 (m, 2H), 4.33 (dd, J = 11.0, 2.8 Hz, 1H), 3.97 (dd, J = 11.1, 6.0 Hz, 1H), 3.39-3.35 (m, 1H), 2.94 (t, J = 4.5 Hz, 1H), 2.77 (dd, J = 4.8, 2.6 Hz, 1H); ^{13}C NMR (151 MHz, $CDCl_3$): δ 161.7, 134.1, 119.1, 115.4, 104.6, 69.1, 49.8, 44.5. The spectra are consistent with those reported in literature.[10]

2-((4-(trifluoromethyl)phenoxy)methyl) oxirane (1I)

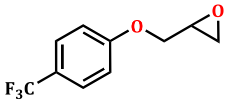

1I

4-trifluoromethylphenol (810.6 mg, 5.0 mmol) was reacted according to **General Procedures 3 and 4**. Purification by silica gel column chromatography (20% ethyl acetate in hexane) afforded epoxide **1I** (363.3 mg, 33% yield over 2 steps) as a faintly yellow oil. ^{1}H NMR (600 MHz, $CDCl_3$): δ 7.60-7.55 (m, 2H), 7.04-6.99 (m, 2H), 4.32 (dd, J = 11.0, 2.9 Hz, 1H), 4.00 (dd, J = 11.0, 5.8 Hz, 1H), 3.42-3.38 (m, 1H), 2.95 (t, J = 4.5 Hz, 1H), 2.80 (dd, J = 4.9, 2.7 Hz, 1H); ^{13}C NMR (151 MHz, $CDCl_3$): δ 160.9, 127.0 (q, J_{C-F} = 3.4 Hz), 124.3 (q, J_{C-F} = 271.0 Hz), 123.4 (q, J_{C-F} = 32.6 Hz), 114.6, 68.9, 49.9, 44.6; ^{19}F NMR (594 MHz, $CDCl_3$): δ -61.5. The spectra are consistent with those reported in literature.[10]

Synthesis of benzyl-10,11-epoxy-undecanoate (1K)

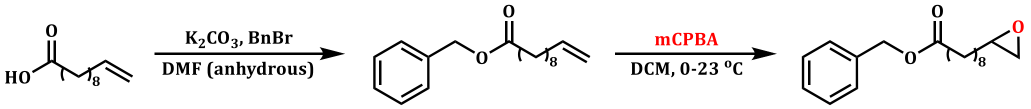

S1 **S2** **1K**

Undec-10-en-1-oic acid (**S1**, 1.843 g, 10.0 mmol, 1.0 equiv), K_2CO_3 (1.658 g, 12.0 mmol, 1.2 equiv), and benzyl bromide (1.1 mL, 9.3 mmol, 0.93 equiv) were added to a 50-mL two-necked round bottom flask equipped with a magnetic stir bar. One neck was plugged with a rubber septum, and the flask was placed under vacuum and flushed with nitrogen (Schlenk technique). The reagents were then dissolved in anhydrous DMF (22 mL) and stirred at room temperature overnight. The reaction was quenched with 1M HCl (15 mL), then extracted

with hexane. The combined organic phase was dried over $MgSO_4$, filtered, and concentrated *in vacuo*. The crude product was used without further purification.

In a 100-mL round bottom flask equipped with magnetic stir bar, benzyl undec-10-en-1-oate (**S2**, crude from previous step) was dissolved in DCM (50 mL) and stirred at 0 ^oC, then *m*-CPBA (2.243 g, 13.0 mmol, 1.3 equiv) was added slowly and the reaction was allowed to warm to room temperature and stirred overnight. The reaction was quenched with 1M NaOH (50 mL) and extracted DCM. The combined organic phase was dried over $MgSO_4$, filtered, and concentrated *in vacuo*. The product (**1K**) was obtained without further purification as a clear oil (2.421 g, 83% over 2 steps). ^1H NMR (600 MHz, $CDCl_3$) δ 7.41-7.32 (m, 5H), 5.11 (s, 2H), 2.93-2.87 (m, 1H), 2.75 (t, *J* = 4.6 Hz, 1H), 2.48-2.45 (m, 1H), 2.35 (t, *J* = 7.5 Hz, 2H), 1.66-1.62 (m, 2H), 1.56-1.49 (m, 2H), 1.47-1.40 (m, 2H), 1.38-1.23 (m, 8H); ^{13}C NMR (151 MHz, $CDCl_3$) δ 173.7, 136.1, 128.6, 128.2, 66.1, 52.4, 47.2, 34.3, 32.5, 29.3, 29.3, 29.1, 29.1, 25.9, 24.9. The spectra are consistent with those reported in literature.[11]

General Procedure 5

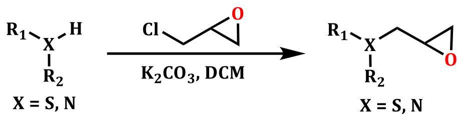

In a 100-mL round bottom flask equipped with a magnetic stir bar, X-H substrate (1.0 equiv) was dissolved in dichloromethane (30 mL). To this mixture was added K_2CO_3 (1.5 equiv) and epichlorohydrin (1.5 equiv). The reaction was allowed to stir overnight at room temperature. After such time, the reaction was quenched with H_2O (25 mL) and extracted with DCM. The combined organic layers were dried over $MgSO_4$, filtered, and concentrated *in vacuo*. Purification by silica gel column chromatography (20% ethyl acetate in hexane) afforded the epoxide products.

2-(phenylsulfanylmethyl)oxirane (1L)

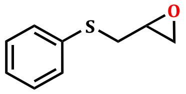

1L

Thiophenol (1.099 g, 9.98 mmol) was reacted according to **General Procedure 5** which afforded epoxide **1L** (480.6 mg, 29%) as a colorless oil. ^1H NMR (600 MHz, $CDCl_3$) δ 7.45 (d, J = 7.6 Hz, 1H), 7.33 (t, *J* = 7.7 Hz, 2H), 7.26 (t, *J* = 7.3 Hz, 1H), 3.26-3.13 (m, 2H), 2.97 (dd, *J* = 15.3, 7.3 Hz, 1H), 2.80 (t, *J* = 4.3 Hz, 1H), 2.55 (dd, *J* = 4.9, 2.4 Hz, 1H); ^{13}C NMR (151 MHz, $CDCl_3$) δ 135.3, 130.4, 129.1, 126.8, 51.1, 47.5, 36.7. The spectra are consistent with those reported in literature.[12]

1-(oxiran-2-ylmethyl)-1H-indole (1O)

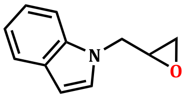

1O

Indole (1.196 g, 10.21 mmol) was reacted according to **General Procedure 5** which afforded epoxide **1O** (1.166 g, 66%) as a yellow liquid. ^1H NMR (600 MHz, $CDCl_3$) δ 7.67 (d, *J* = 7.9 Hz 1H), 7.42 (d, *J* = 8.3 Hz, 1H), 7.26 (t, *J* = 8.2 Hz, 1H), 7.17-7.14 (m, 2H), 6.56 (d, *J* = 3.2 Hz, 1H), 4.46 (d, *J* = 15.3 Hz, 1H), 4.23 (d, *J* = 15.3 Hz, 1H), 3.33-3.30 (m, 1H), 2.84 (d, *J* = 4.8 Hz, 1H), 2.49 (dd, *J* = 4.8, 2.6 Hz, 1H); ^{13}C NMR (151 MHz, $CDCl_3$) δ 136.3, 128.6, 128.2, 121.8, 121.0, 119.6, 109.3, 101.9, 50.9, 47.8, 45.3. The spectra are consistent with those reported in literature.[13]

Synthesis of 2-(oxiran-2-yl)-1-phenylethan-1-one (1M)

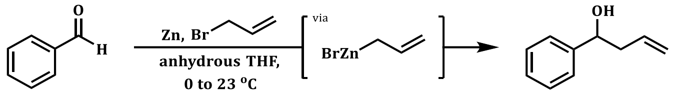

A 500-mL round bottom flask equipped with a magnetic stir bar was charged with Zn (3.062 g, 46.83 mmol, 3.0 equiv) and the flask was placed under vacuum and seal with a rubber septum. Anhydrous tetrahydrofuran (50 mL) was added and the mixture was brought to 0 ^{0}C. After stirring for 10 min at 0 ^{0}C, allyl bromide (6.352 g, 52. 50 mmol, 3.5 equiv) was slowly added and the solution was allowed to stir at room temperature for 1 hr. Benzaldehyde (**S3**, 1.591 g, 14.99 mmol, 1.0 equiv) was then added to solution via syringe and the reaction was stirred overnight. The reaction was quenched with sat. NH_4Cl and extracted with ethyl acetate. The combined organic layers were wash with brine, dried over $MgSO_4$, filtered, and concentrated *in vacuo*. The resulting residue was used without further purification.

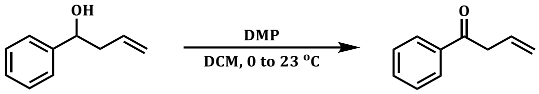

To a new 500-mL round bottom flask equipped with a magnetic stir bar, the crude allyl alcohol product (**S4**) was dissolved in dichloromethane (50 mL) and the solution was brought to 0 ^{0}C. Dess-Martin periodinane (DMP, 6.360 g, 14.99 mmol, 1.0 equiv) was added and the solution was stirred for 10 min at room temperature. The reaction was quenched with sat. $NaHCO_3$, and extracted with dichloromethane. The combined organic layers were washed with brine, dried of $MgSO_4$, filtered, and concentrated to afford the crude product. 1-phenylbut-3-en-1-one (**S5**) was isolated using CombiFlash Auto-Column Chromatography (20% ethyl acetate in hexane) as pale yellow oil (1.193 g, 8.160 mmol). ^{1}H NMR (600 MHz, $CDCl_3$): δ 7.99-7.96 (m, 2H), 7.59-7.55 (m, 1H), 7.49-7.45 (m, 2H), 6.10 (ddt, *J* = 17.1, 10.3, 6.7 Hz, 1H), 5.26-5.20 (m, 2H), 3.77 (dt, *J* = 6.7, 1.4 Hz, 2H); ^{13}C NMR (151 MHz, $CDCl_3$): δ 198.0, 136.6, 133.2, 131.1, 128.6, 128.3, 118.8, 43.5. The spectra are consistent with those reported in literature.[14]

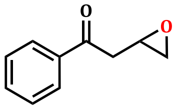

1M

1-phenylbut-3-en-1-one (**S5**, 1.193 g, 8.160 mmol) was reacted according to **General Procedure 2.** Purification by silica gel column chromatography (20% ethyl acetate in hexane) afforded epoxide **1M** (788.1 mg, 32.4% yield over 3 steps) as a yellow oil. ^{1}H NMR (600 MHz, CDCl3) δ 7.98 – 7.93 (m, 2H), 7.62 – 7.56 (m, 1H), 7.51 – 7.45 (m, 2H), 3.47 (tdd, J = 5.6, 4.0, 2.7 Hz, 1H), 3.36 (dd, J = 17.0, 5.7 Hz, 1H), 3.10 (dd, J = 17.0, 5.4 Hz, 1H), 2.93 (ddd, J = 4.7, 4.0, 0.6 Hz, 1H), 2.60 (dd, J = 4.9, 2.6 Hz, 1H), ^{13}C NMR (151 MHz, CDCl3) δ 197.4, 136.6, 133.5, 128.7, 128.2, 48.3, 47.0, 42.1; MS (ESI) calculated for $C_{10}H_{10}O_2Na$ $[M+Na]^+$: 185.0573, observed 185.0571.

Synthesis of N-methyl-N-glycidyl-aniline (1N)

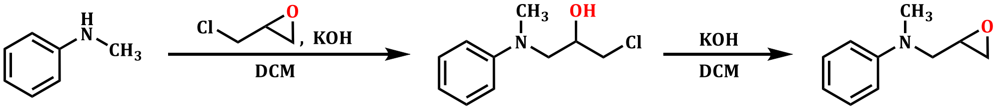

To a 500-mL round bottom flask equipped with a magnetic stir bar was added N-methylaniline (**S6**, 2.21 mL, 2.0 mmol, 1.0 equiv) and DCM (50 mL). A solution of KOH (2.24 g, 40.0 mmol, 20.0 equiv) in H_2O (20 mL) was added to the mixture. Epichlorohydrin (2.00 mL, 25.0 mmol, 12.5 equiv) was also added. The reaction mixture was stirred at room temperature overnight. After such time, the reaction was quenched with H_2O (100 mL)

and extracted with DCM. The combined organic layers were dried with $MgSO_4$, filtered, and concentrated *in vacuo*. The crude product (**S7**) was used in the next step without further purification.

S7 (crude from previous step) was added to a 250-mL round bottom flask equipped with a magnetic stir bar and dissolved in DCM (50 mL) and stirred at ambient temperature with 10% aqueous KOH (10 mL) for 24 hr. The reaction was quenched with sat. NH_4Cl (50 mL) and extracted with DCM. The combined organic layers were dried over $MgSO_4$, filtered, and concentrated *in vacuo*. Purification by silica gel column chromatography (20% ethyl acetate in hexane) afforded epoxide **1N** as a clear oil (2.296 g, 71%). ^1H NMR (600 MHz, $CDCl_3$) δ 7.26-7.22 (m, 2H), 6.77-6.72 (m, 3H), 3.64 (dd, J = 15.7, 3.2 Hz, 1H), 3.40 (dd, J = 15.7, 4.9 Hz, 1H), 3.17-3.14 (m, 1H), 3.00 (s, 3H), 2.79 (t, J = 4.5 Hz, 1H), 2.57 (dd, J = 5.0, 2.7 Hz, 1H); ^{13}C NMR (151 MHz, $CDCl_3$) δ 149.5, 129.2, 116.9, 112.5, 54.3, 50.6, 45.2, 39.1. The spectra are consistent with those reported in literature.[15]

Synthesis of 2-benzyloxymethyloxetane (S11)

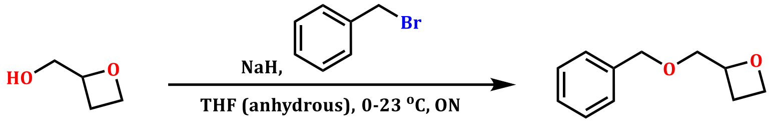

S10 S11

To a 25-mL two-necked round-bottom flask fitted with a magnetic stir bar was added NaH (60% dispersion in oil) (60.0 mg, 1.50 mmol, 1.5 equiv). One neck was plugged with a rubber septum and the flask was placed under nitrogen using Schlenk technique. 2-hydroxymethyloxetane (100.0 mg, 1.13 mmol, 1.0 equiv) was added to the flask, the mixture was dissolved in anhydrous THF (6 mL), and it was allowed to react at 0 °C for 1 hr. Then, benzyl bromide (0.145 mL, 1.22 mmol, 1.2 equiv) was added to the solution via syringe and the reaction was stirred overnight as it warmed to room temperature. The reaction mixture was diluted with Et_2O and quenched with the dropwise addition of sat. NH_4Cl. The organic layer was removed and the aqueous layer was extracted with Et_2O (2 x 25 mL). The combined organic layers were dried over Na_2SO_4, filtered, and concentrated. Purification by silica gel column chromatography (20% ethyl acetate in hexanes) afforded **S11** (64.7 mg, 41%) as a pale yellow liquid. ^1H NMR (600 MHz, $CDCl_3$): δ 7.40-7.27 (m, 5H), 5.00-4.95 (m, 1H), 4.70-4.65 (m, 2H), 4.63-4.56 (m, 2H), 3.68-3.60 (m, 2H), 2.69-2.62 (m, 1H), 2.61-2.54 (m, 1H); ^{13}C NMR (151 MHz, $CDCl_3$): δ 138.3, 128.4, 127.7, 127.6, 81.2, 73.5, 73.4, 69.0, 23.8. The spectra are consistent with those reported in literature.[16]

Vitamin B12 Catalyzed Epoxide Ring Opening

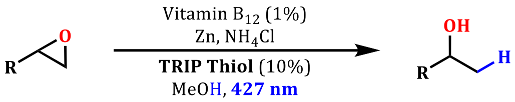

General Procedure 6

To an 8 mL septum screw-capped vial equipped with magnetic stir bar was added epoxide (**1A-Q**, 1.0 equiv), Vitamin B_{12} (cyanocobalamin, 1 mol %), Zn (3.0 equiv), and NH_4Cl (3.0 equiv). 2,4,6-triisopropylbenzene thiol ("TRIP thiol") was then added from a 0.1 mmol/mL stock solution in methanol (10 mol %) followed by the methanol solvent (0.1 mmol epoxide/mL methanol). This solution was sparged with a nitrogen balloon for 10 min and the vial was sealed with parafilm. The mixture was then stirred and irradiated with a 427 nm LED (Kessil®) for 2-48 hr. After reaction completion, the reaction mixture was concentrated *in vacuo*, suspended in dichloromethane, and sonicated before being passed through a cotton pipette filter. The filtrate was concentrated *in vacuo* and purified by silica gel column chromatography or preparative TLC to isolate the alcohol products featured below.

Reaction of Epoxide 1A

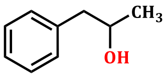

2A

Epoxide **1A** was reacted on 0.4 mmol scale for 48 hr. Purification by silica gel column chromatography (40% ethyl acetate in hexane) afforded alcohol **2A** (41.4 mg, 76% yield) as a pale yellow oil. ^1H NMR (600 MHz, $CDCl_3$): δ 7.36-7.33 (m, 2H), 7.28-7.23 (m, 3H), 4.09-4.02 (m, 1H), 2.82 (dd, *J* = 13.5, 4.8 Hz, 1H), 2.72 (dd, *J* = 13.5, 8.0 Hz, 1H), 1.59 (bs, 1H), 1.28 (d, *J* = 6.1 Hz, 3H); ^{13}C NMR (151 MHz, $CDCl_3$): δ 138.5, 129.4, 128.6, 126.5, 68.9, 45.8, 22.8. The spectra are consistent with those reported in literature.[8]

Reaction of Epoxide 1B

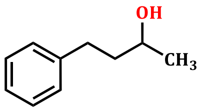

2B

Epoxide **1B** was reacted on 0.2 mmol scale for 48 hr. Purification by preparative TLC (40% ethyl acetate in hexane) afforded alcohol **2B** (17.3 mg, 58% yield) as a pale yellow liquid. ^1H NMR (600 MHz, $CDCl_3$): δ 7.31-7.26 (m, 2H), 7.23-7.16 (m, 3H), 3.86-3.79 (m, 1H), 2.79-2.72 (m, 1H), 2.71-2.64 (m, 1H), 1.83-1.72 (m, 2H), 1.42 (bs, 1H), 1.23 (d, *J* = 6.2 Hz, 3H); ^{13}C NMR (151 MHz, $CDCl_3$): δ 142.1, 128.4, 125.8, 67.5, 40.9, 32.1, 23.6. The spectra are consistent with those reported in literature.[17]

Reaction of Epoxide 1C

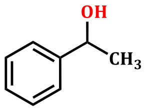

2C

Epoxide **1C** was reacted on 0.4 mmol scale for 47 hr. Purification by silica gel column chromatography (20% ethyl acetate in hexane) afforded alcohol **2C** (5.7 mg, 12% yield) as a colorless liquid. ^1H NMR (600 MHz, $CDCl_3$): δ 7.41-7.33 (m, 4H), 7.30-7.27 (m, 1H), 4.91 (q, *J* = 6.2 Hz, 1H), 1.81 (bs, 1H), 1.51 (d, *J* = 6.5 Hz, 3H); ^{13}C NMR (151 MHz, $CDCl_3$): δ 145.8, 128.5, 127.5, 125.4, 70.5, 25.2. The spectra are consistent with those reported in literature.[18]

Reaction of Epoxide 1D

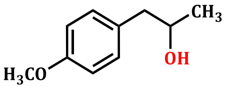

2D

Epoxide **1D** was reacted on 0.2 mmol scale for 16 hr. Purification by silica gel column chromatography (20% ethyl acetate in hexane) afforded alcohol **2D** (19.5 mg, 60% yield) as a yellow oil. ^{1}H NMR (600 MHz, $CDCl_3$) δ 7.15 (d, *J* = 8.6 Hz, 2H), 6.88 (d, J = 8.6 Hz, 2H), 4.05 – 3.91 (m, 1H), 3.82 (s, 3H), 2.76 (dd, *J* = 13.6, 4.8 Hz, 1H), 2.65 (dd, *J* = 13.6, 8.0 Hz, 1H), 1.25 (d, *J* = 6.2 Hz, 3H); ^{13}C NMR (151 MHz, $CDCl_3$) δ 158.3, 130.5, 130.4, 114.0, 114.0, 69.0, 55.3, 44.9, 22.7. The spectra are consistent with those reported in literature.[19]

Reaction of Epoxide 1E

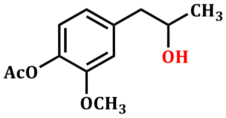

2E

Epoxide **1E** was reacted on 0.2 mmol scale for 2 hr. Purification by silica gel column chromatography (20% ethyl acetate in hexane) afforded alcohol **2E** (28.7 mg, 64% yield) as a yellow oil. ^{1}H NMR (600 MHz, $CDCl_3$) δ 6.96 (d, J = 8.0 Hz, 1H), 6.82 (d, J = 1.9 Hz, 1H), 6.78 (dd, J = 8.0, 1.9 Hz, 1H), 4.00 (dqd, J = 8.1, 6.2, 4.6 Hz, 1H), 3.81 (s, 3H), 2.76 (dd, J = 13.5, 4.6 Hz, 1H), 2.65 (dd, J = 13.5, 8.2 Hz, 1H), 2.30 (s, 3H), 1.25 (d, J = 6.2 Hz, 3H). ^{13}C NMR (151 MHz, $CDCl_3$) δ 169.3, 150.9, 138.3, 137.6, 122.7, 121.5, 113.5, 68.8, 55.8, 45.7, 22.8, 20.7. MS (ESI) calculated for $C_{12}H_{17}O_4$ $[M+H]^+$: 225.1121, observed 225.1107.

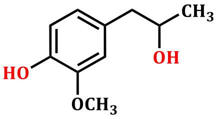

2E'

Epoxide **1E** was reacted on 0.2 mmol scale for 16 hr. Purification by silica gel column chromatography (30% ethyl acetate in hexane) afforded alcohol **2E'** (25.9 mg, 71% yield) as a yellow oil. ^{1}H NMR (600 MHz, $CDCl_3$) δ 6.85 (d, *J* = 7.9 Hz, 1H), 6.74 – 6.67 (m, 2H), 5.66 (bs, 1H), 3.98 (m, 1H), 3.87 (s, 3H), 2.73 (dd, *J* = 13.6, 4.6 Hz, 1H), 2.59 (dd, *J* = 13.6, 8.2 Hz, 1H), 1.24 (d, *J* = 6.2 Hz, 3H); ^{13}C NMR (151 MHz, $CDCl_3$) δ 146.6, 144.3, 130.3, 122.0, 114.5, 111.9, 69.0, 55.9, 45.4, 22.7. The spectra are consistent with those reported in literature.[20]

Reaction of Epoxide 1F

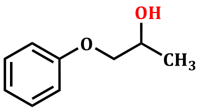

2F

Epoxide **1F** was reacted on 0.2 mmol scale for 48 hr. Purification by preparative TLC (40% ethyl acetate in hexanes) afforded alcohol **2F** (17.8 mg, 59% yield) as a yellowish oil. ^{1}H NMR (600 MHz, $CDCl_3$): δ 7.31-7.27 (m, 2H), 6.99-6.95 (m, 1H), 6.93-6.89 (m, 2H), 4.23-4.16 (m, 1H), 3.94 (dd, *J* = 9.2, 3.0 Hz, 1H), 3.79 (dd, *J* = 9.2, 8.0 Hz, 1H), 2.41 (bs, 1H), 1.28 (d, *J* = 6.4 Hz, 3H); ^{13}C NMR (151 MHz, $CDCl_3$): δ 158.5, 129.6, 121.1, 114.6, 73.2, 66.3, 18.8. The spectra are consistent with those reported in literature.[21]

Reaction of Epoxide 1G

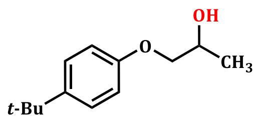

2G

Epoxide **1G** was reacted on 0.2 mmol scale for 48 hr. Purification by silica gel column chromatography (20% ethyl acetate in hexane) afforded alcohol **2G** (25.1 mg, 60% yield) as a yellow oil. ^{1}H NMR (600 MHz, $CDCl_3$): δ 7.33-7.29 (m, 2H), 6.87-6.83 (m, 2H), 4.22-4.15 (m, 1H), 3.93 (dd, *J* = 9.3, 3.2 Hz, 1H), 3.77 (dd, *J* = 9.2, 7.9 Hz, 1H), 2.42 (bs, 1H), 1.30 (s, 9H), 1.28 (d, *J* = 6.4 Hz, 3H); ^{13}C NMR (151 MHz, $CDCl_3$): δ 138.5, 129.4, 128.6, 126.5, 68.9, 45.8, 22.8. The spectra are consistent with those reported in literature.[8]

Reaction of Epoxide 1H

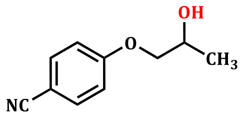

2H

Epoxide **1H** was reacted on 0.2 mmol scale for 48 hr. Purification by preparative TLC (40% ethyl acetate in hexane) afforded alcohol **2H** (16.9 mg, 48% yield) as a white crystalline solid. ^{1}H NMR (600 MHz, $CDCl_3$): δ 7.62-7.57 (m, 2H), 7.00-6.95 (m, 2H), 4.26-4.20 (m, 1H), 3.98 (dd, *J* = 9.2, 3.3 Hz, 1H), 3.87 (dd, *J* = 9.2, 7.5 Hz, 1H), 2.30 (bs, 1H), 1.31 (d, *J* = 6.3 Hz, 3H); ^{13}C NMR (151 MHz, $CDCl_3$): δ 161.9, 134.1, 119.1, 115.3, 104.4, 73.5, 66.1, 18.9. The spectra are consistent with those reported in literature.[8]

Reaction of Epoxide 1I

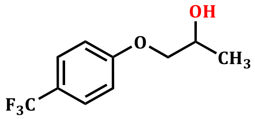

2I

Epoxide **1I** was reacted on 0.2 mmol scale for 44 hr. Purification by preparative TLC (40% ethyl acetate in hexane) afforded alcohol **2I** (29.4 mg, 66% yield) as a yellow oil. ^{1}H NMR (600 MHz, $CDCl_3$): δ 7.55 (d, *J* = 8.7 Hz, 2H), 6.98 (d, *J* = 8.7 Hz, 2H), 4.26-4.20 (m, 1H), 3.98 (dd, *J* = 9.2, 3.2 Hz, 1H), 3.85 (dd, *J* = 9.1, 7.7 Hz, 1H), 2.29 (bs, 1H), 1.31 (d, *J* = 6.4 Hz, 3H); ^{13}C NMR (151 MHz, $CDCl_3$): δ 161.0, 127.0 (q, J_{C-F} = 3.6 Hz), 124.4 (q, J_{C-F} = 271.3 Hz), 123.3 (q, J_{C-F} = 32.9 Hz), 114.5, 73.4, 66.2, 18.8; ^{19}F NMR (594 MHz, $CDCl_3$): δ -61.5. The spectra are consistent with those reported in literature.[8]

Reaction of Epoxide 1J

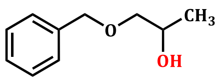

2J

Epoxide **1J** was reacted on 0.2 mmol scale for 24 hr. Purification by preparative TLC (40% ethyl acetate in hexane) afforded alcohol **2J** (20.8 mg, 63% yield) as a pale yellow oil. ^{1}H NMR (600 MHz, $CDCl_3$): δ 7.39-7.27 (m, 5H), 4.56 (s, 2H), 4.04-3.97 (m, 1H), 3.48 (dd, *J* = 9.4, 3.1 Hz, 1H), 3.29 (dd, *J* = 9.4, 8.2 Hz, 1H), 2.40 (bs, 1H), 1.15 (d, *J* = 6.4 Hz, 3H); ^{13}C NMR (151 MHz, $CDCl_3$): δ 138.0, 128.5, 127.8, 127.7, 75.8, 73.3, 66.5, 18.6. The spectra are consistent with those reported in literature.[8]

Reaction of Epoxide 1K

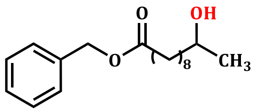

2K

Epoxide **1K** was reacted on 0.2 mmol scale for 16 hr. Purification by silica gel column chromatography (10% ethyl acetate in hexane) afforded alcohol **2K** (38.3 mg, 60% yield) as a transparent oil. ^{1}H NMR (600 MHz, $CDCl_3$) δ 7.35 (m, 5H), 5.11 (s, 2H), 3.79 (s, 1H), 2.35 (t, *J* = 7.5 Hz, 2H), 1.64 (m, 2H), 1.42 (m, 4H), 1.30 – 1.24 (m, 10H), 1.18 (d, *J* = 6.1 Hz, 3H);^{13}C NMR (151 MHz, $CDCl_3$) δ 173.7, 136.1, 128.6, 128.2, 68.2, 66.1, 39.3, 34.3, 29.6, 29.4, 29.2, 29.1, 25.7, 24.9, 23.5; MS (ESI) calculated for $C_{18}H_{28}O_3Na$ $[M+Na]^+$: 315.1928, observed 315.1931.

Reaction of Epoxide 1L

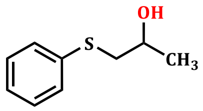

2L

Epoxide **1L** was reacted on 0.2 mmol scale for 18 hr. Purification by preparative TLC (20% ethyl acetate in hexane) afforded alcohol **2L** (23.1 mg, 67% yield) as a pale yellow oil. ^{1}H NMR (600 MHz, $CDCl_3$) δ 7.41 – 7.36 (m, 2H), 7.30 (t, *J* = 7.7 Hz, 2H), 7.25 – 7.19 (m, 1H), 3.85 (dqd, *J* = 9.8, 6.2, 3.6 Hz, 1H), 3.12 (dd, *J* = 13.8, 3.6 Hz, 1H), 2.84 (dd, *J* = 13.7, 8.6 Hz, 1H), 1.27 (d, *J* = 6.2 Hz, 2H);^{13}C NMR (151 MHz, $CDCl_3$) δ 135.1, 130.1, 129.1, 126.7, 65.5, 43.6, 21.9. The spectra are consistent with those reported in literature.[22]

Reaction of Epoxide 1M

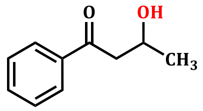

2M

Epoxide **1M** was reacted on 0.2 mmol scale for 48 hr. Purification by preparative TLC (20% ethyl acetate in hexane) afforded alcohol **2M** (14.4 mg, 38% yield) as a yellow oil. ^{1}H NMR (600 MHz, $CDCl_3$): δ 7.98-7.94 (m, 2H), 7.61-7.57 (m, 1H), 7.50-7.46 (m, 2H), 4.42 (m, 1H), 3.32 (bs, 1H), 3.19 (dd, *J* = 17.7, 2.7 Hz, 1H), 3.05 (dd, *J* = 17.7, 9.0 Hz, 1H), 1.31 (d, *J* = 6.4 Hz, 3H); ^{13}C NMR (151 MHz, $CDCl_3$): δ 200.9, 136.7, 133.6, 128.7, 128.1, 64.0, 46.5, 22.4. The spectra are consistent with those reported in literature.[23]

Reaction of Epoxide 1N

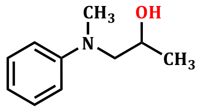

2N

Epoxide **1N** was reacted on 0.2 mmol scale for 16 hr. Purification by silica gel column chromatography (20% ethyl acetate in hexane) afforded alcohol **2N** (31.1 mg, 93% yield) as a yellow oil. ^{1}H NMR (600 MHz, $CDCl_3$) δ 7.33 – 7.09 (m, 2H), 6.96 – 6.79 (m, 2H), 6.76 (tt, *J* = 7.3, 1.1 Hz, 1H), 4.10 (q, *J* = 6.3 Hz, 1H), 3.22 (d, *J* = 6.3 Hz, 2H), 2.95 (s, 3H), 1.23 (d, *J* = 6.3 Hz, 3H); ^{13}C NMR (151 MHz, $CDCl_3$) δ 150.5, 129.2, 117.5, 113.4, 65.5, 61.82 39.3, 20.1. The spectra are consistent with those reported in literature.[24]

Reaction of Epoxide 1O

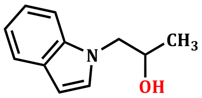

2O

Epoxide **1O** was reacted on 0.2 mmol scale for 16 hr. Purification by silica gel column chromatography (20% ethyl acetate in hexane) afforded alcohol **2O** (32.3 mg, 92% yield) as a yellow oil. ^{1}H NMR (600 MHz, $CDCl_3$) δ 7.65 (dt, J = 7.9, 1.0 Hz, 1H), 7.40 – 7.35 (m, 1H), 7.23 (m, 1H), 7.16 – 7.10 (m, 2H), 6.53 (dd, J = 3.1, 0.9 Hz, 1H), 4.23 – 4.13 (m, 2H), 4.06 – 3.99 (m, 1H), 1.74 (d, J = 3.7 Hz, 1H), 1.26 (d, J = 6.3 Hz, 3H); ^{13}C NMR (151 MHz, $CDCl_3$) δ 136.3, 128.6, 128.6, 121.7, 121.1, 119.6, 109.6, 101.6, 67.3, 53.9, 20.5. The spectra are consistent with those reported in literature.[25]

Reaction of Epoxide 1P

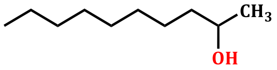

2P

Epoxide **1P** was reacted on 0.2 mmol scale for 24 hr. Purification by silica gel column chromatography (20% ethyl acetate in hexane) afforded alcohol **2P** (16.7 mg, 53% yield) as a colorless oil. ^{1}H NMR (600 MHz, $CDCl_3$): δ 3.82-3.77 (m, 1H), 1.49-1.36 (m, 2H), 1.34-1.24 (m, 12H), 1.19 (d, *J* = 6.1 Hz, 3H), 0.88 (t, *J* = 6.9 Hz, 3H); ^{13}C NMR (151 MHz, $CDCl_3$): δ 68.2, 39.4, 31.9, 29.7, 29.6, 29.3, 25.8, 23.5, 22.7, 14.1. The spectra are consistent with those reported in literature.[21]

Reaction of Epoxide 1Q

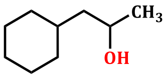

2Q

Epoxide **1Q** was reacted on 0.2 mmol scale for 16 hr. Purification by silica gel column chromatography (100% hexane) afforded alcohol **2Q** (27.8 mg, 97% yield) as a transparent oil. ^{1}H NMR (600 MHz, $CDCl_3$) δ 3.92 (s, 1H), 1.78 (d, *J* = 11.1 Hz, 1H), 1.68 (m, 3H), 1.38 (m, 3H), 1.25 (m, 3H), 1.18 (m, 4H), 0.91 (m, 2H); ^{13}C NMR (151 MHz, $CDCl_3$) δ 65.5, 47.3, 34.3, 34.0, 33.1, 26.6, 26.4, 26.2, 24.1. The spectra are consistent with those reported in literature.[26]

Unreactive Epoxides

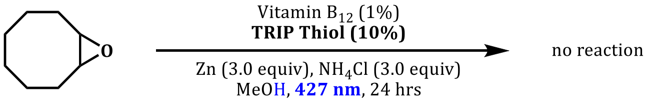

S8

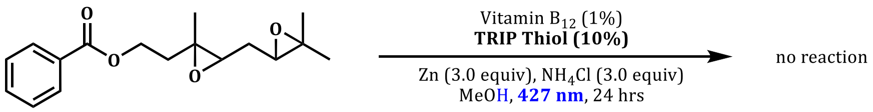

S9

Reaction of Oxetane S11

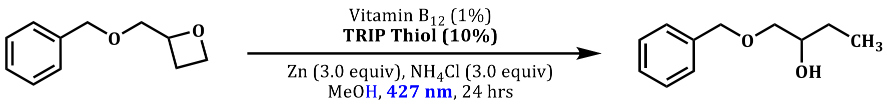

S12: 10% (NMR yield)

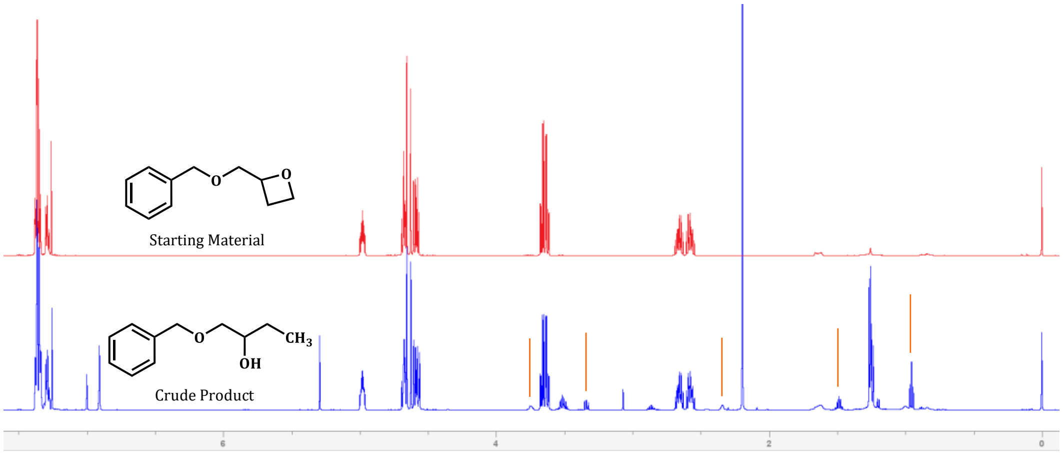

^1H NMR, ^{13}C NMR, and ^{19}F NMR Spectra

^1H NMR (600 MHz, $CDCl_3$) of 1,2-epoxy-4-phenylbutane (**1B**)

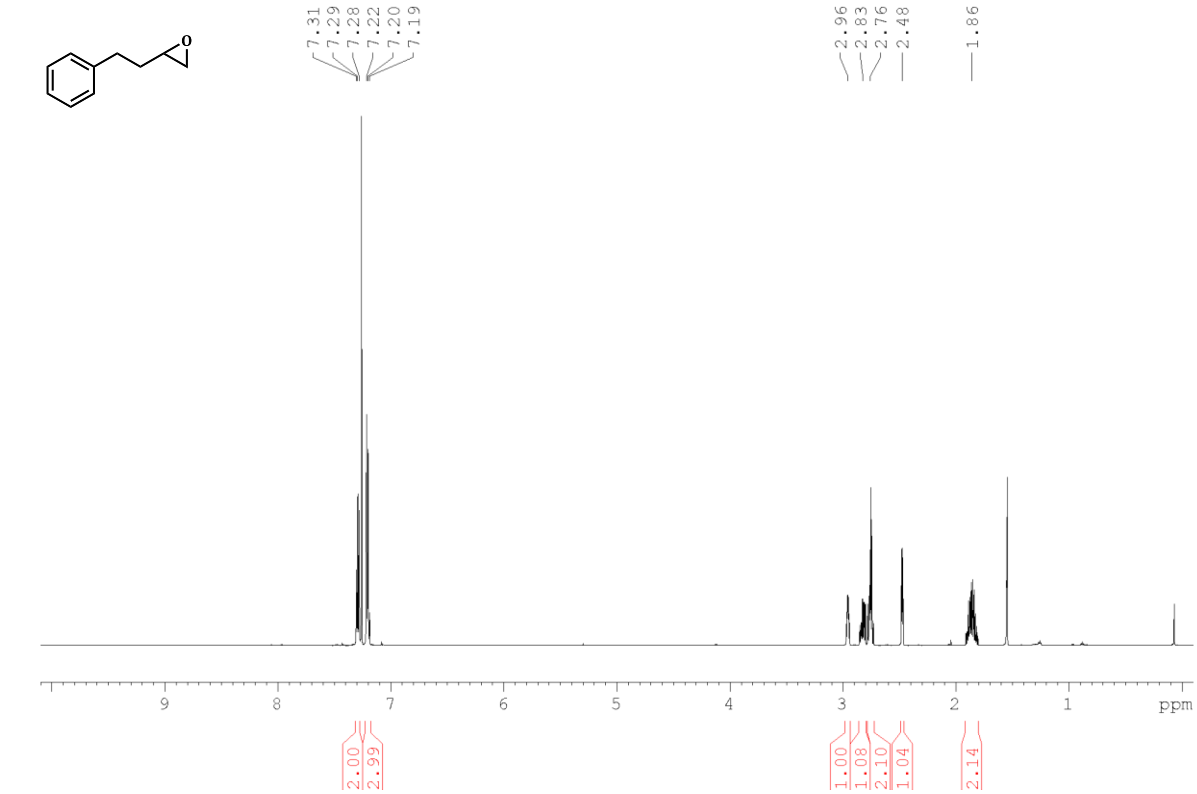

^{13}C NMR (151 MHz, $CDCl_3$) of 1,2-epoxy-4-phenylbutane (**1B**)

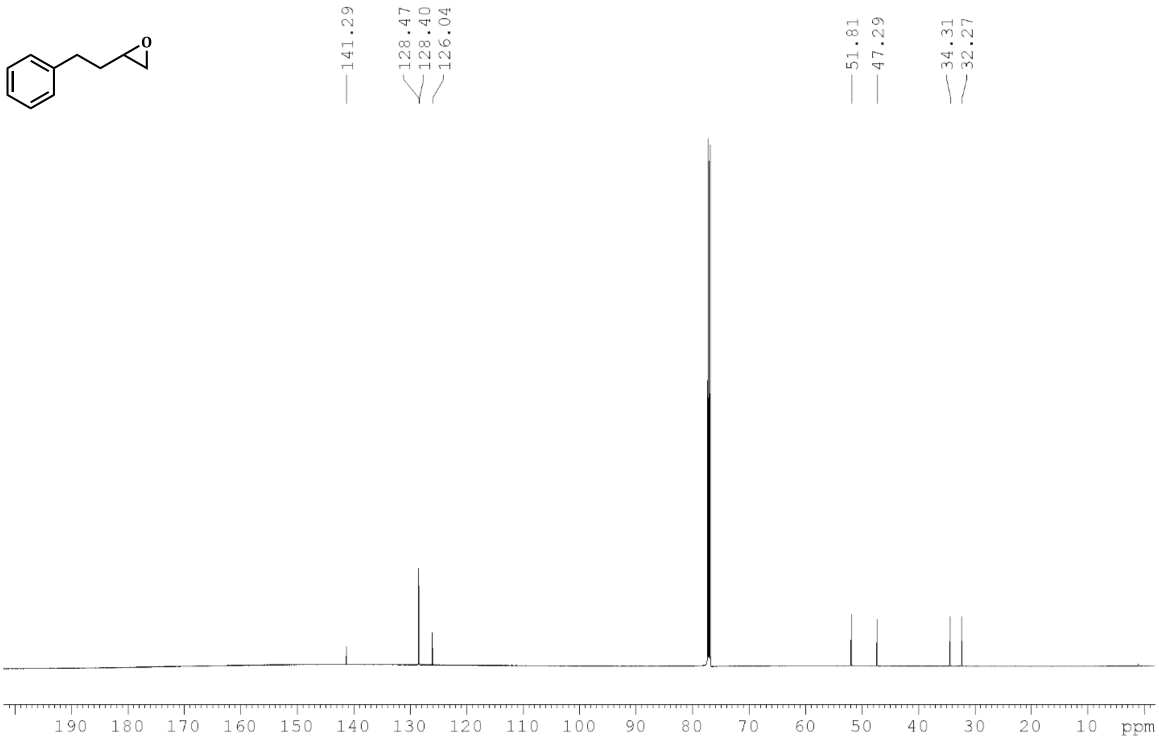

^{1}H NMR (600 MHz, $CDCl_3$) of estragole oxide (**1D**)

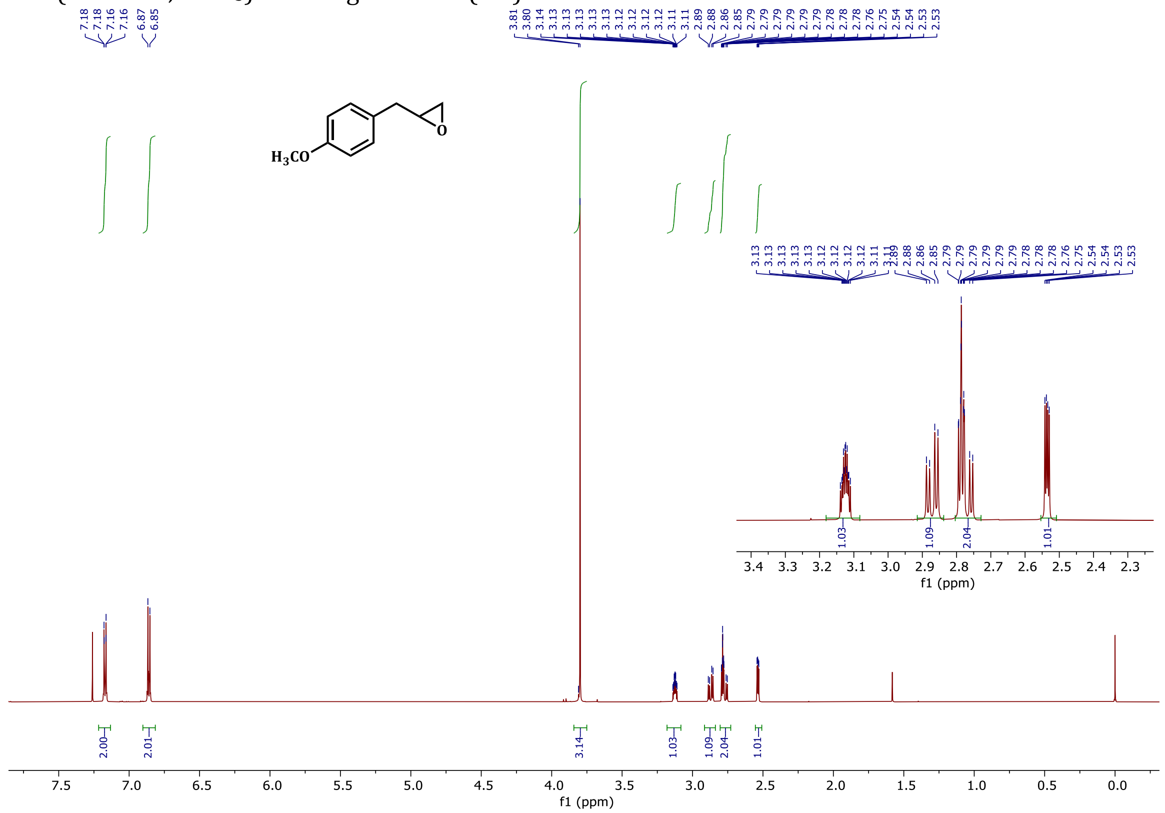

^{13}C NMR (151 MHz, $CDCl_3$) of estragole oxide (**1D**)

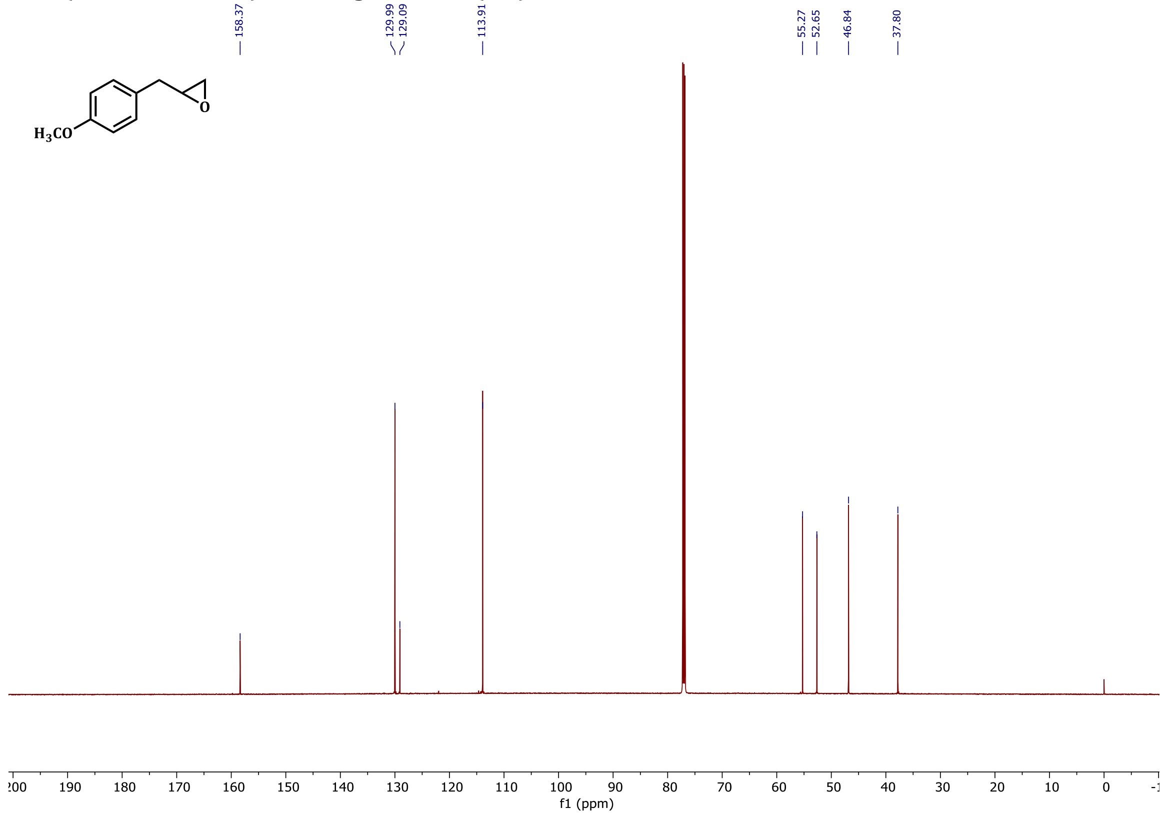

^{1}H NMR (600 MHz, $CDCl_3$) of *o*-acetyleugenol oxide (**1E**)

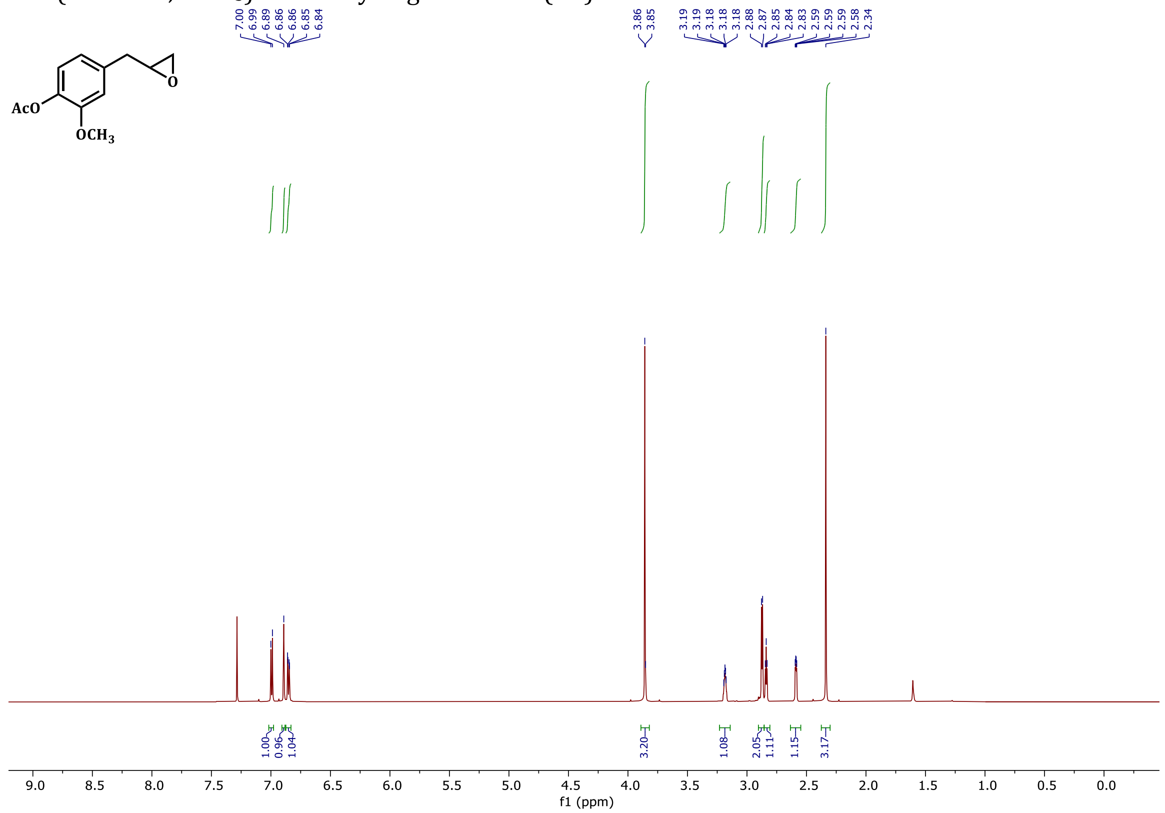

^{13}C NMR (151 MHz, $CDCl_3$) of *o*-acetyleugenol oxide (**1E**)

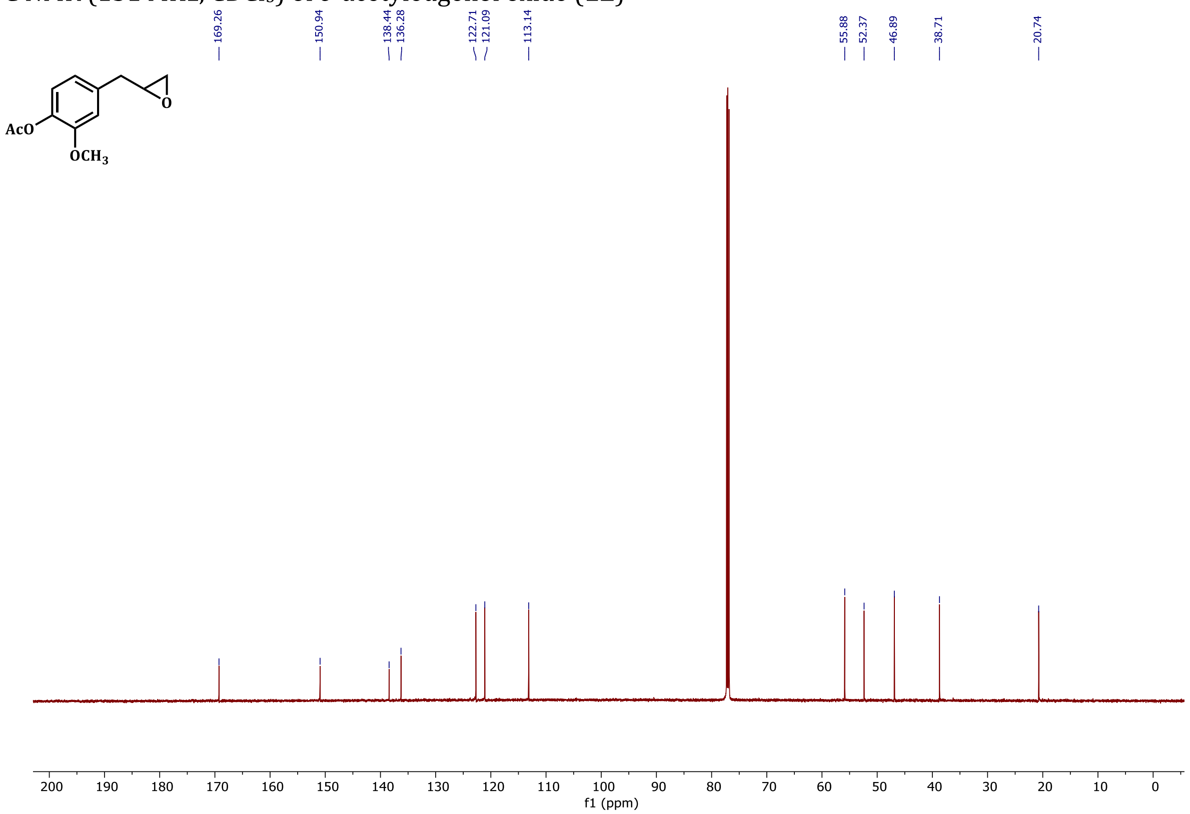

^{1}H NMR (600 MHz, $CDCl_3$) of 4-tert-butylphenyl 2,3-epoxypropyl ether (**1G**)

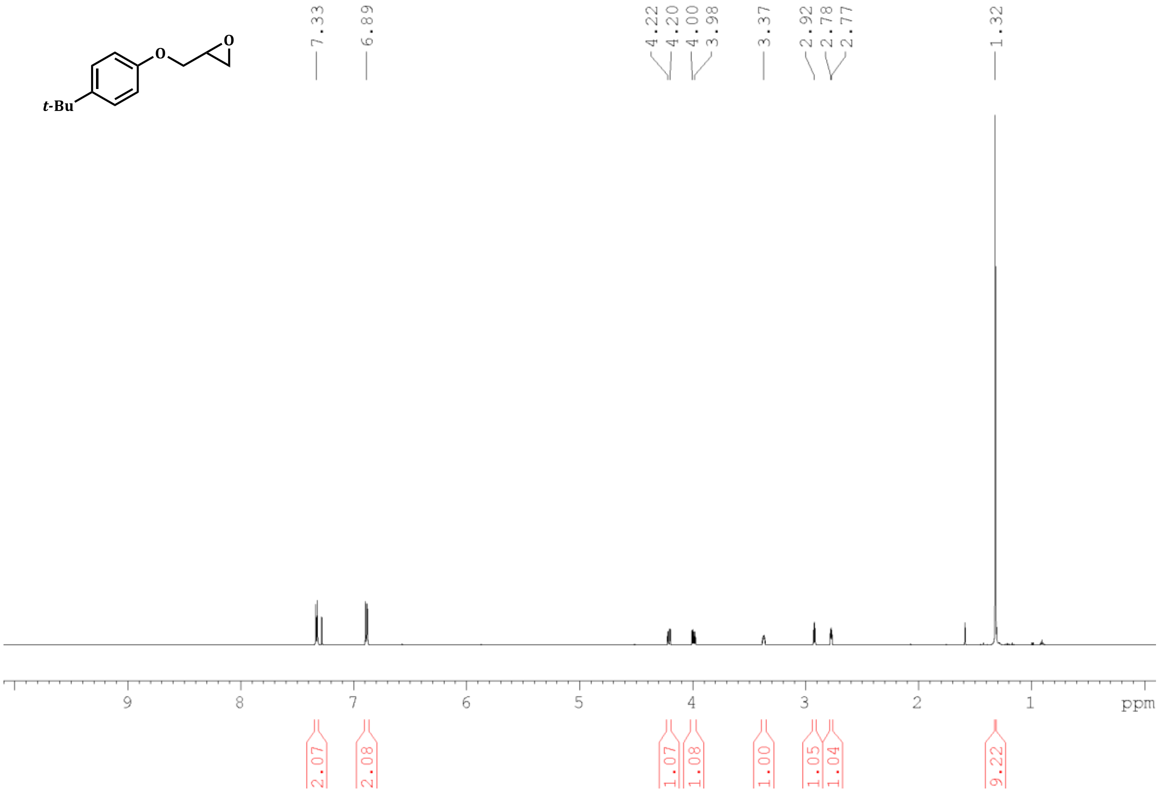

^{13}C NMR (151 MHz, $CDCl_3$) of 4-tert-butylphenyl 2,3-epoxypropyl ether (**1G**)

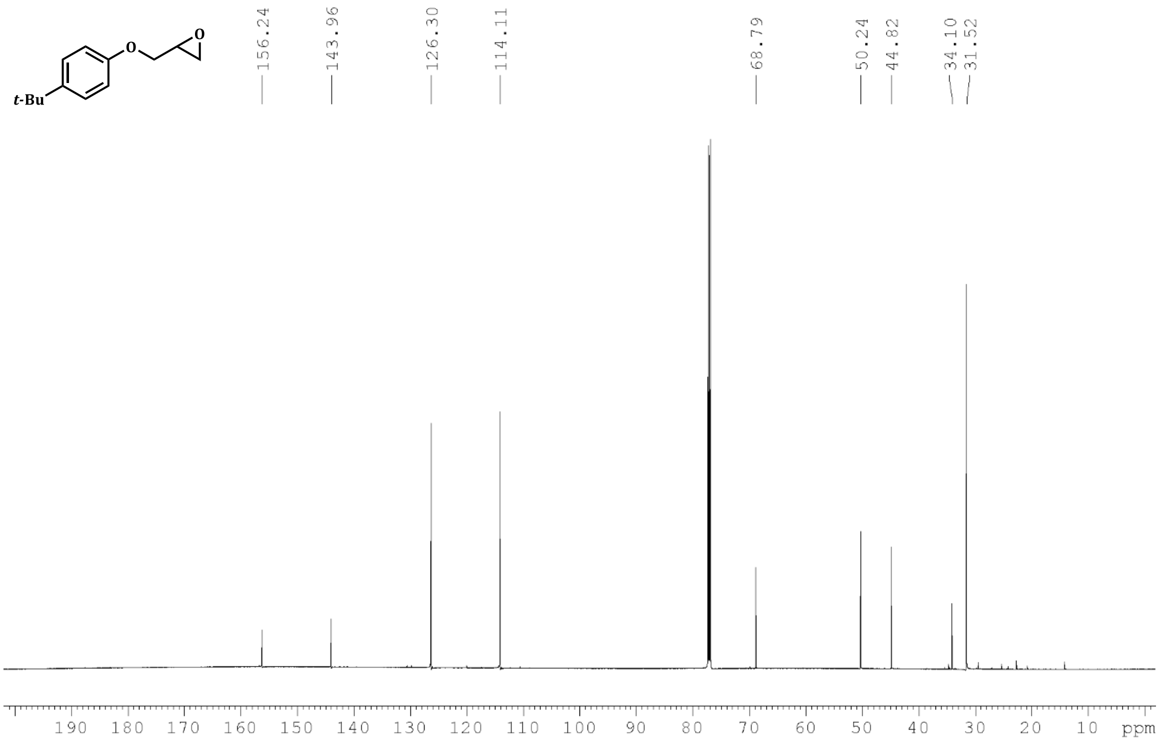

^{1}H NMR (600 MHz, $CDCl_3$) of 4-(2-oxiranylmethoxy) benzonitrile (**1H**)

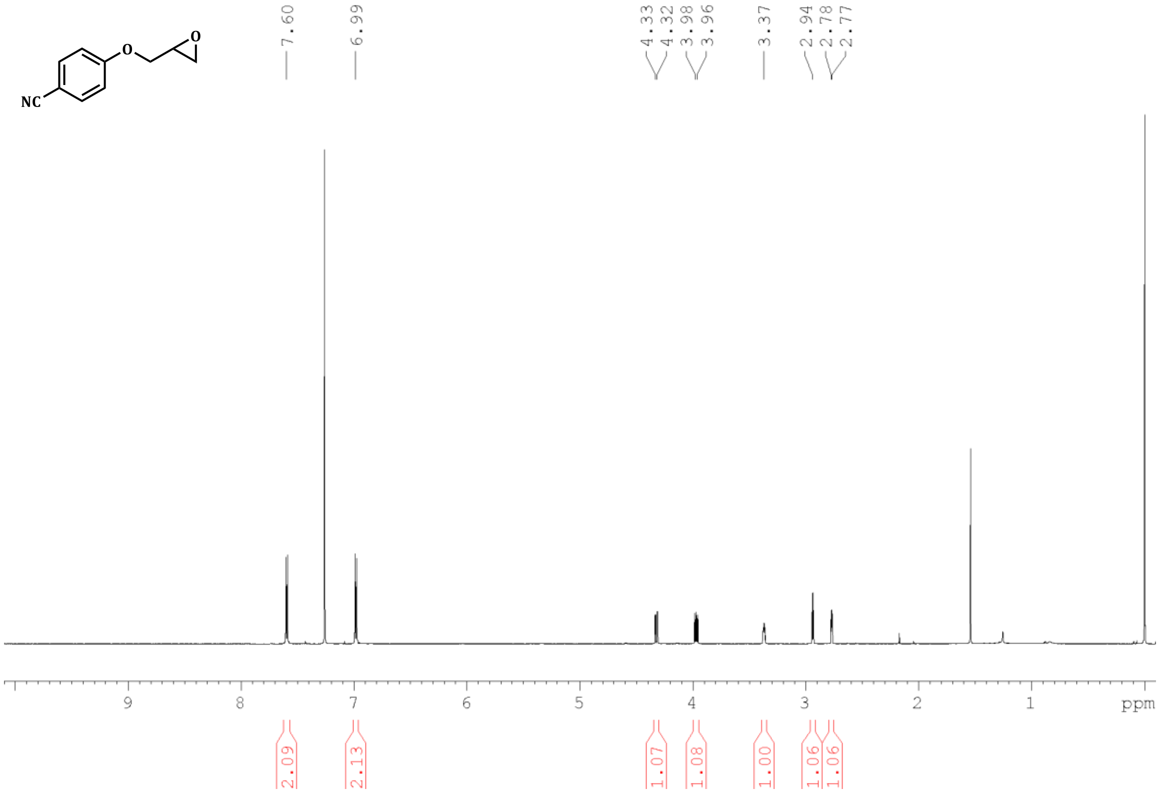

^{13}C NMR (151 MHz, $CDCl_3$) of 4-(2-oxiranylmethoxy) benzonitrile (**1H**)

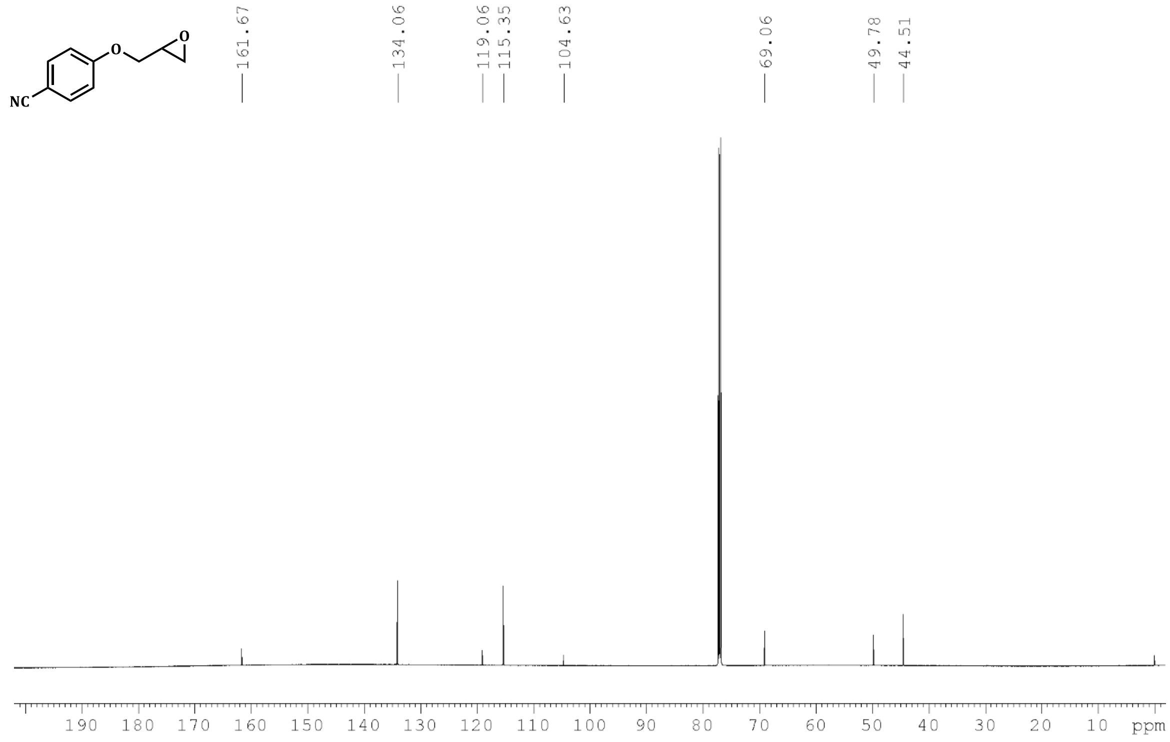

^1H NMR (600 MHz, $CDCl_3$) of 2-((4-(trifluoromethyl)phenoxy)methyl) oxirane (**1l**)

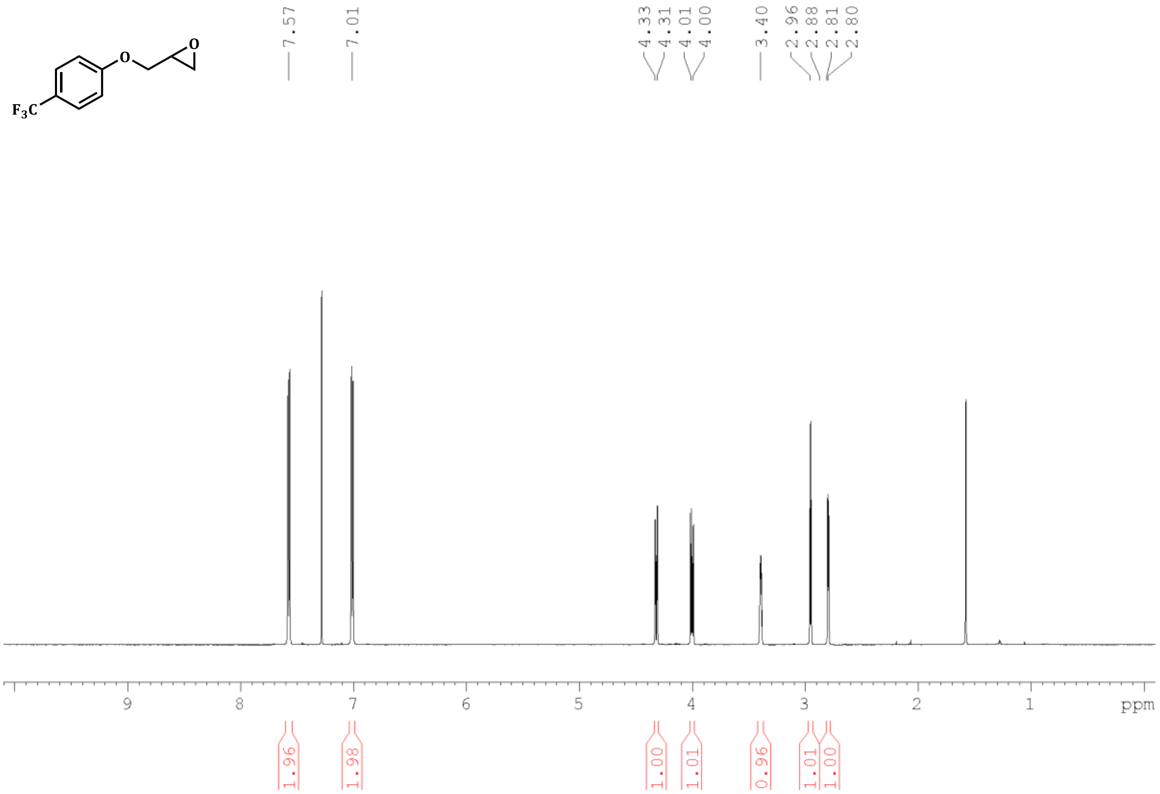

^{13}C NMR (151 MHz, $CDCl_3$) of 2-((4-(trifluoromethyl)phenoxy)methyl) oxirane (**1l**)

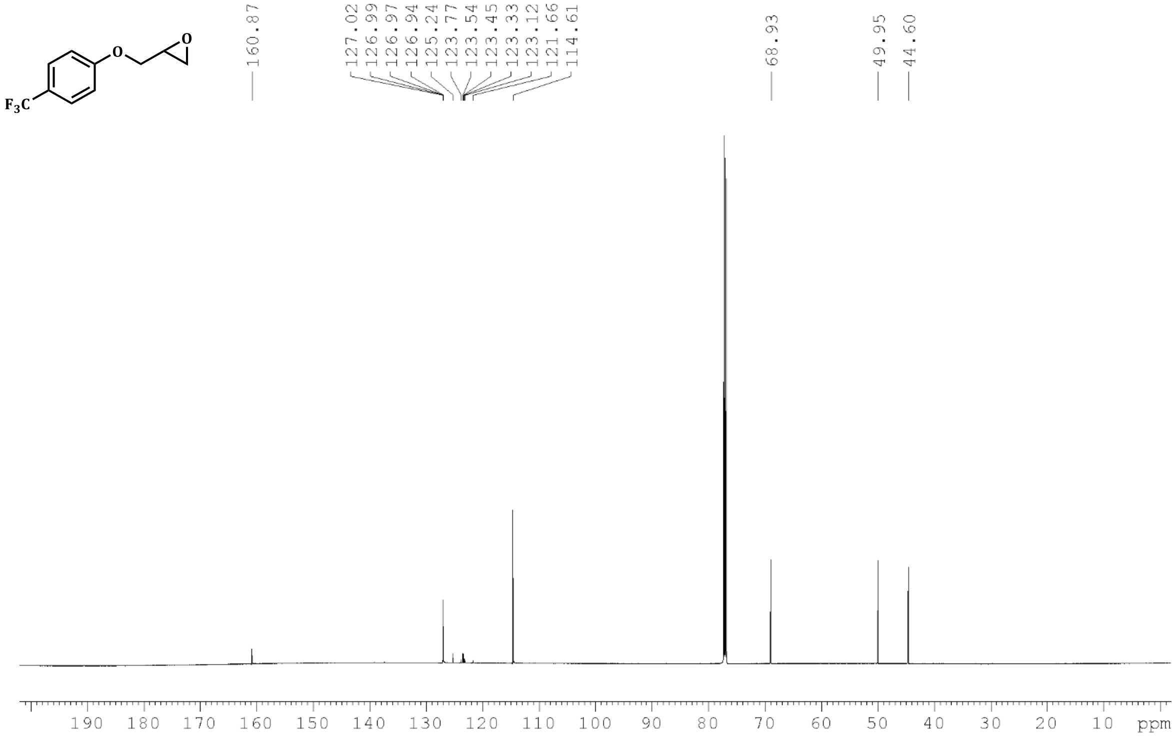

^{19}F NMR (594 MHz, $CDCl_3$) of 2-((4-(trifluoromethyl)phenoxy)methyl) oxirane (**1I**)

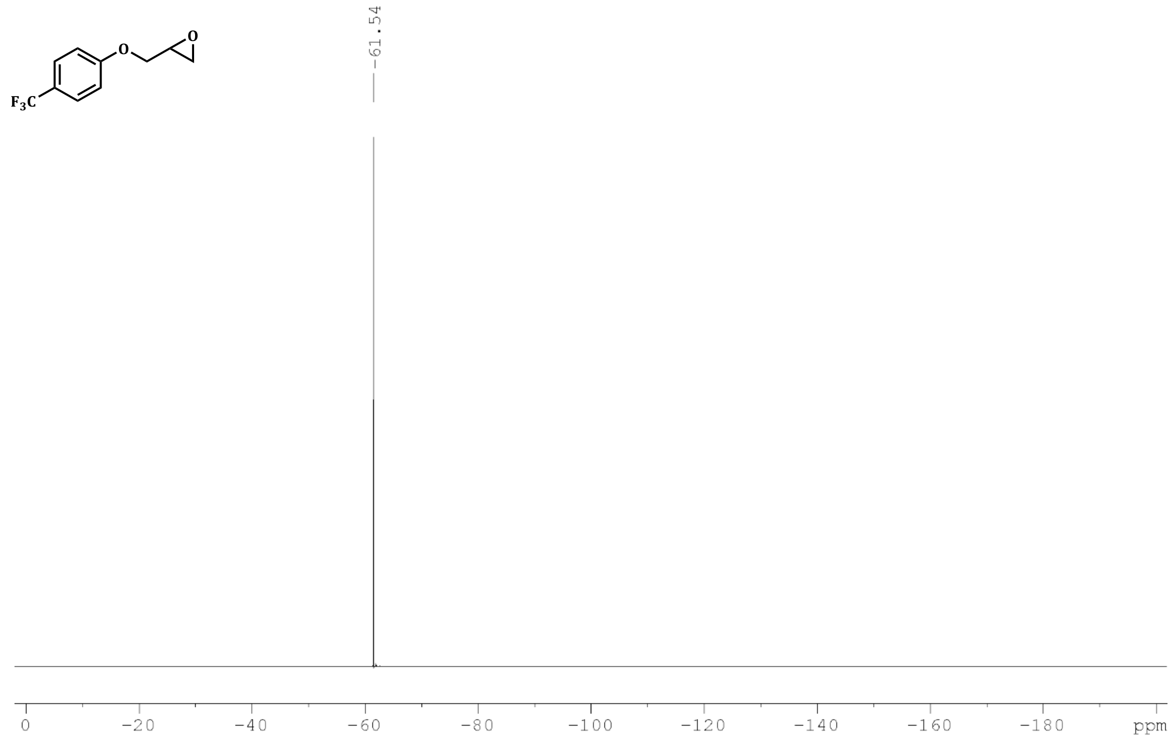

^{1}H NMR (600 MHz, $CDCl_3$) of benzyl-10,11-epoxy-undecanoate (**1K**)

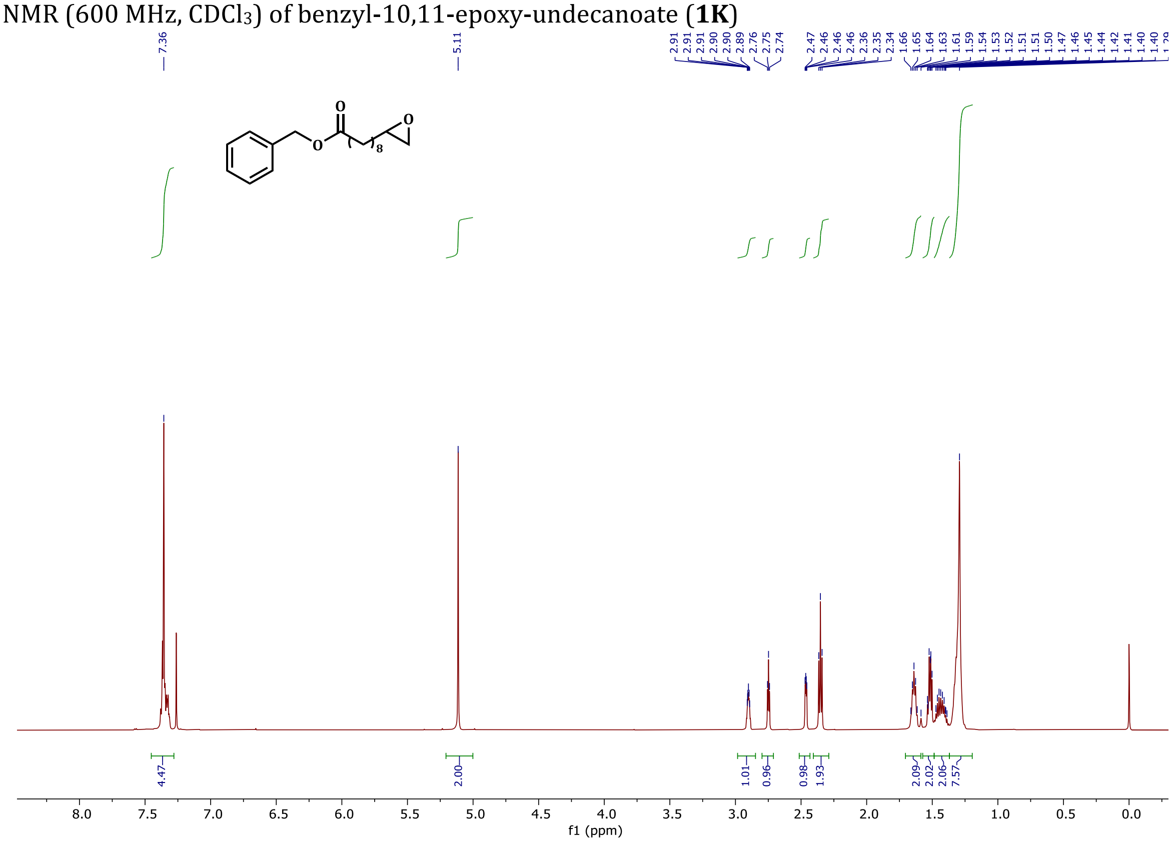

^{13}C NMR (151 MHz, $CDCl_3$) of benzyl-10,11-epoxy-undecanoate (**1K**)

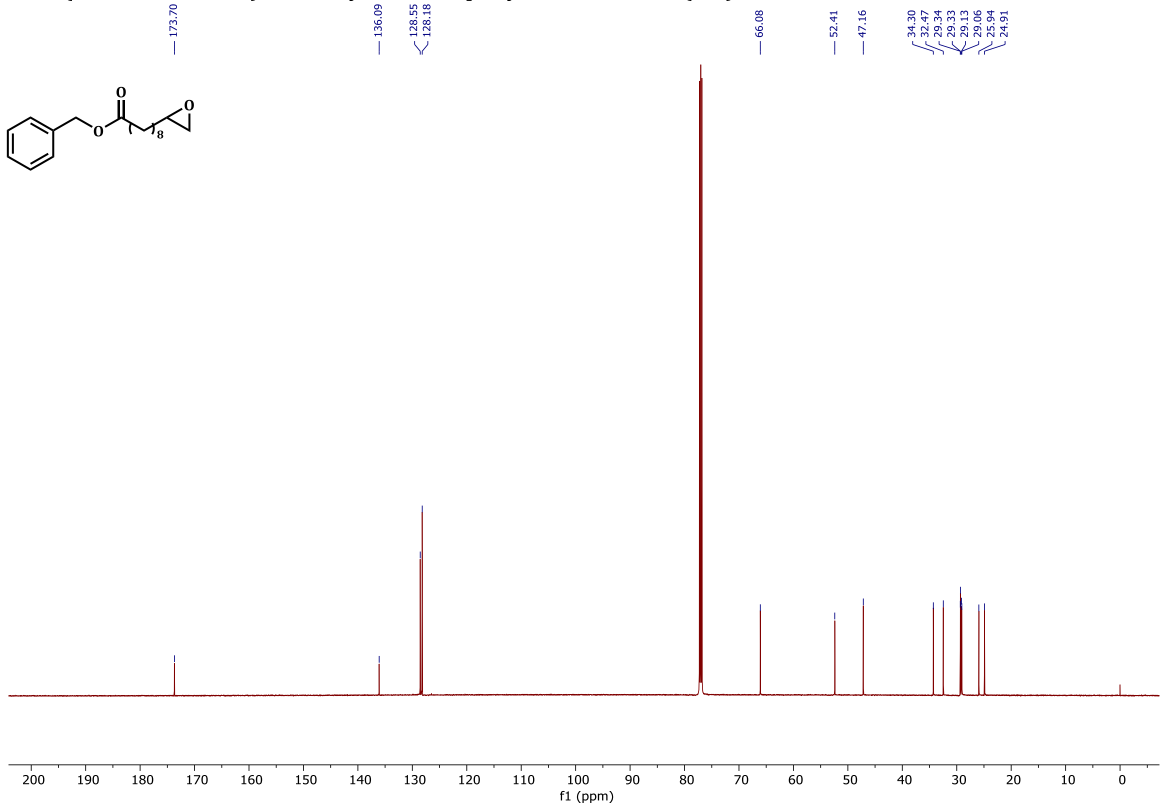

^{1}H NMR (600 MHz, $CDCl_3$) of 2-(phenylsulfanylmethyl)oxirane (**1L**)

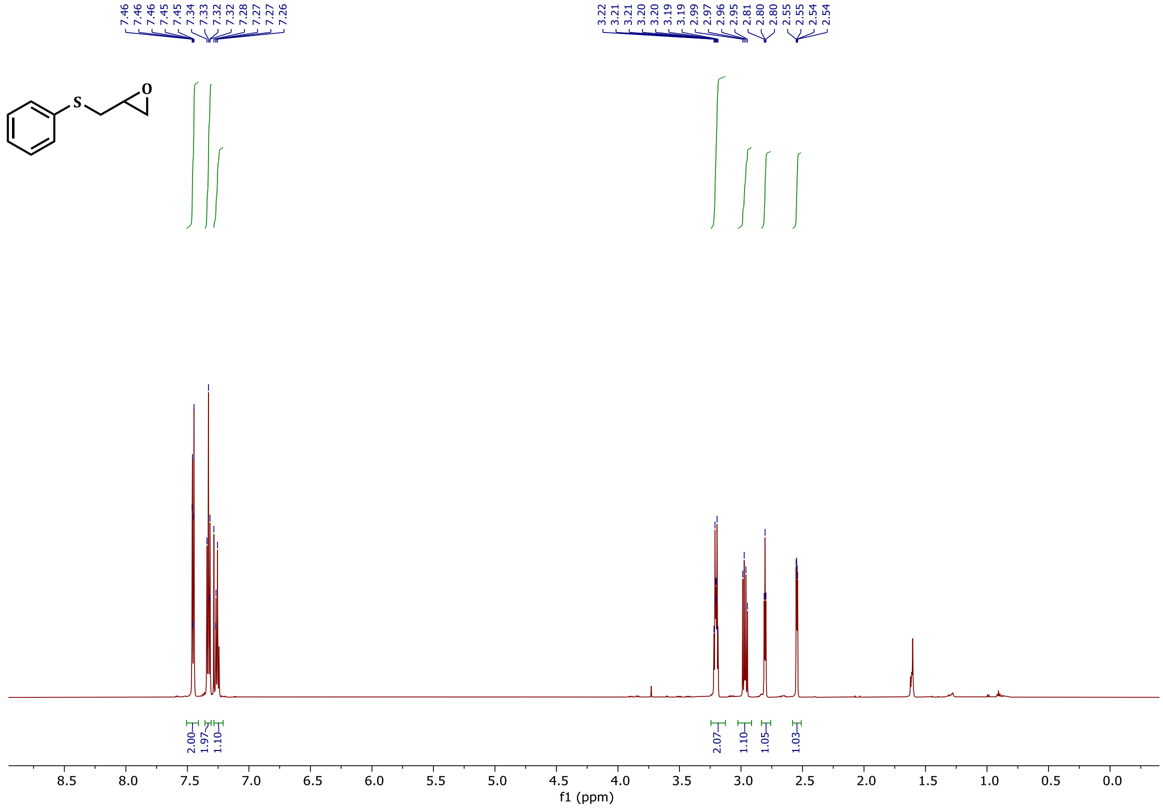

^{13}C NMR (151 MHz, $CDCl_3$) of 2-(phenylsulfanylmethyl)oxirane (**1L**)

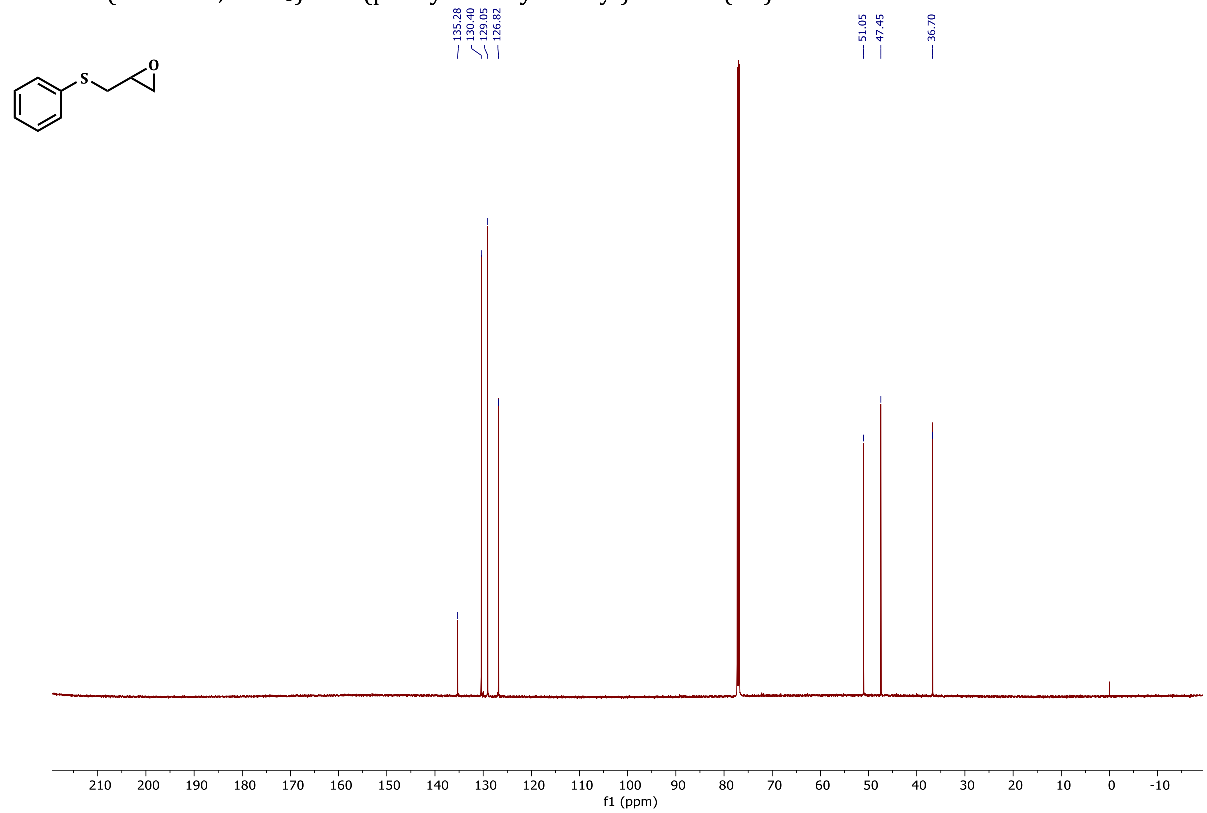

^{1}H NMR (600 MHz, $CDCl_3$) of 1-phenylbut-3-en-1-one (**S5**)

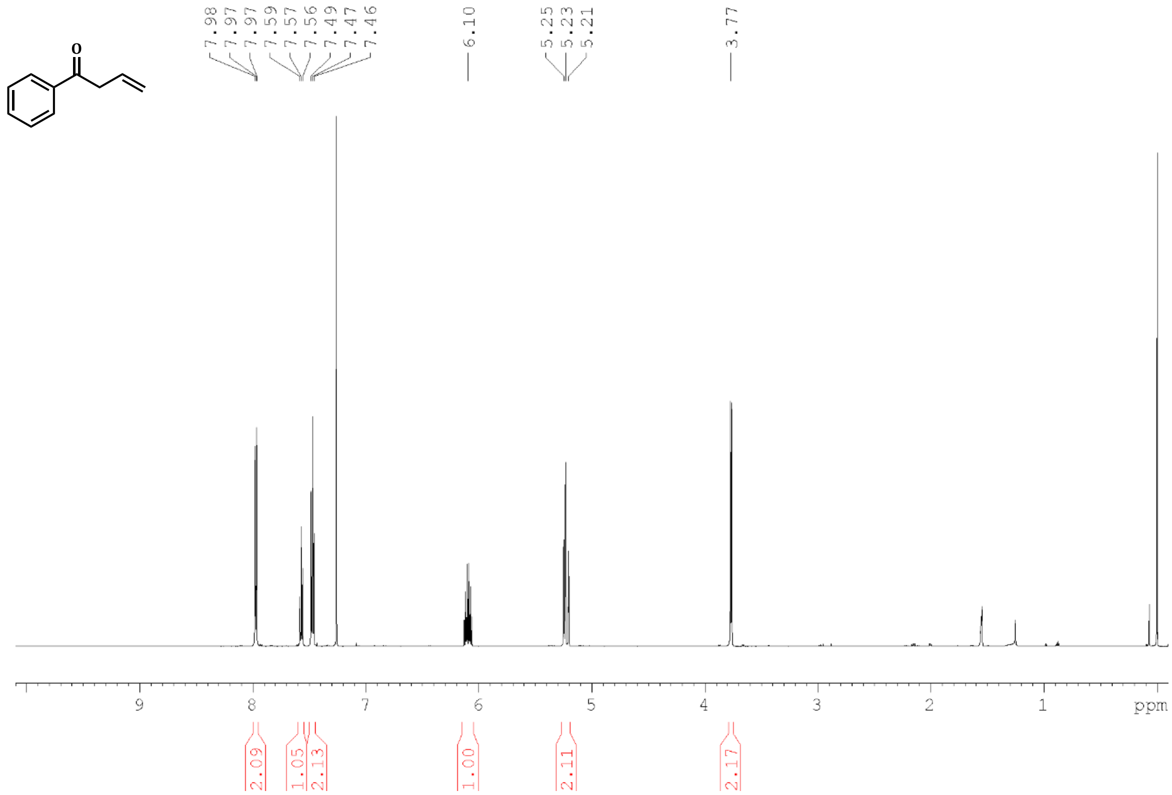

^{13}C NMR (151 MHz, $CDCl_3$) of 1-phenylbut-3-en-1-one (**S5**)

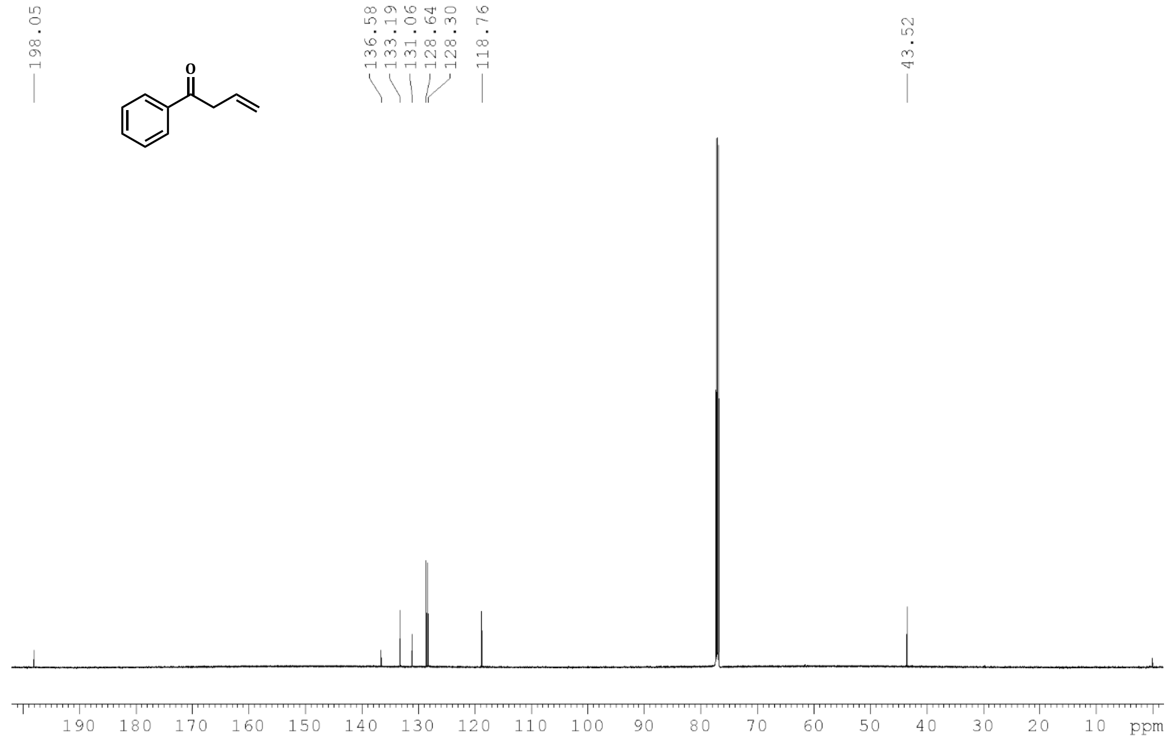

^{1}H NMR (600 MHz, $CDCl_3$) of 2-(oxiran-2-yl)-1-phenylethan-1-one (**1M**)

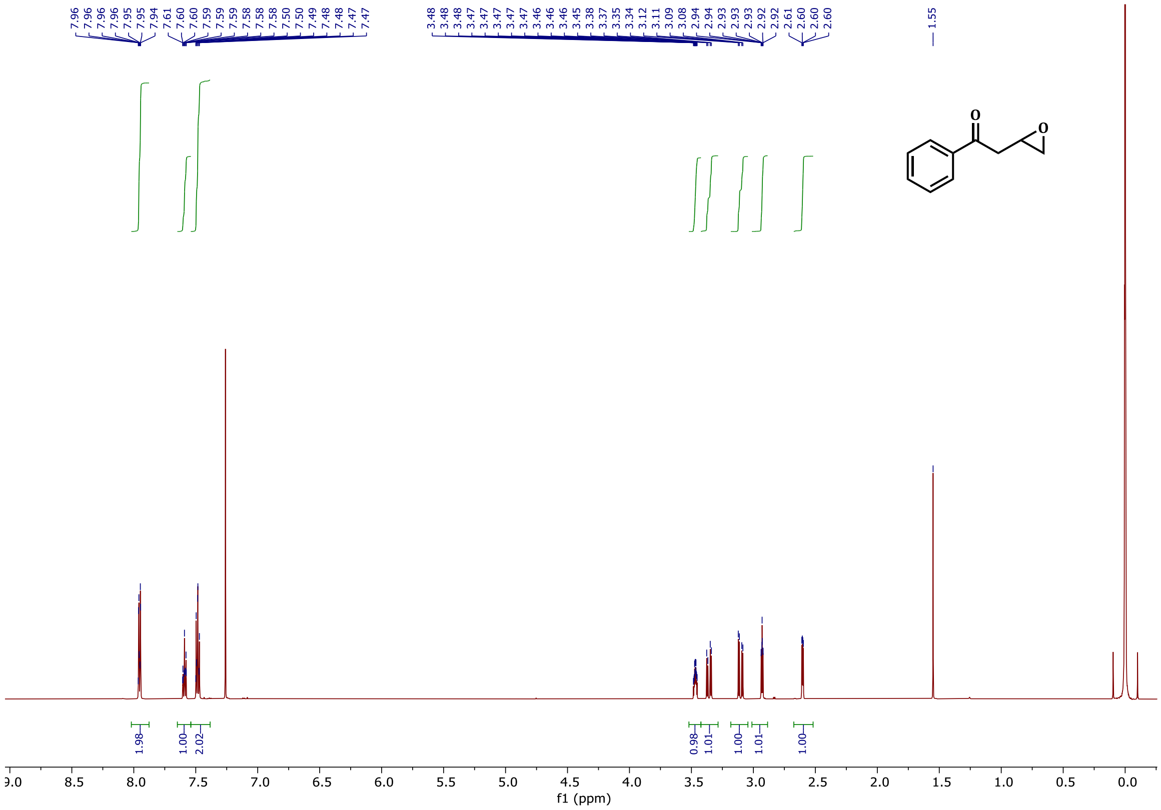

^{13}C NMR (151 MHz, $CDCl_3$) of 2-(oxiran-2-yl)-1-phenylethan-1-one (**1M**)

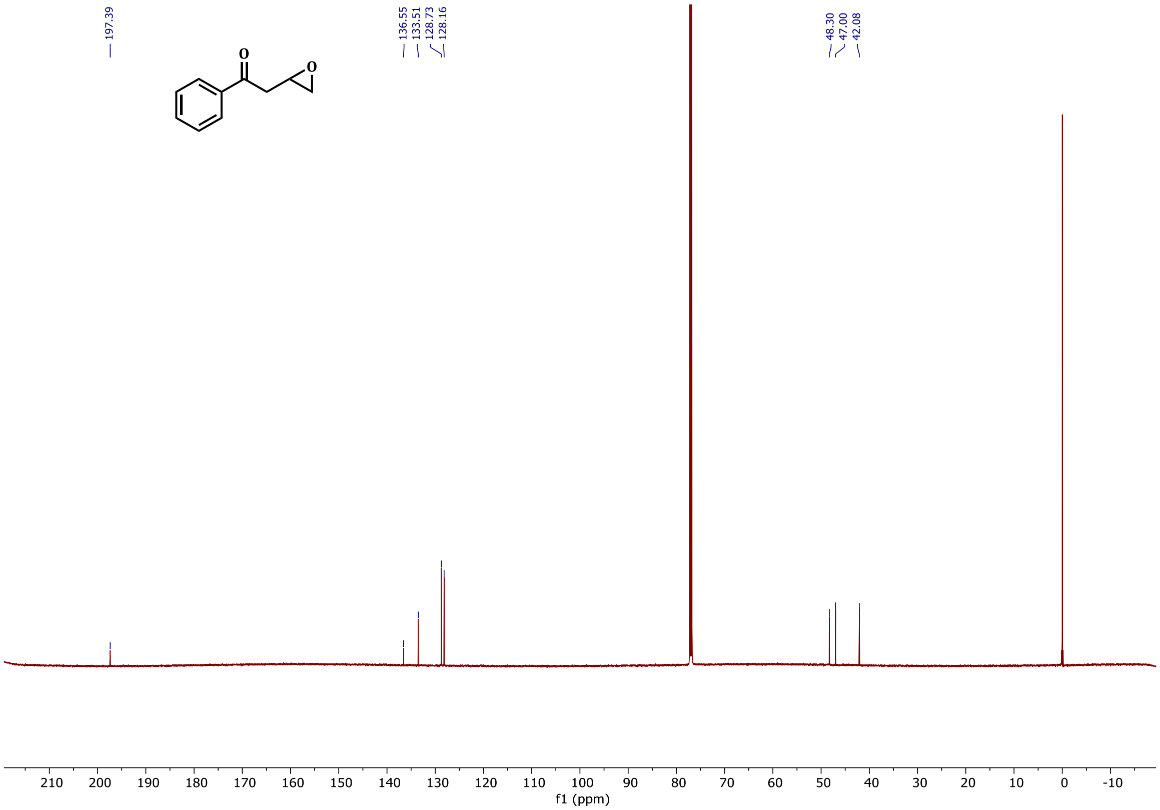

^{1}H NMR (600 MHz, $CDCl_3$) of N-methyl-N-glycidyl-aniline (**1N**)

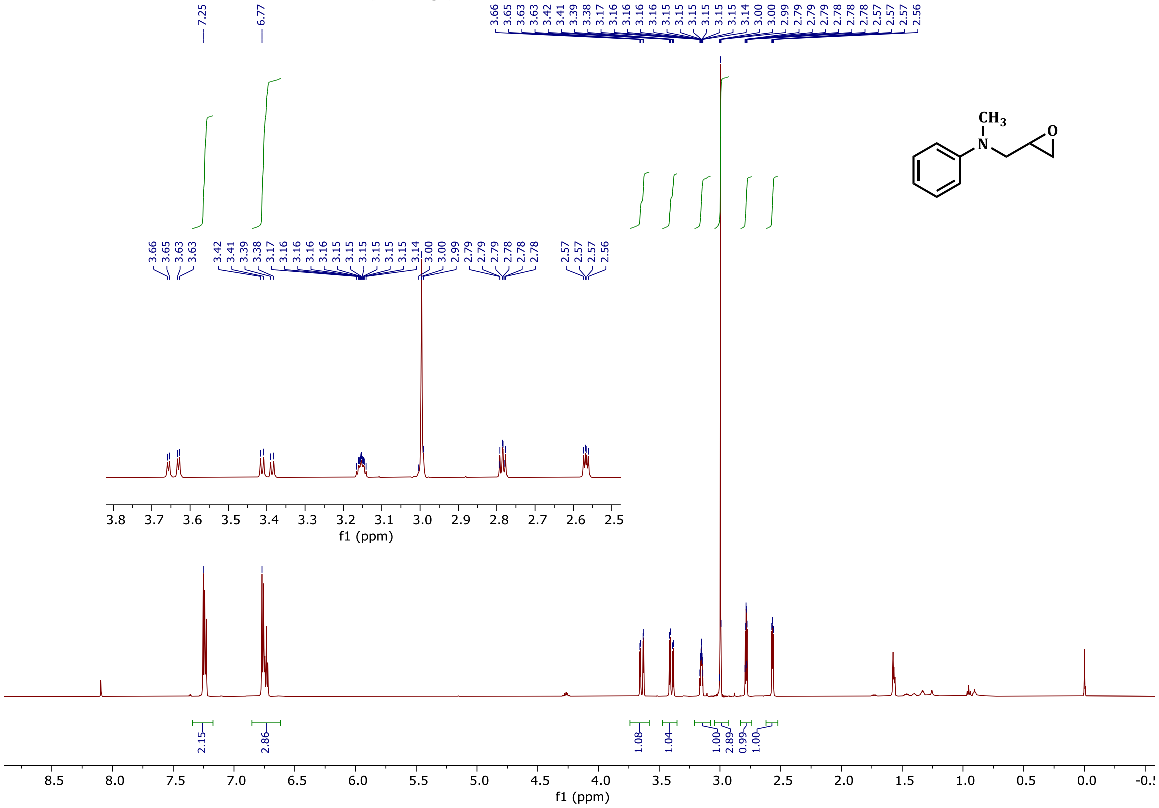

^{13}C NMR (151 MHz, $CDCl_3$) of N-methyl-N-glycidyl-aniline (**1N**)

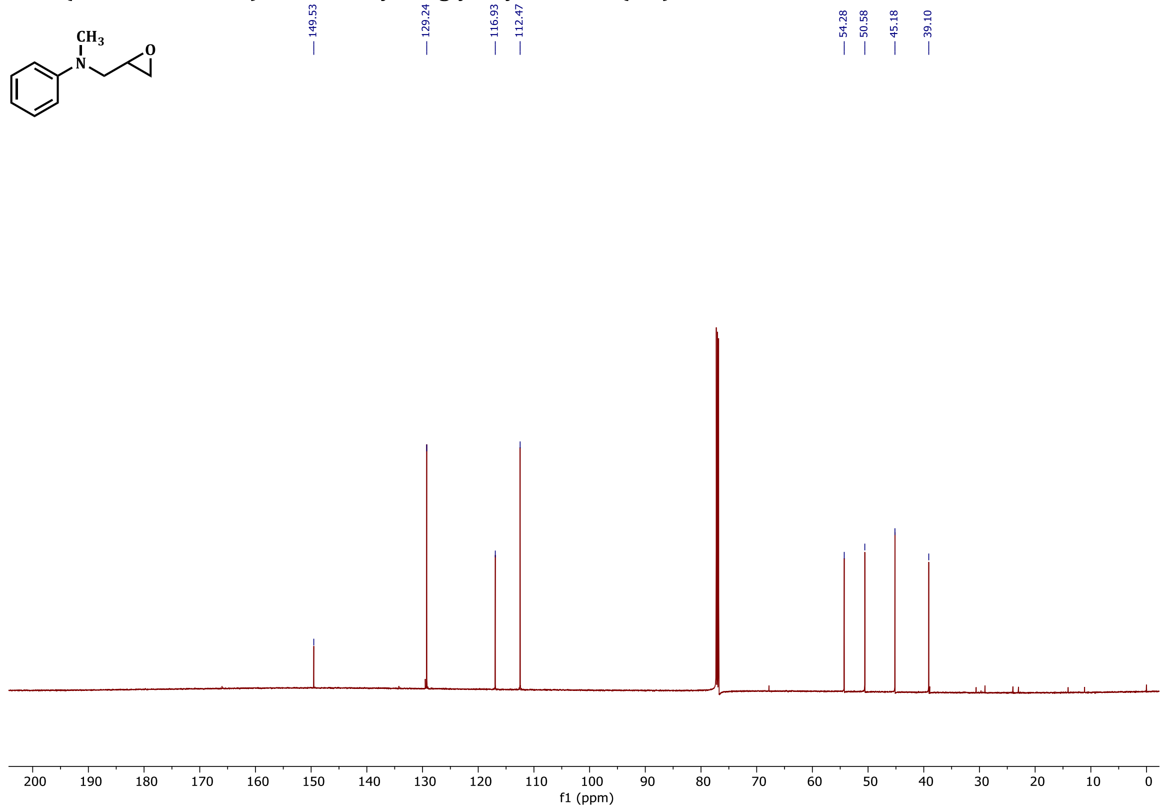

^{1}H NMR (600 MHz, $CDCl_3$) of 1-(oxiran-2-ylmethyl)-1H-indole (**1O**)

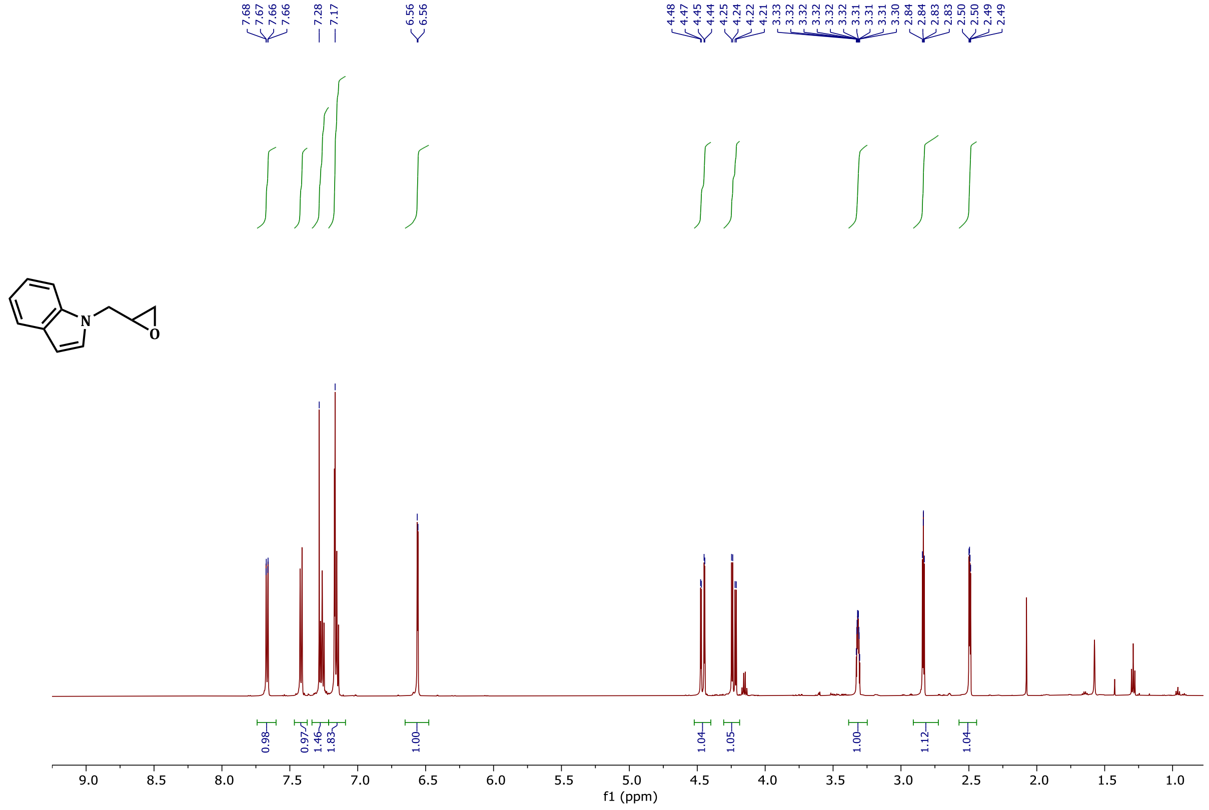

^{13}C NMR (151 MHz, $CDCl_3$) of 1-(oxiran-2-ylmethyl)-1H-indole (**1O**)

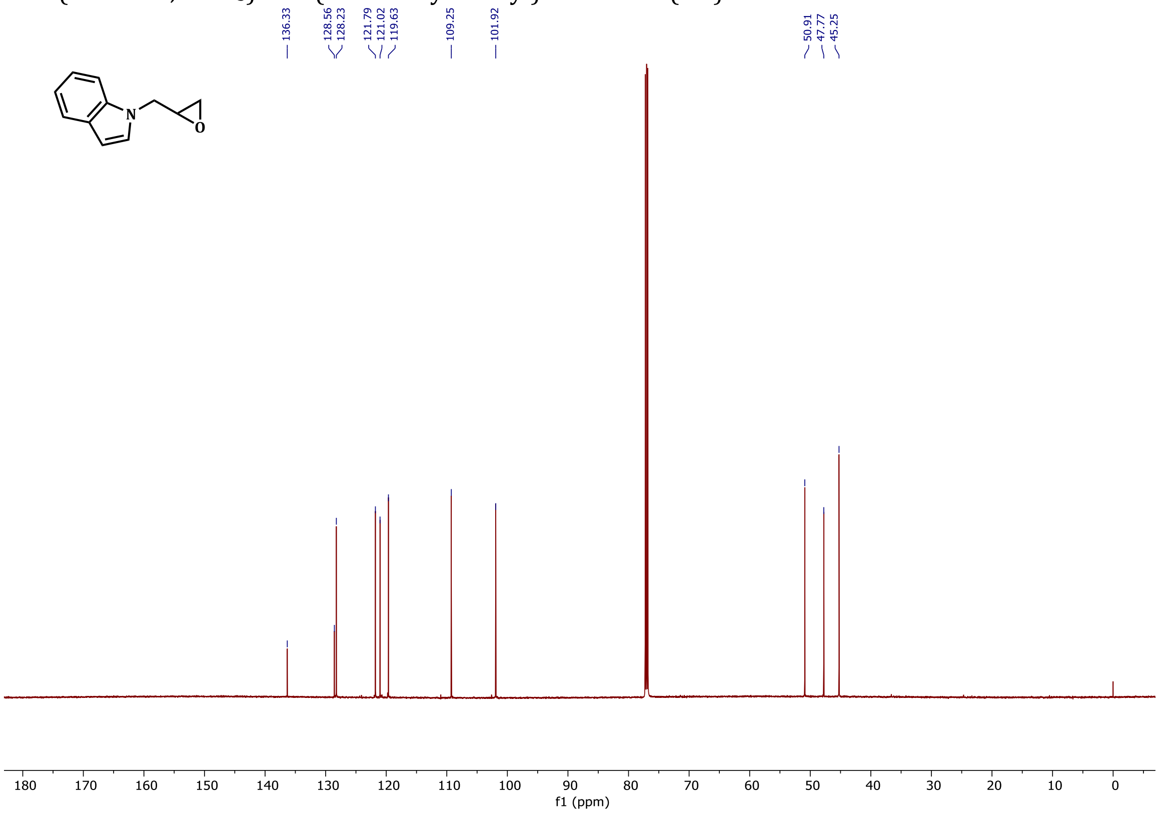

^{1}H NMR (600 MHz, $CDCl_3$) of 1,2-epoxydecane (**1P**)

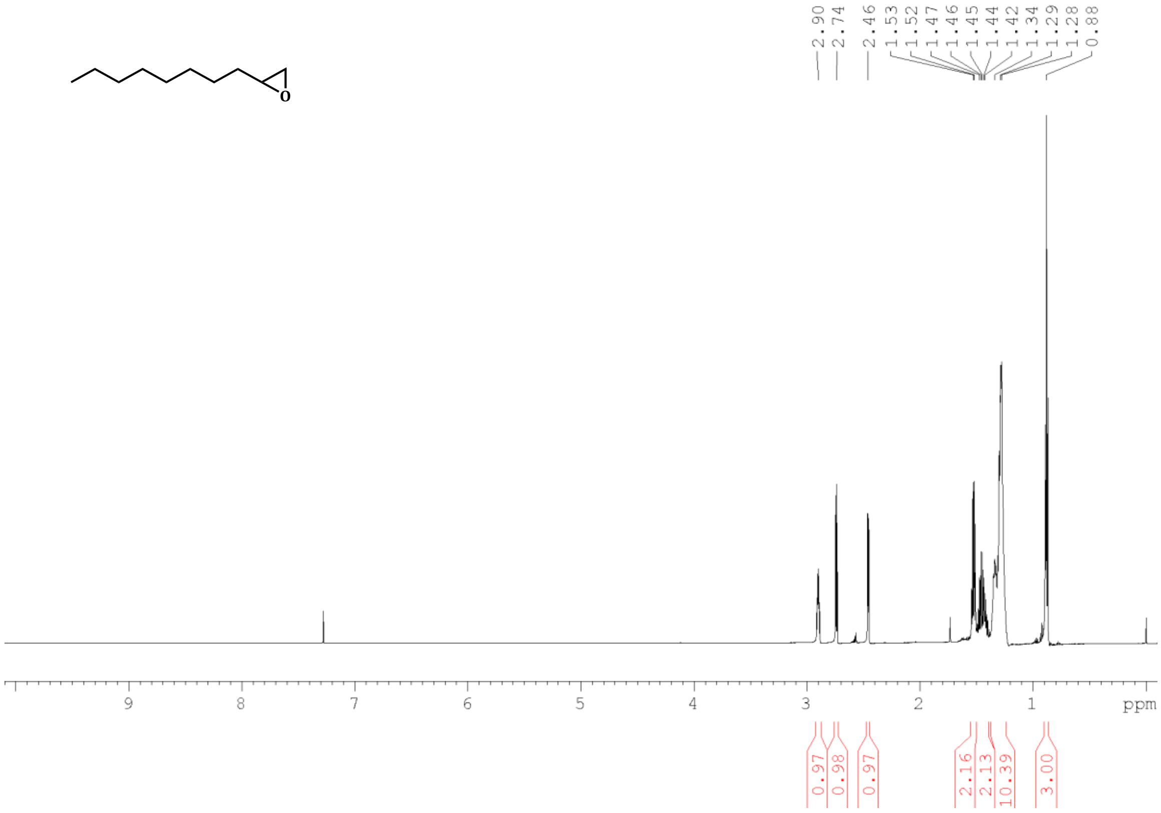

^{13}C NMR (151 MHz, $CDCl_3$) of 1,2-epoxydecane (**1P**)

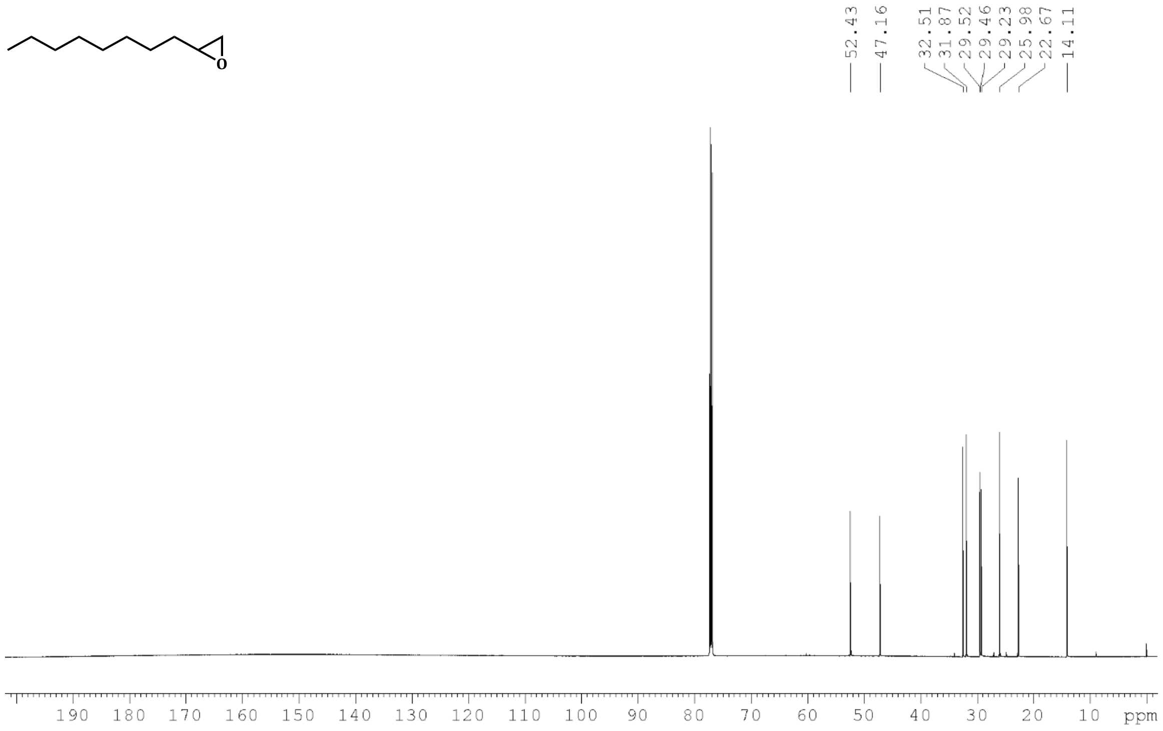

^{1}H NMR (600 MHz, $CDCl_3$) of cyclohexyl-3 epoxy-1,2 propane (**1Q**)

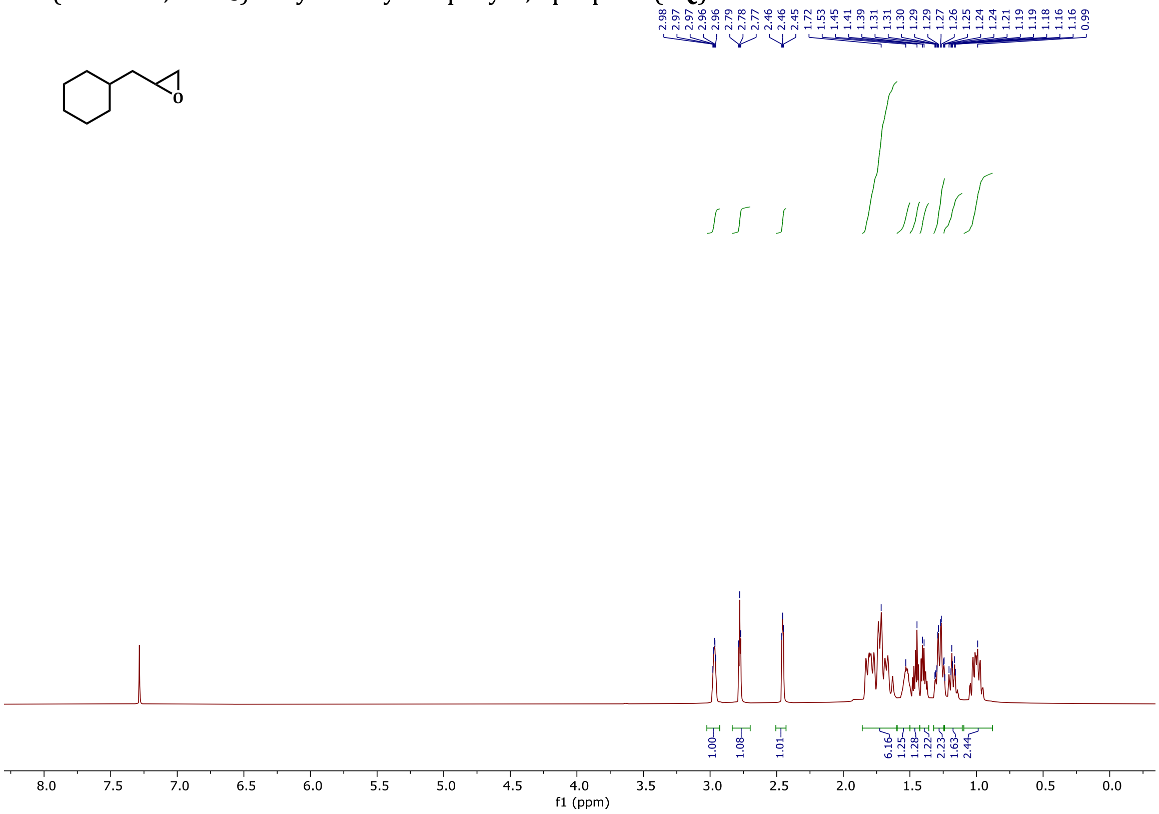

^{13}C NMR (151 MHz, $CDCl_3$) of cyclohexyl-3 epoxy-1,2 propane (**1Q**)

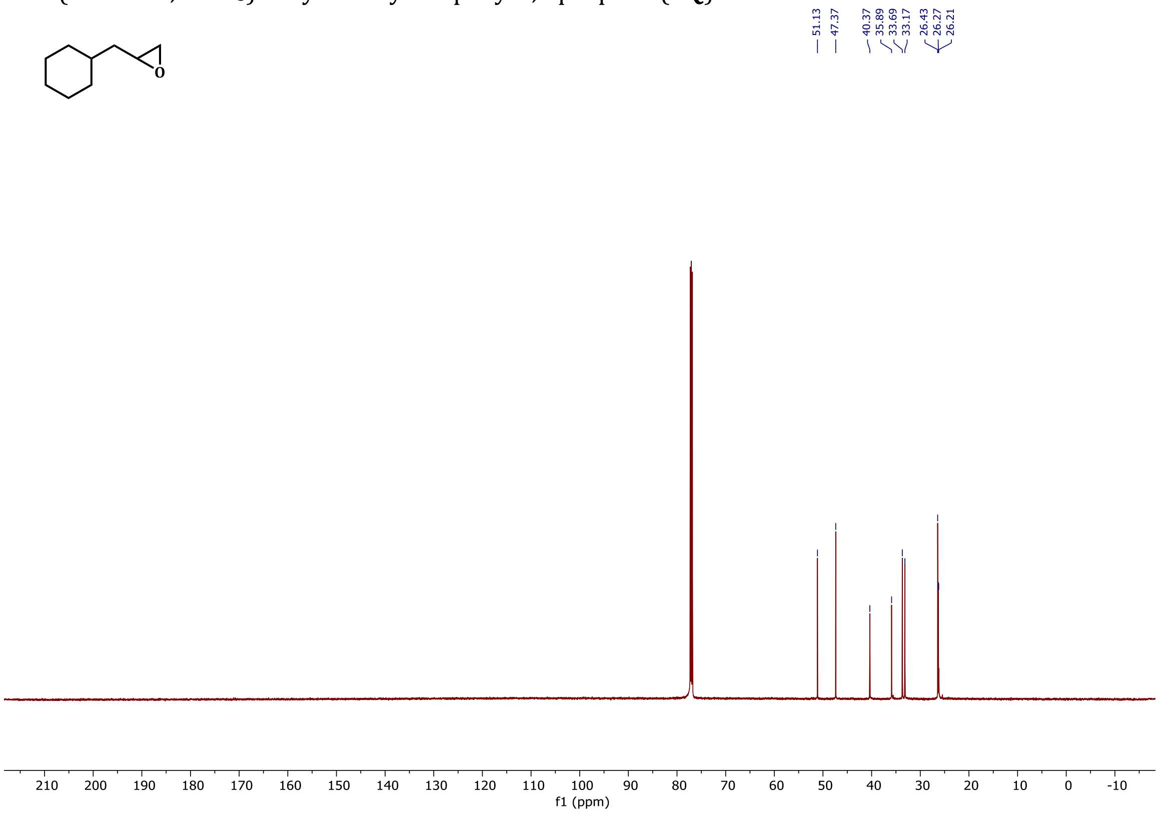

^{1}H NMR (600 MHz, $CDCl_3$) of 2-benzyloxymethyloxetane (**S11**)

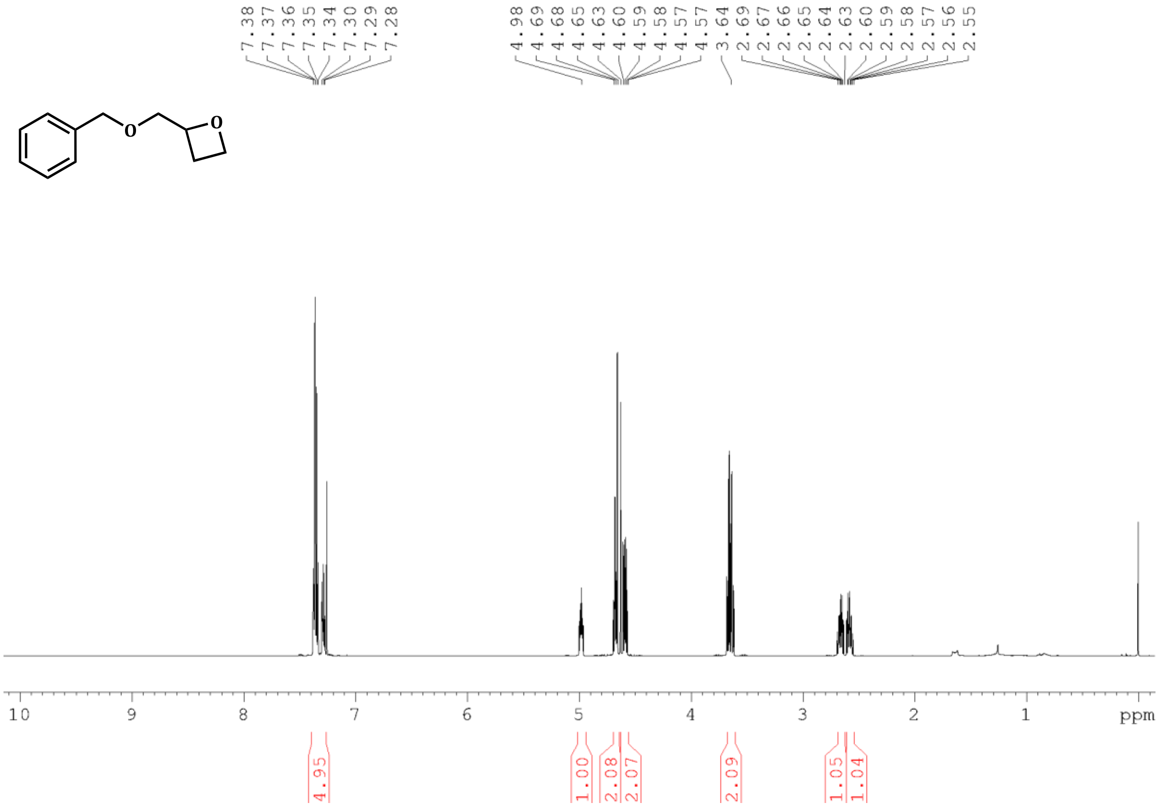

^{13}C NMR (151 MHz, $CDCl_3$) of 2-benzyloxymethyloxetane (**S11**)

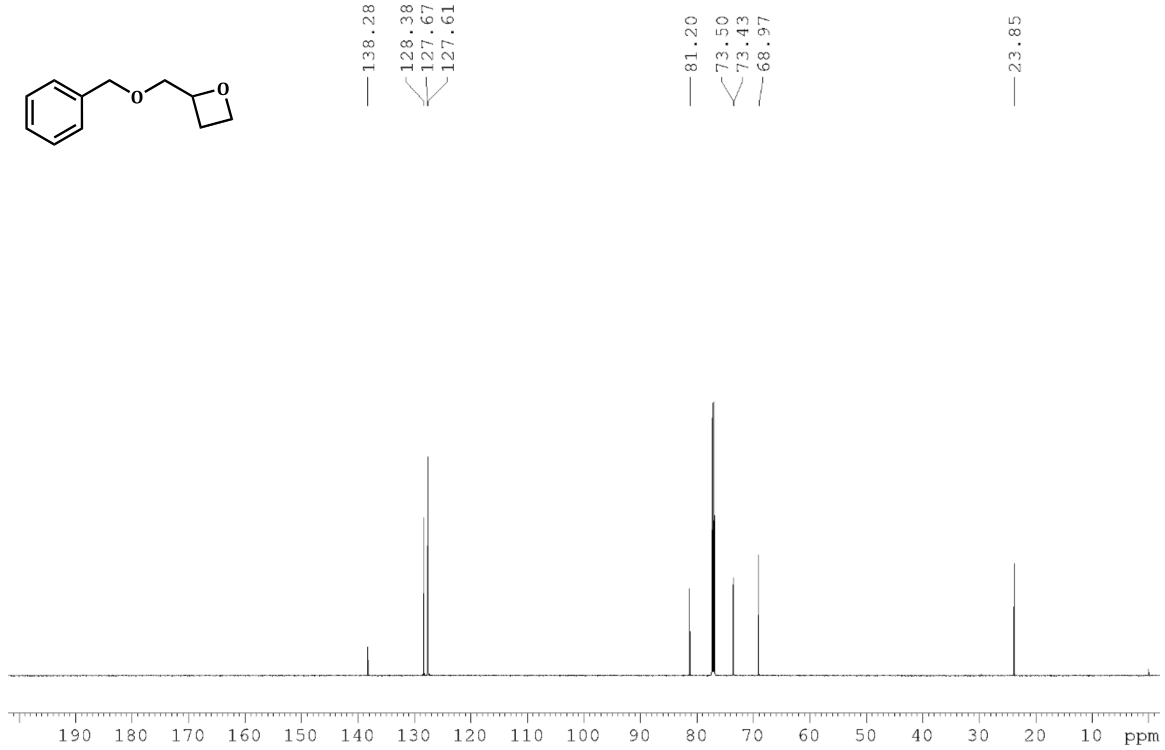

^{1}H NMR (600 MHz, $CDCl_3$) of 3-phenyl-2-propanol (**2A**)

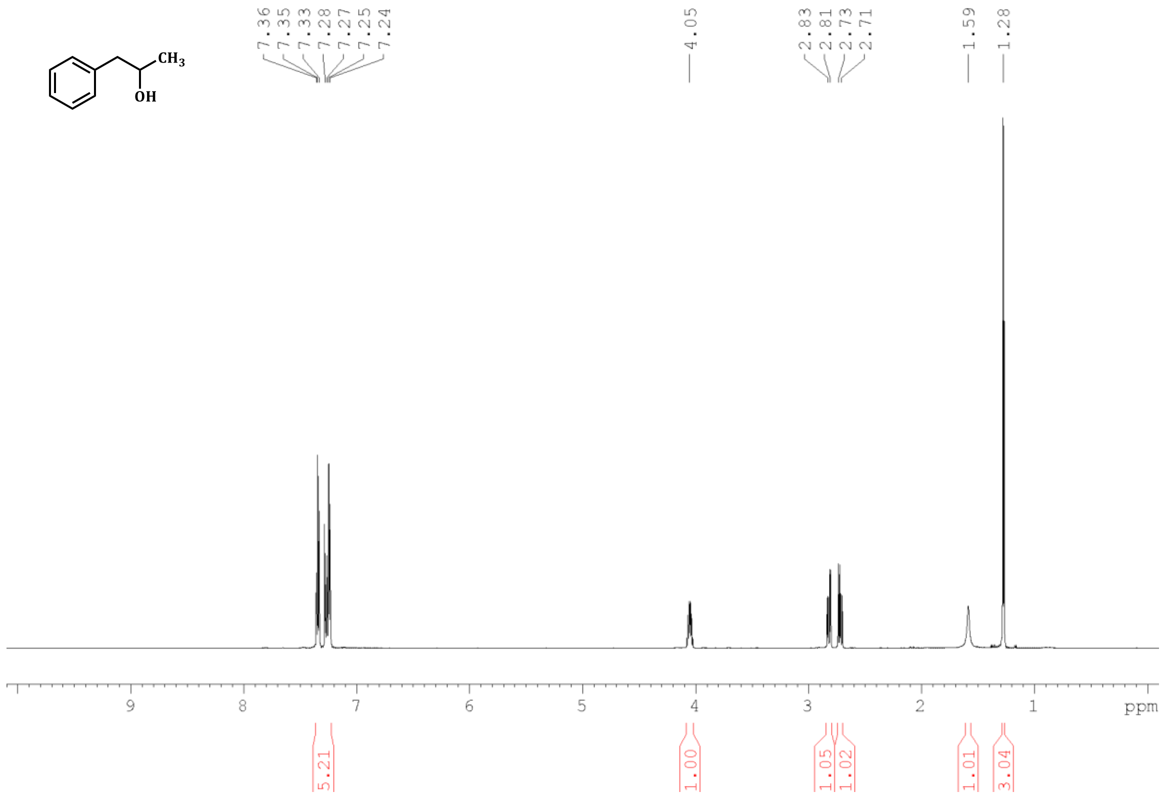

^{13}C NMR (151 MHz, $CDCl_3$) of 3-phenyl-2-propanol (**2A**)

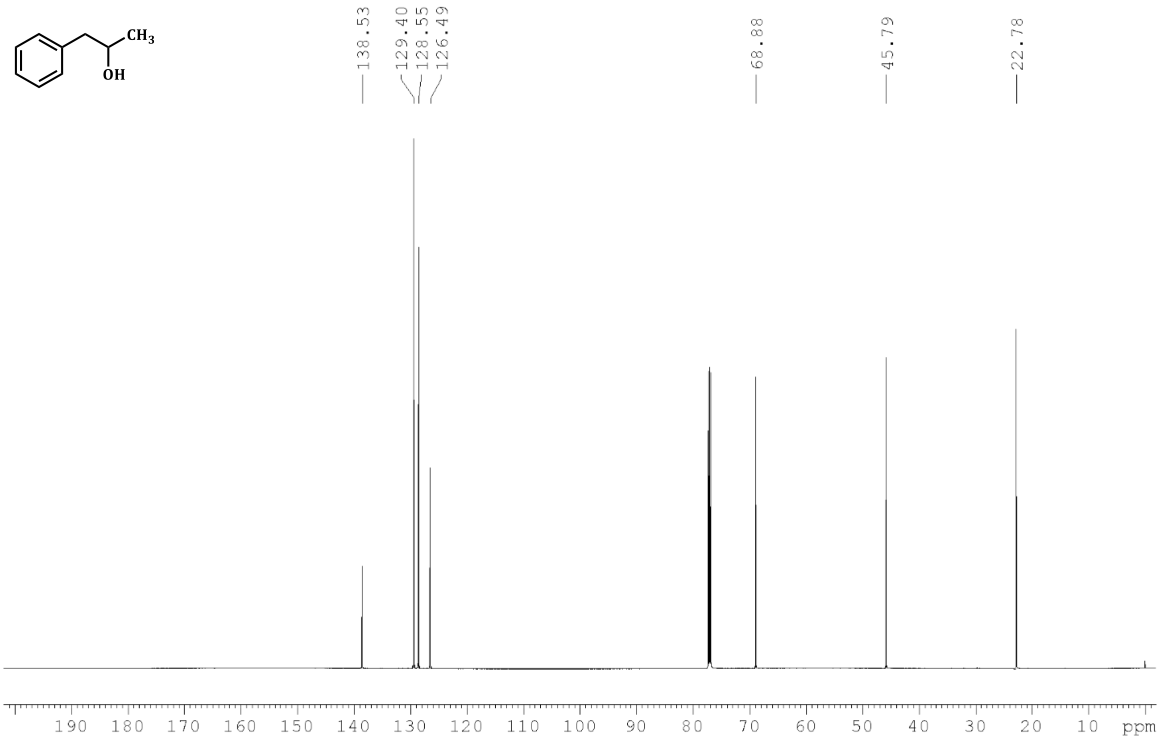

^{1}H NMR (600 MHz, $CDCl_3$) of 1-phenyl-3-butanol (**2B**)

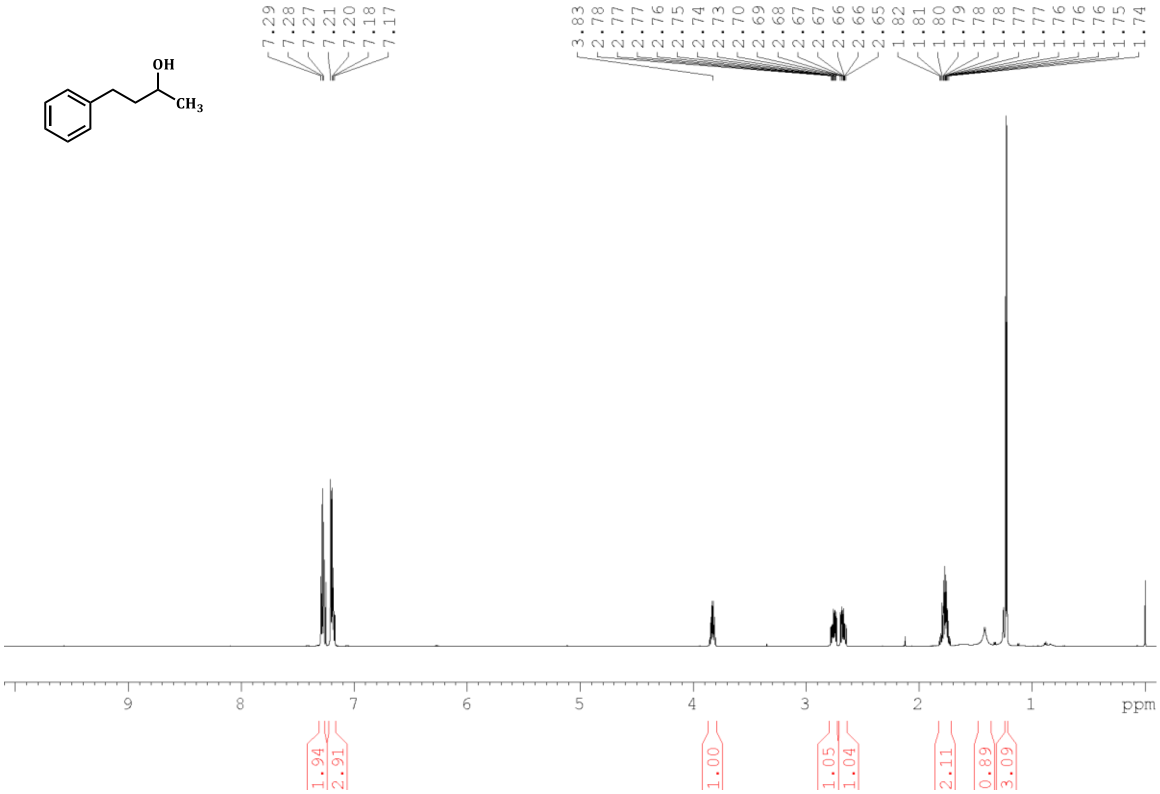

^{13}C NMR (151 MHz, $CDCl_3$) of 1-phenyl-3-butanol (**2B**)

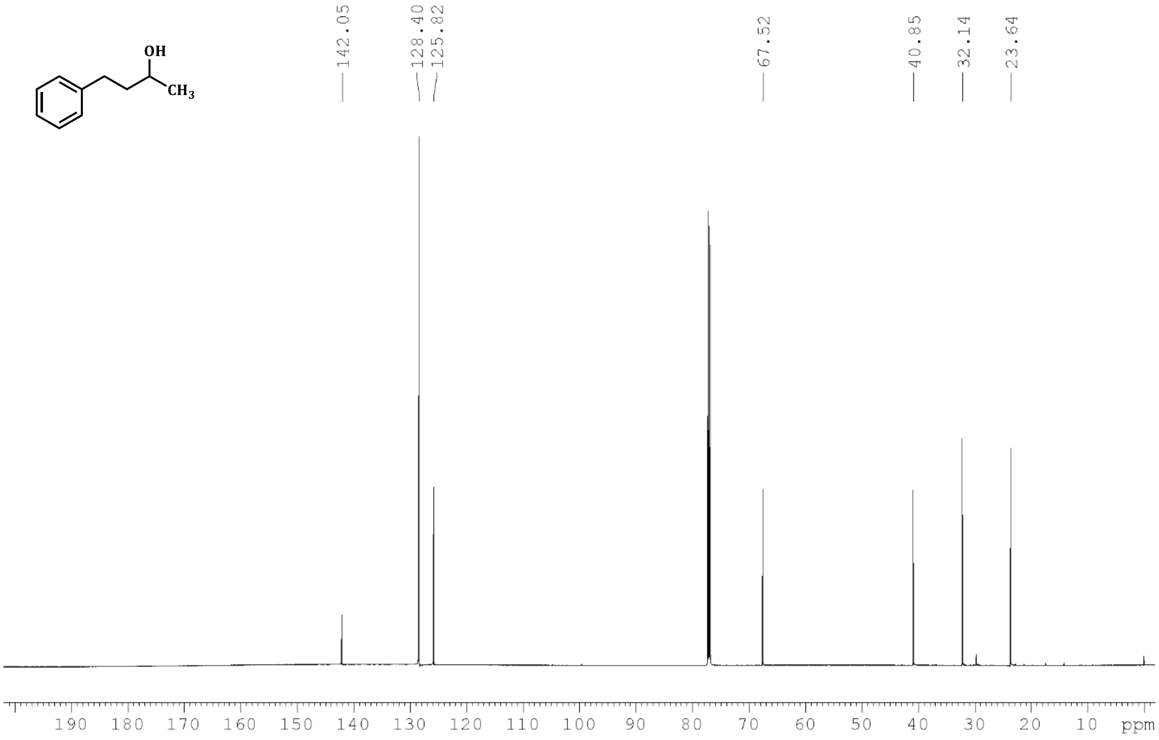

^{1}H NMR (600 MHz, $CDCl_3$) of 1-phenylethanol (**2C**)

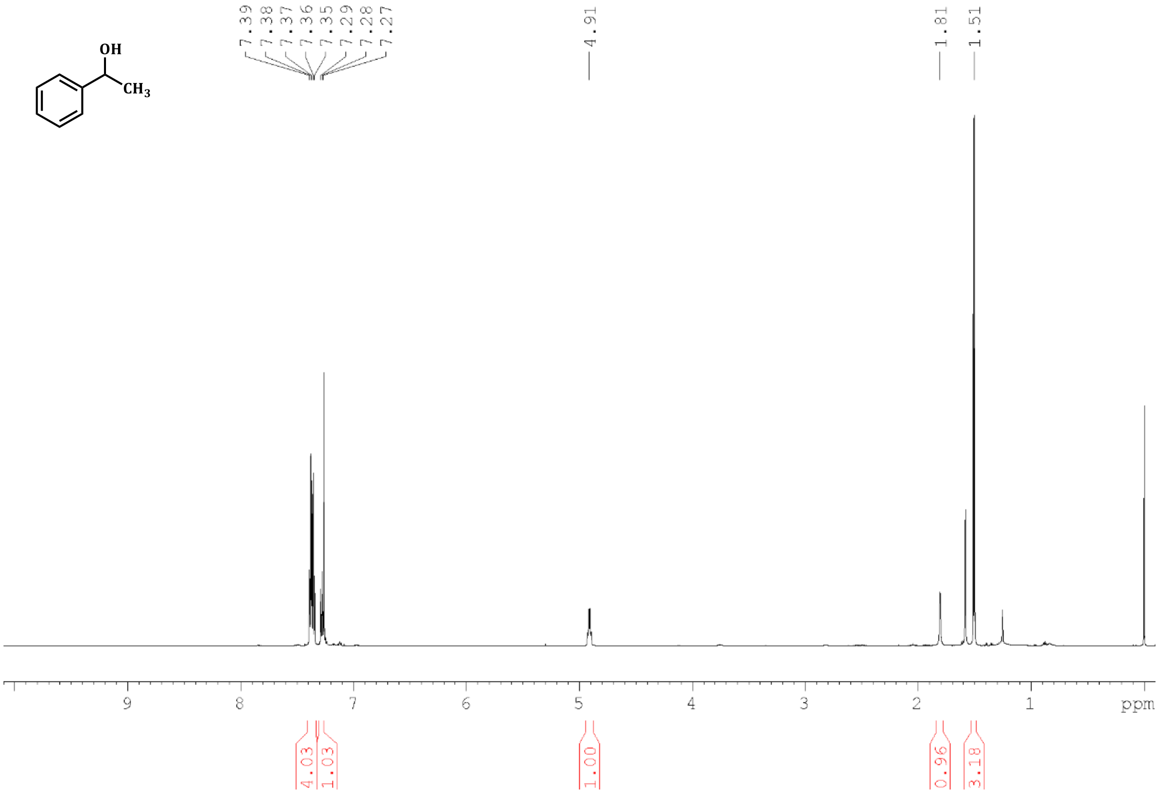

^{13}C NMR (151 MHz, $CDCl_3$) of 1-phenylethanol (**2C**)

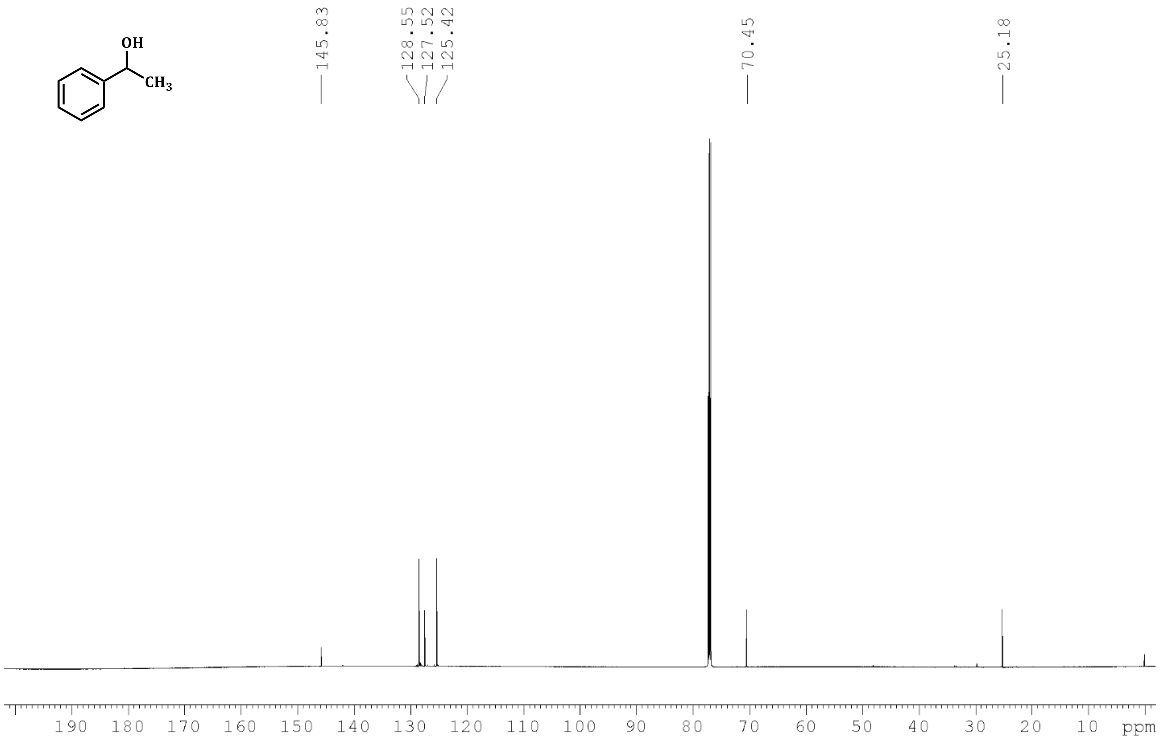

^{1}H NMR (600 MHz, $CDCl_3$) of 1-(4-methoxyphenyl)propan-2-ol (**2D**)

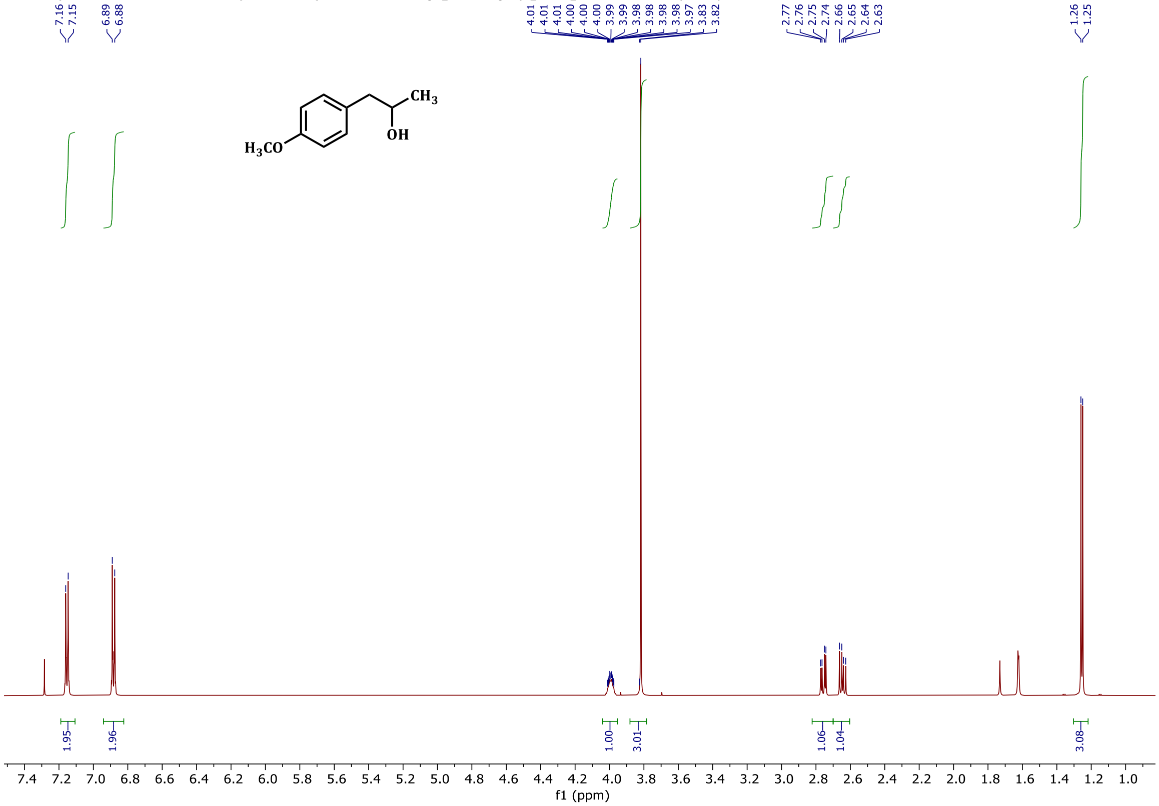

^{13}C NMR (151 MHz, $CDCl_3$) of 1-(4-methoxyphenyl)propan-2-ol (**2D**)

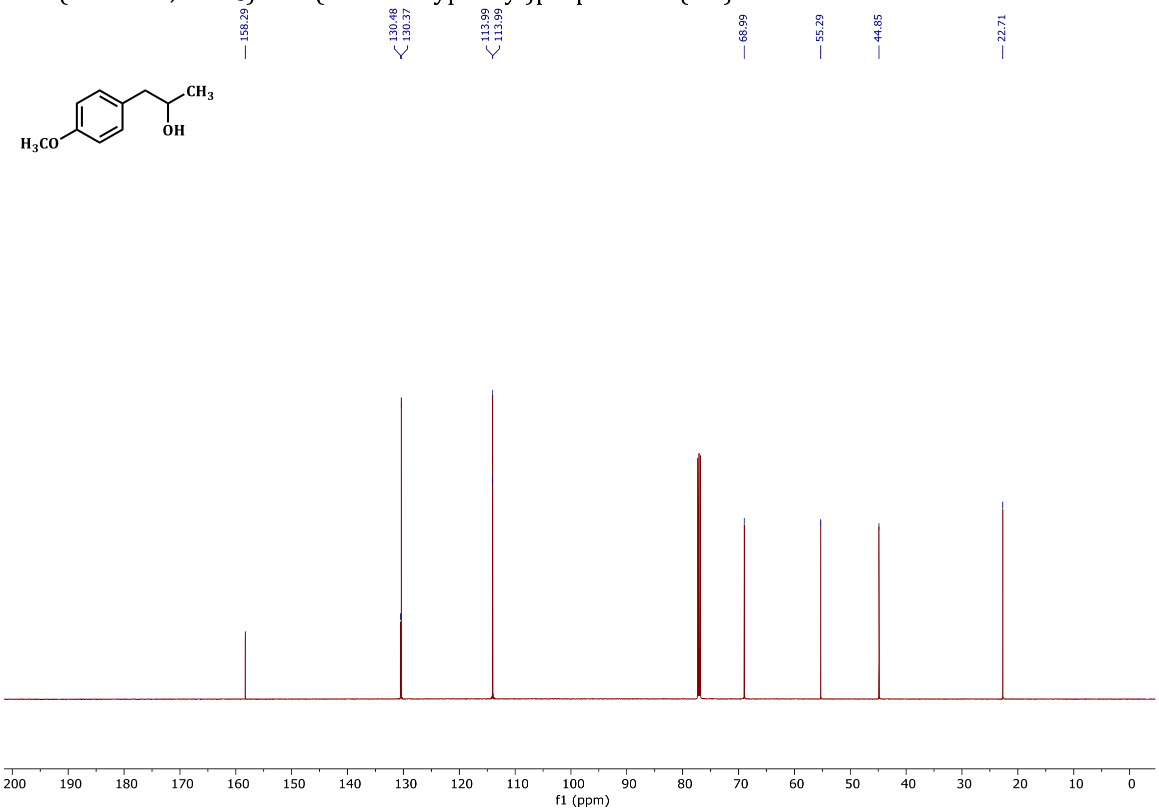

^{1}H NMR (600 MHz, $CDCl_3$) of acetic acid 4-(2-hydroxy-propyl)-2-methoxy-phenyl ester (**2E**)

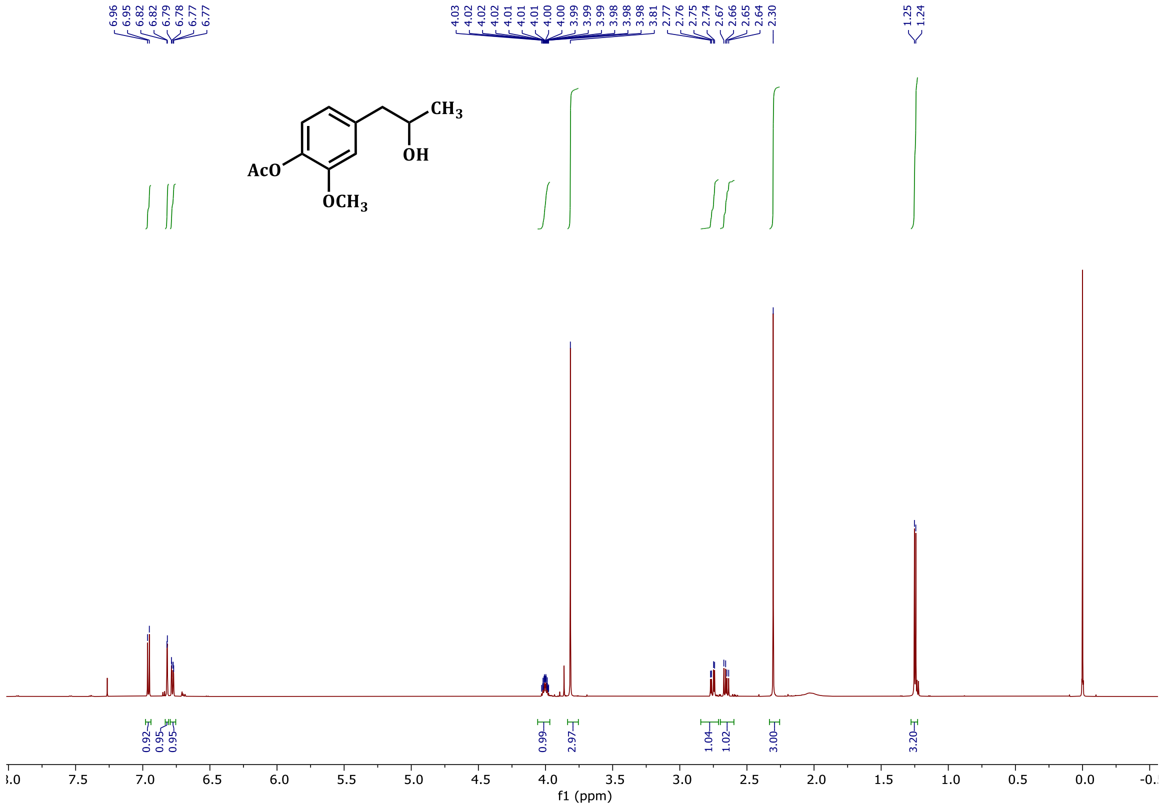

^{13}C NMR (151 MHz, $CDCl_3$) of acetic acid 4-(2-hydroxy-propyl)-2-methoxy-phenyl ester (**2E**)

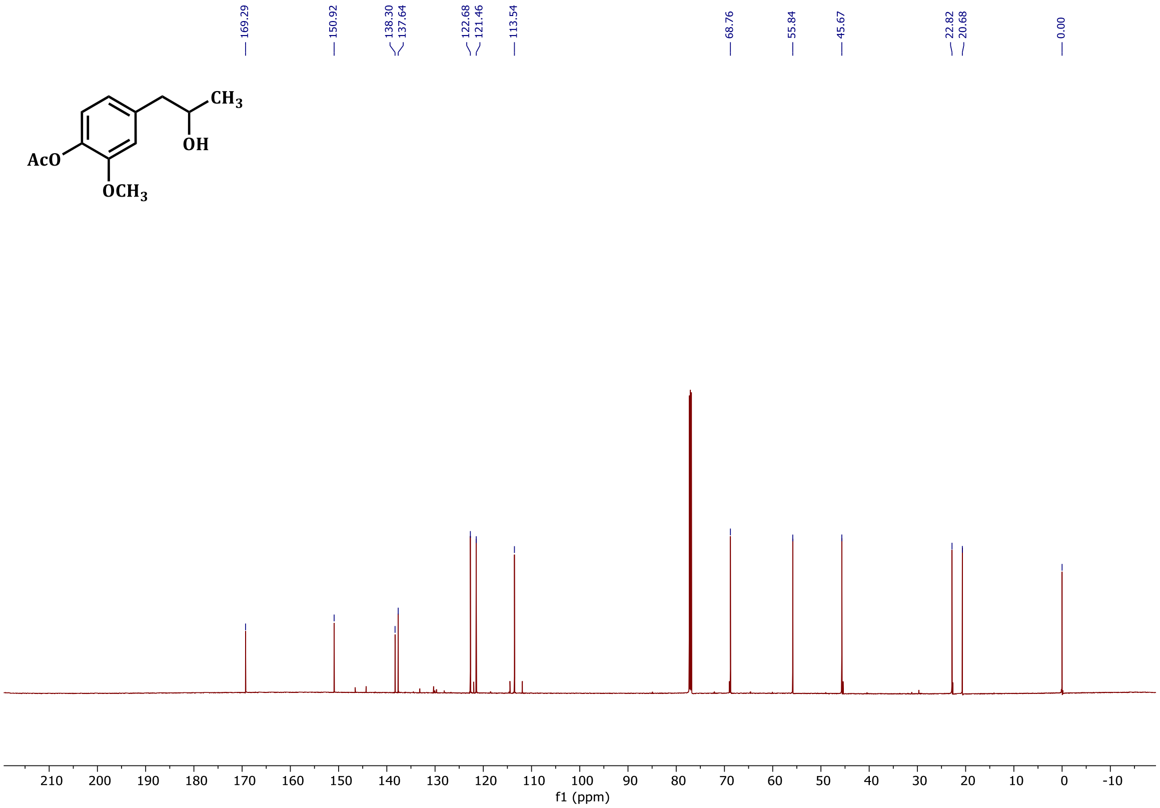

^{1}H NMR (600 MHz, $CDCl_3$) of 4-(2-hydroxypropyl)-2-methoxyphenol (**2E'**)

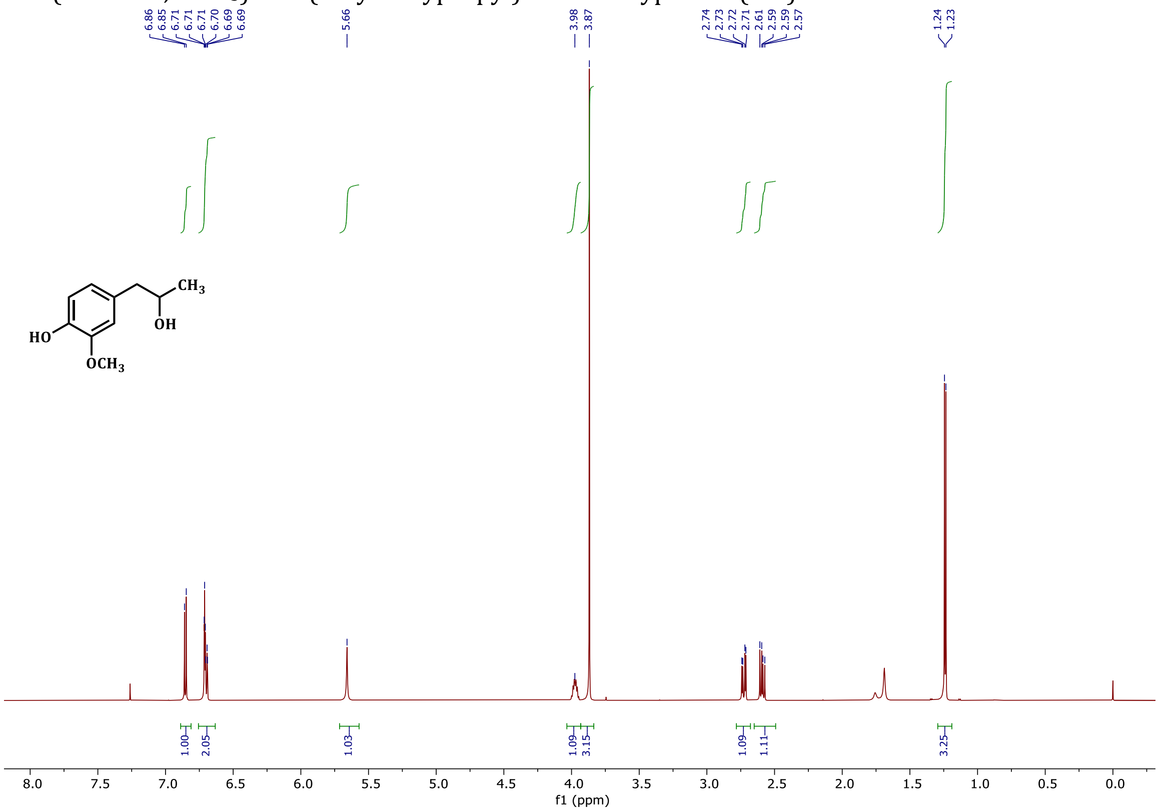

^{13}C NMR (151 MHz, $CDCl_3$) of 4-(2-hydroxypropyl)-2-methoxyphenol (**2E'**)

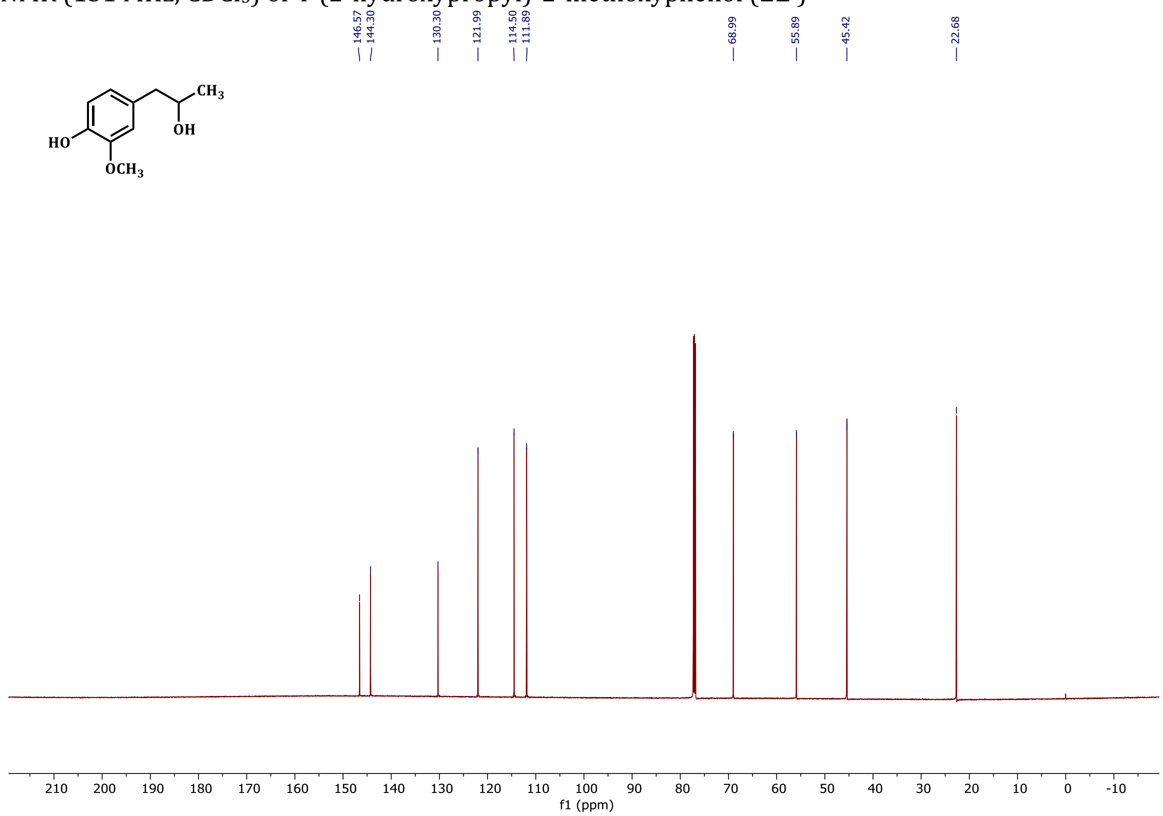

^{1}H NMR (600 MHz, $CDCl_3$) of 1-phenoxy-2-propanol (**2F**)

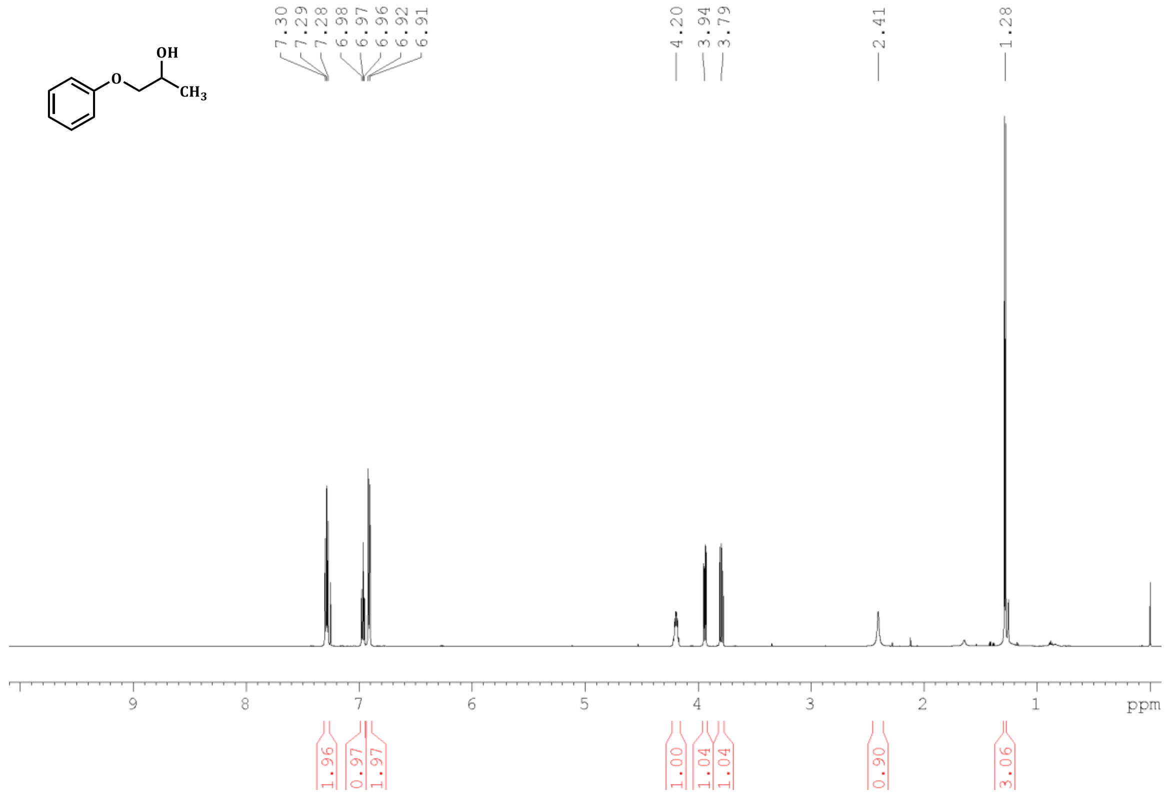

^{13}C NMR (151 MHz, $CDCl_3$) of 1-phenoxy-2-propanol (**2F**)

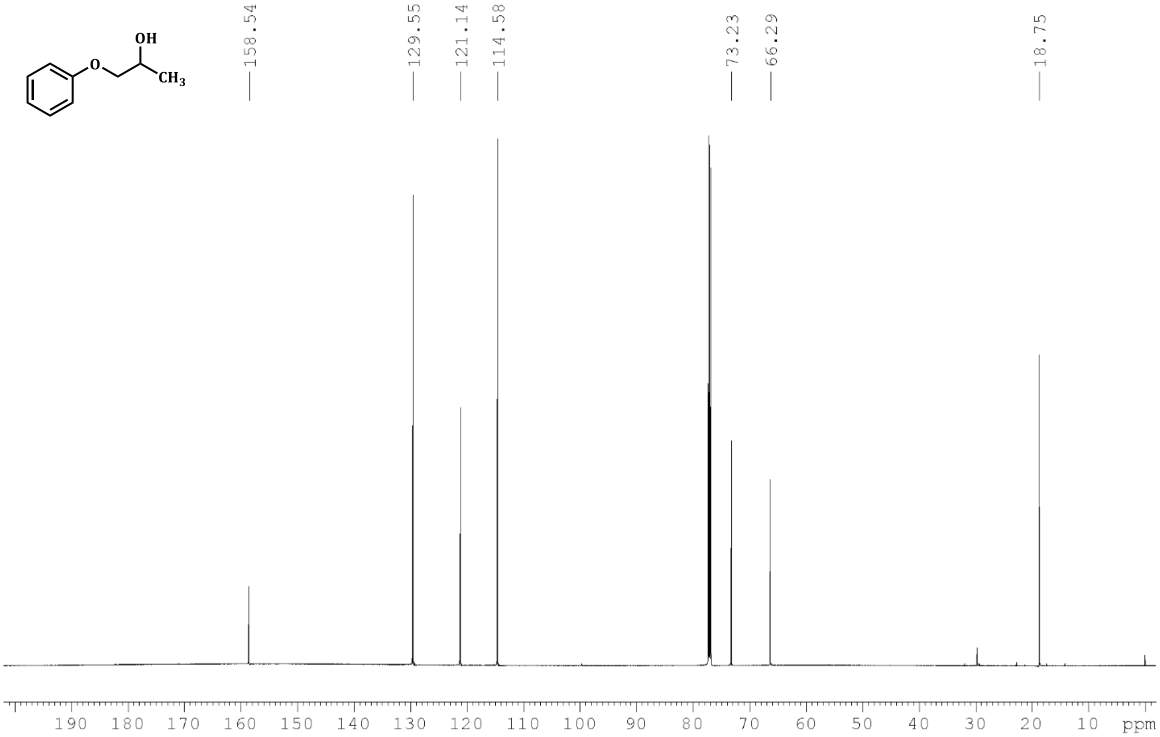

^{1}H NMR (600 MHz, $CDCl_3$) of 2-hydroxy-3-(4'-tert-butylphenoxy) propane (**2G**)

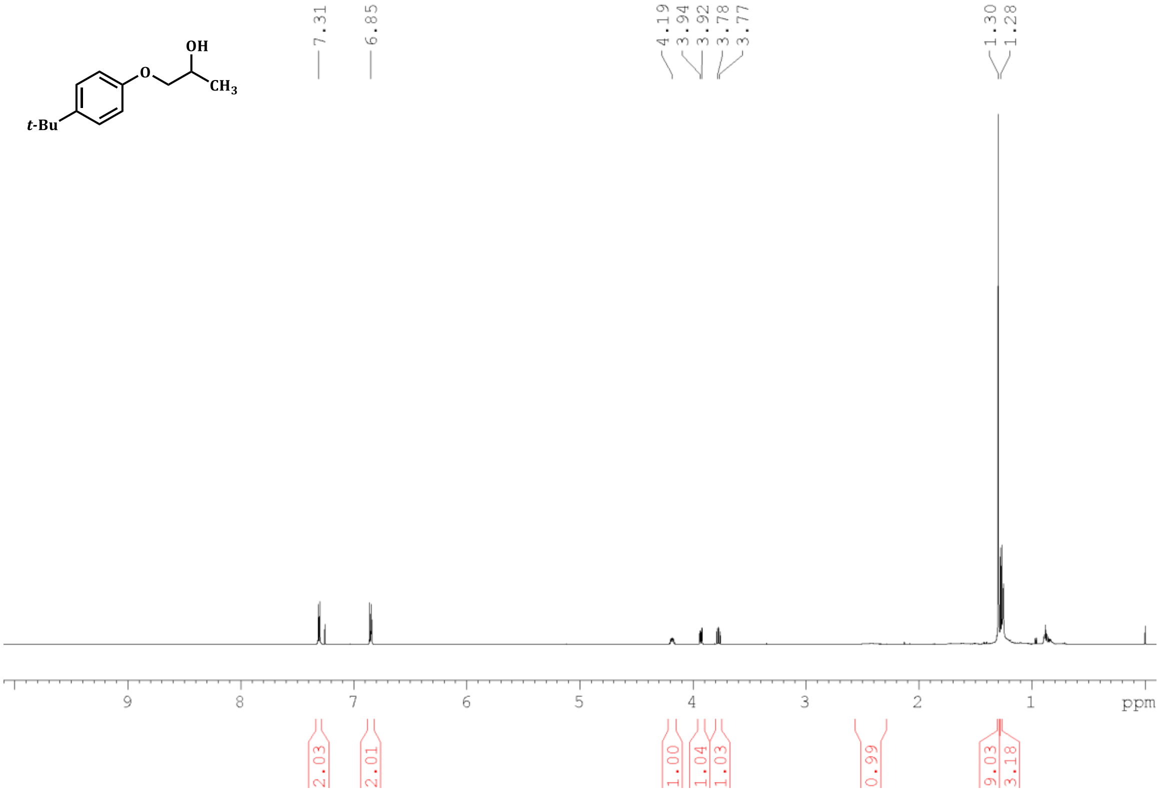

^{13}C NMR (151 MHz, $CDCl_3$) of 2-hydroxy-3-(4'-tert-butylphenoxy) propane (**2G**)

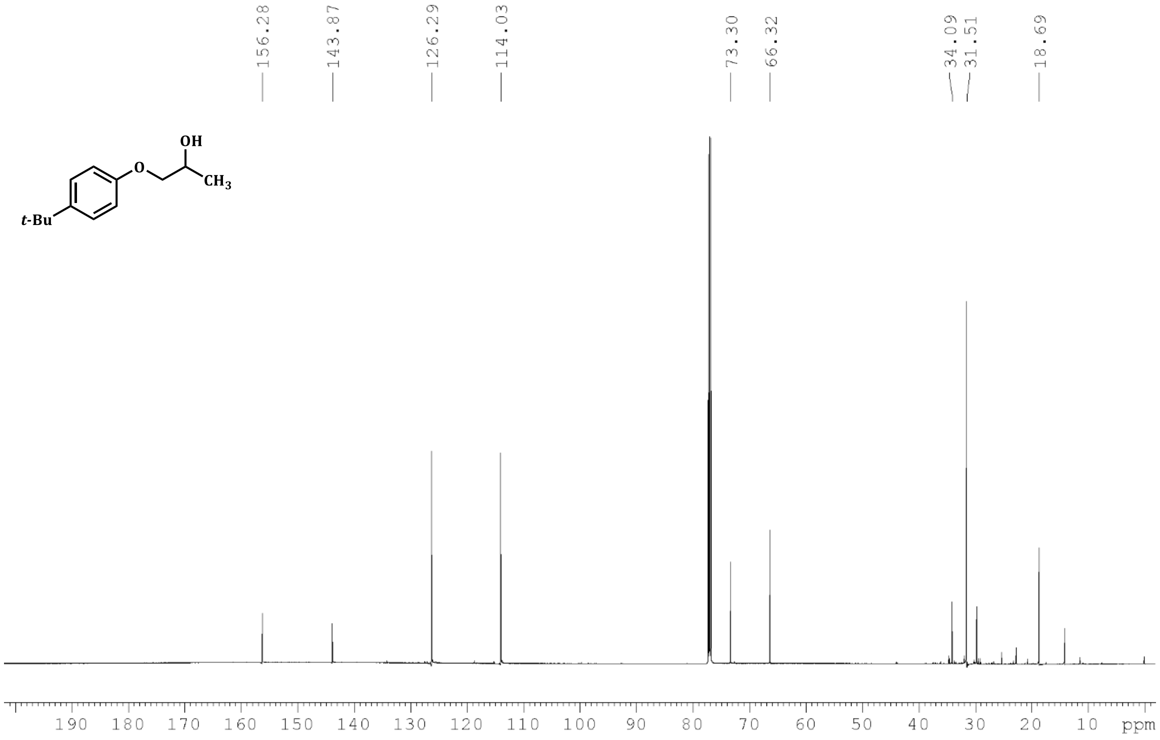

^{1}H NMR (600 MHz, $CDCl_3$) of 4-(2-hydroxypropoxy) benzonitrile (**2H**)

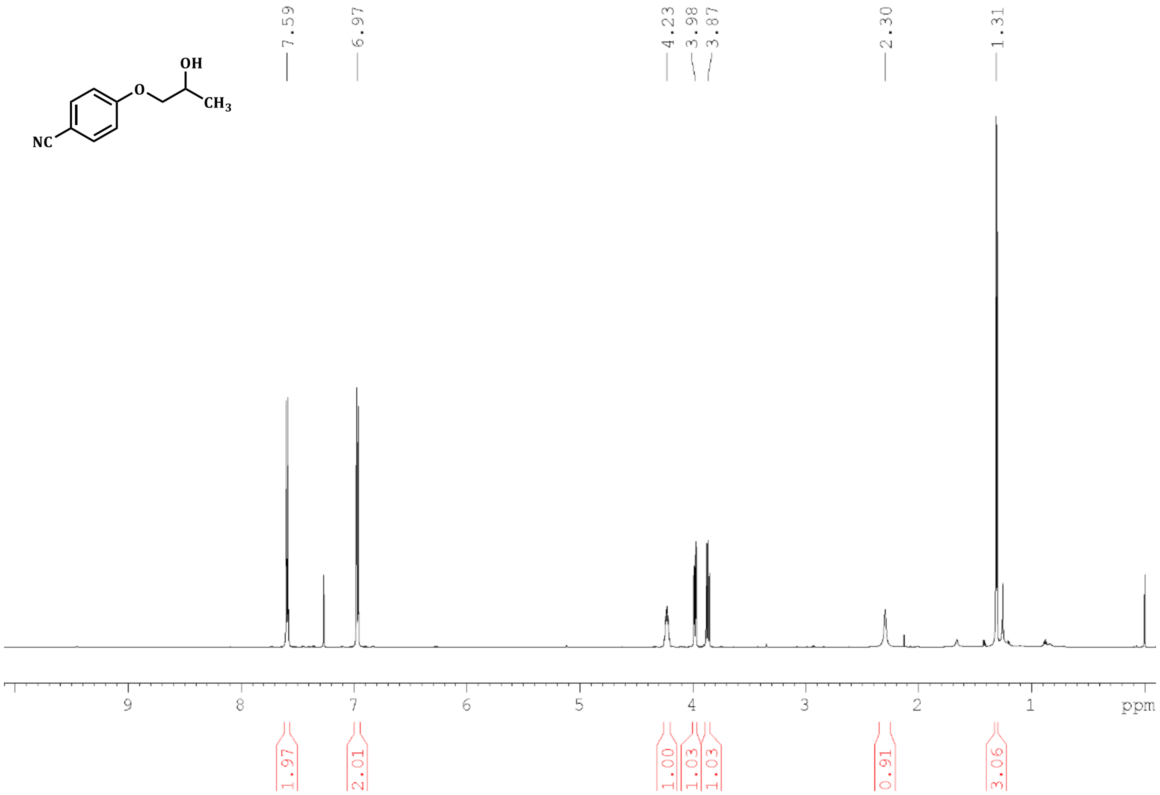

^{13}C NMR (151 MHz, $CDCl_3$) of 4-(2-hydroxypropoxy) benzonitrile (**2H**)

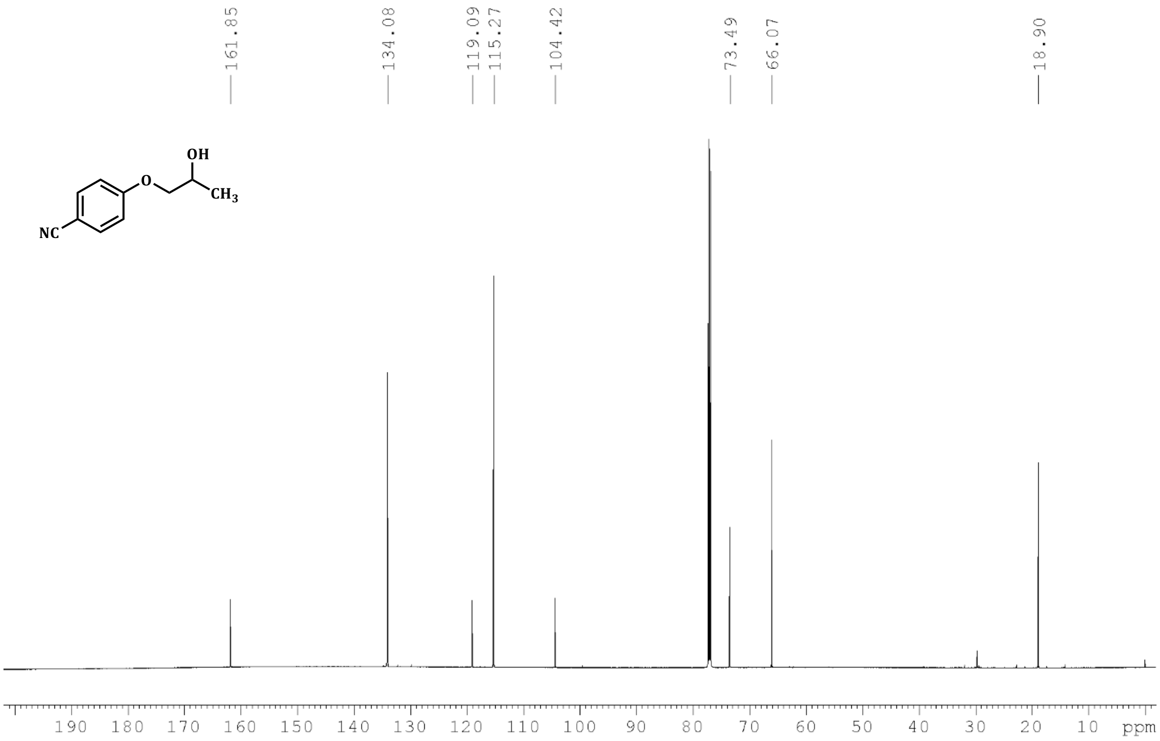

^{1}H NMR (600 MHz, $CDCl_3$) of 1-(4-(trifluoromethyl)phenoxy) propan-2-ol (**2I**)

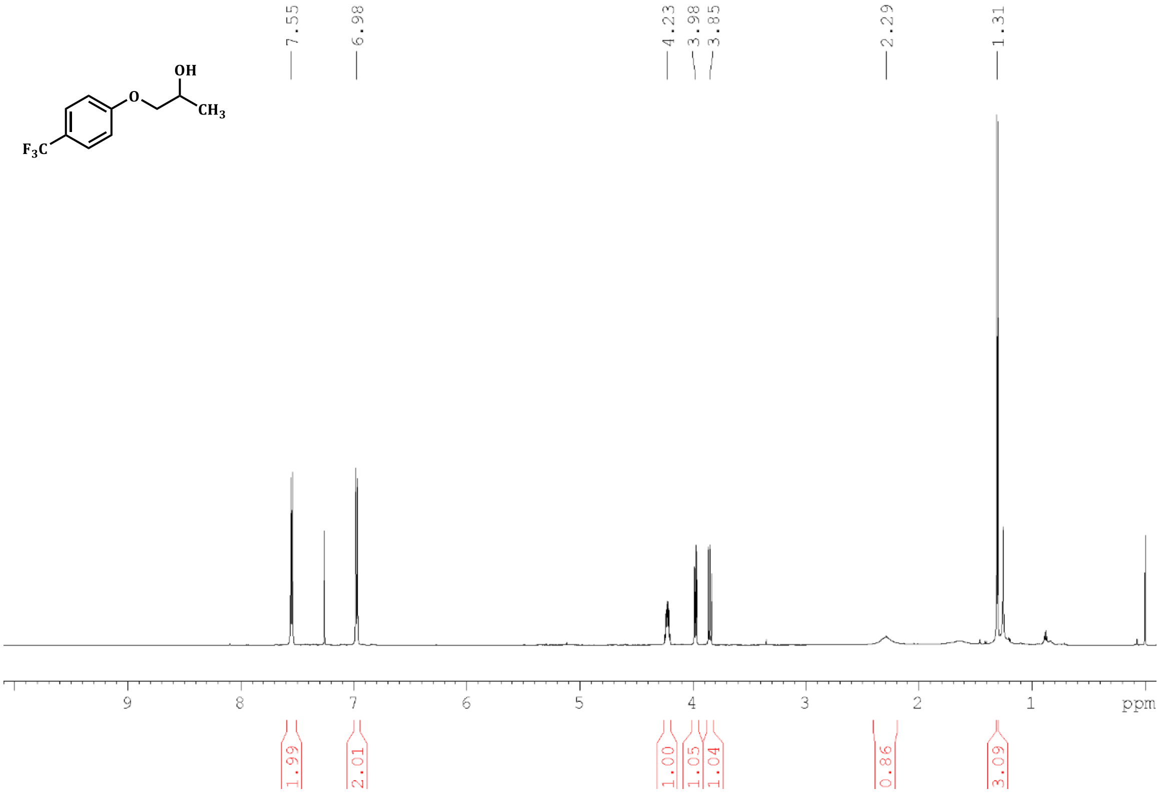

^{13}C NMR (151 MHz, $CDCl_3$) of 1-(4-(trifluoromethyl)phenoxy) propan-2-ol (**2I**)

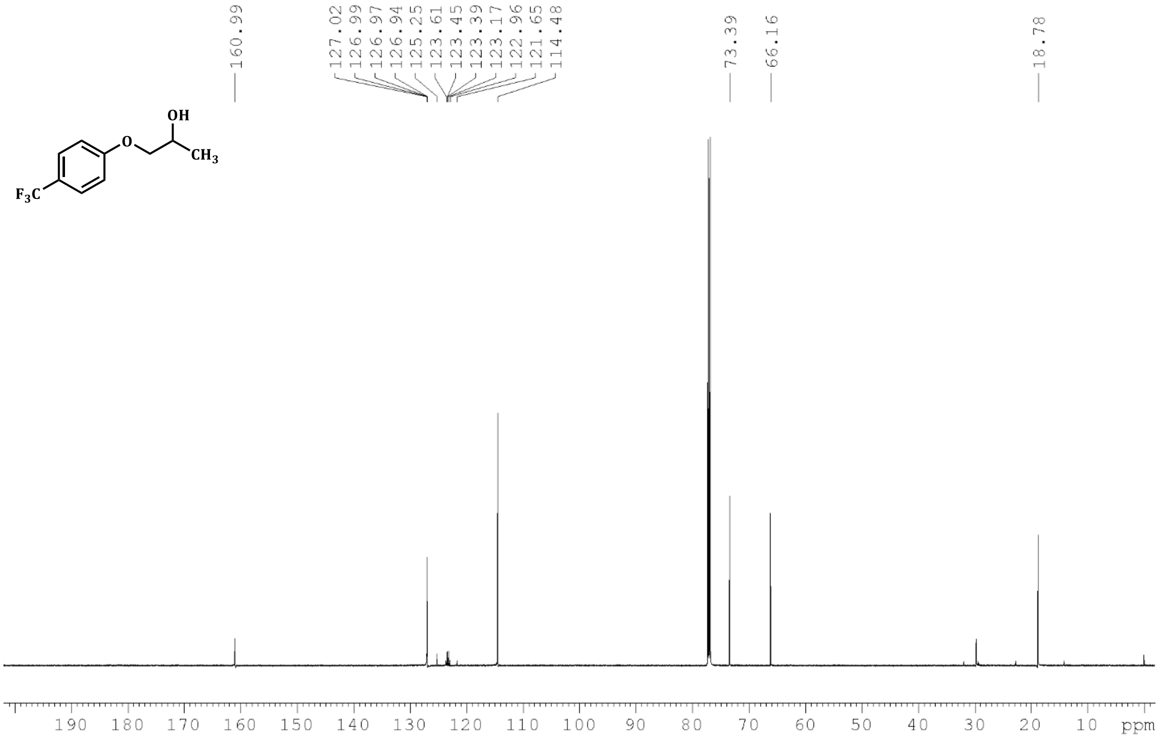

^{19}F NMR (594 MHz, $CDCl_3$) of 1-(4-(trifluoromethyl)phenoxy) propan-2-ol (**2I**)

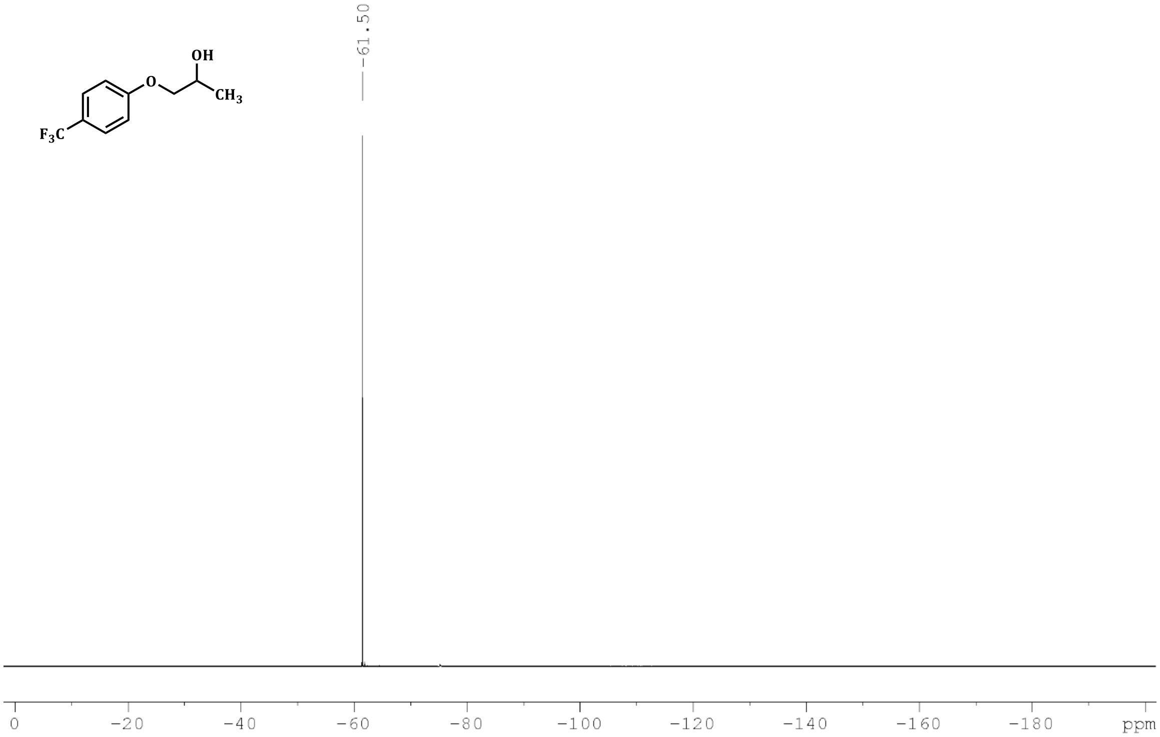

^1H NMR (600 MHz, $CDCl_3$) of benzyloxy-2-propanol (**2J**)

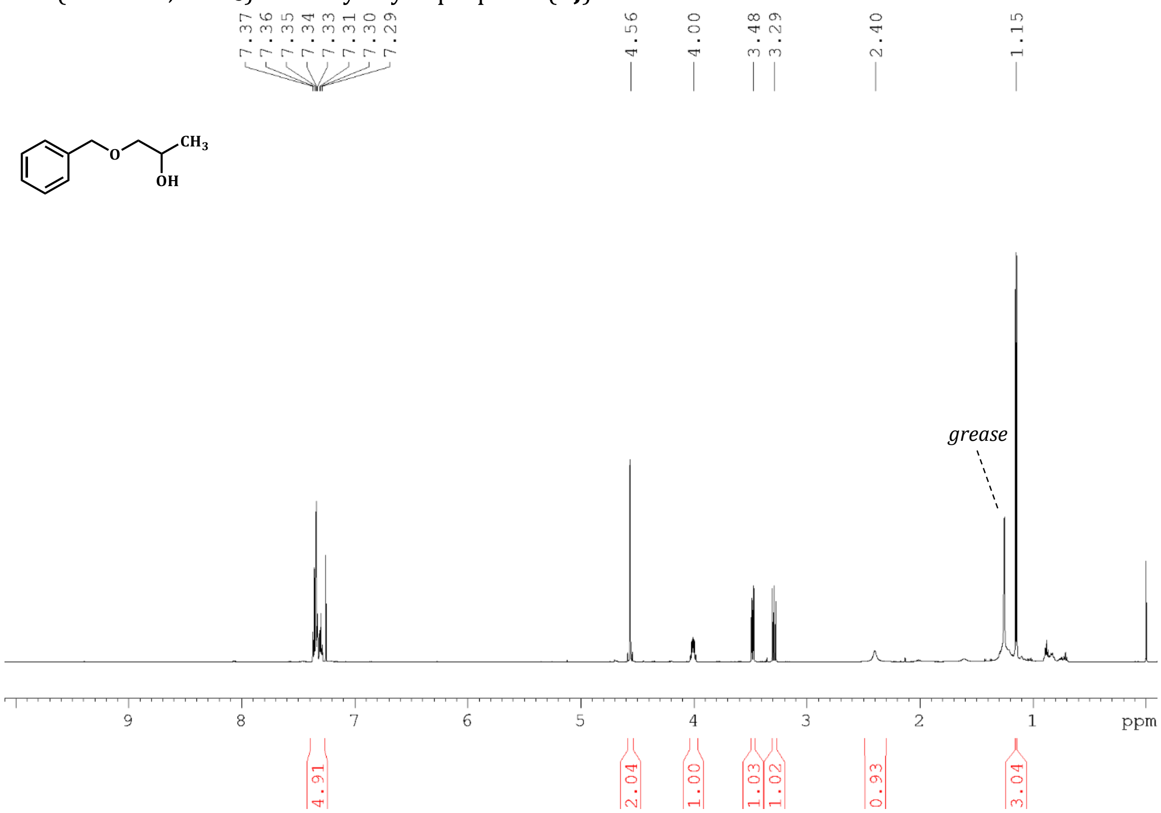

^{13}C NMR (151 MHz, $CDCl_3$) of benzyloxy-2-propanol (**2J**)

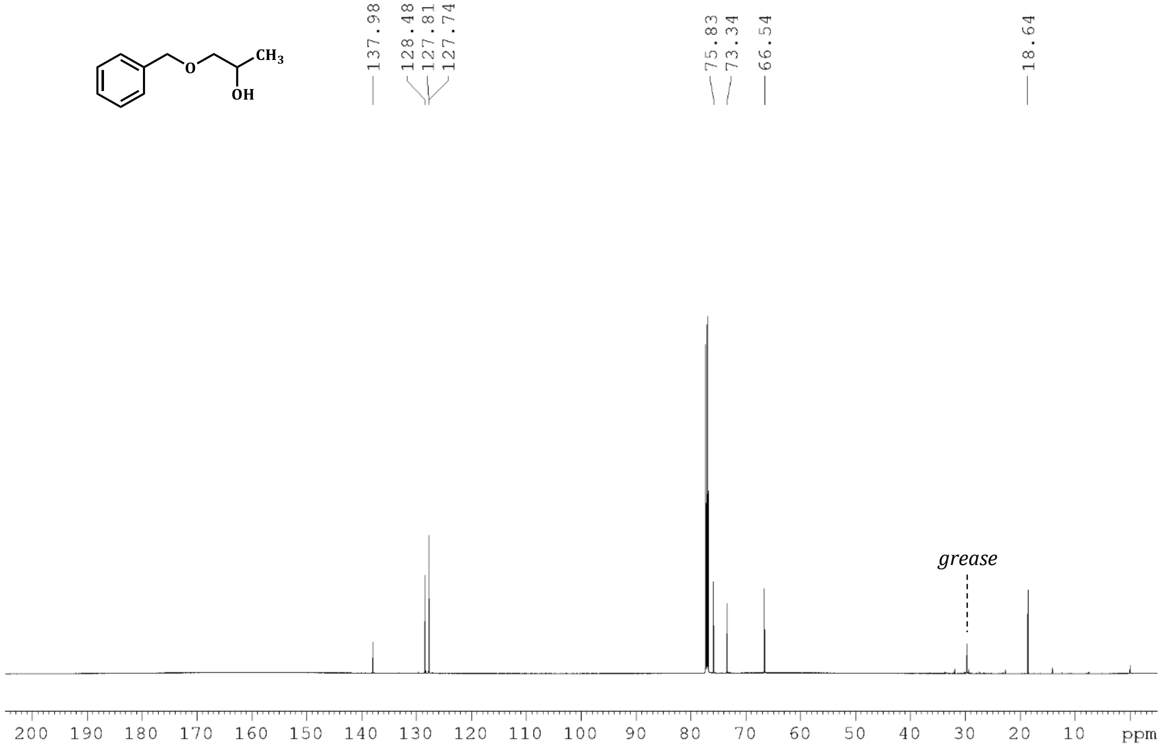

^{1}H NMR (600 MHz, $CDCl_3$) of benzyl 10-hydroxyundecanoate (**2K**)

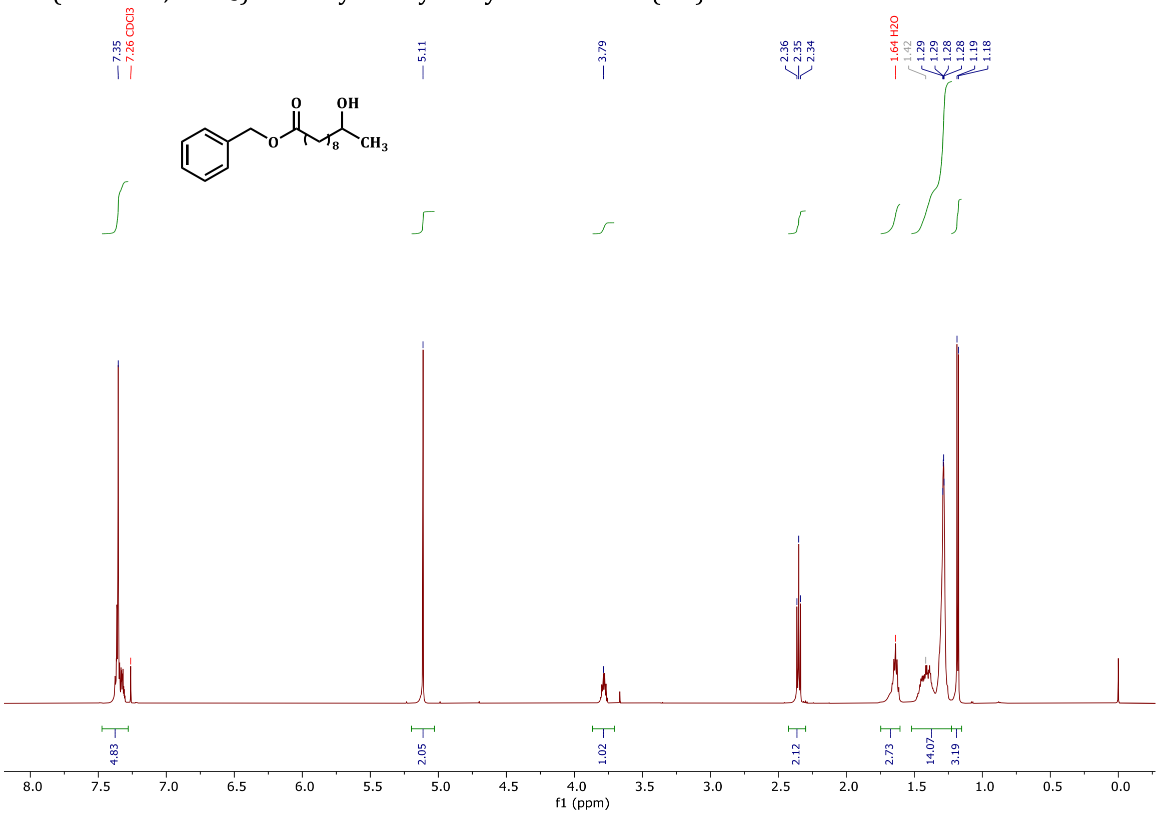

^{13}C NMR (151 MHz, $CDCl_3$) of benzyl 10-hydroxyundecanoate (**2K**)

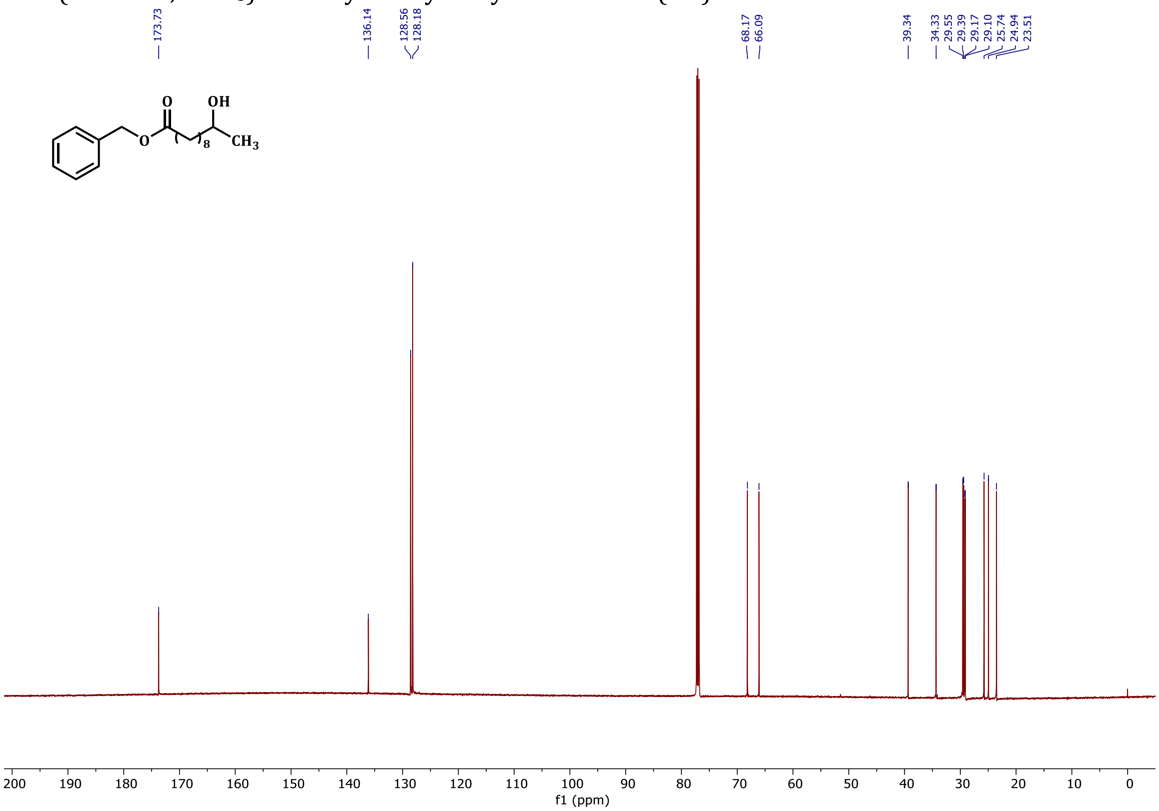

^{1}H NMR (600 MHz, $CDCl_3$) of 1-(phenylthio)propan-2-ol (**2L**)

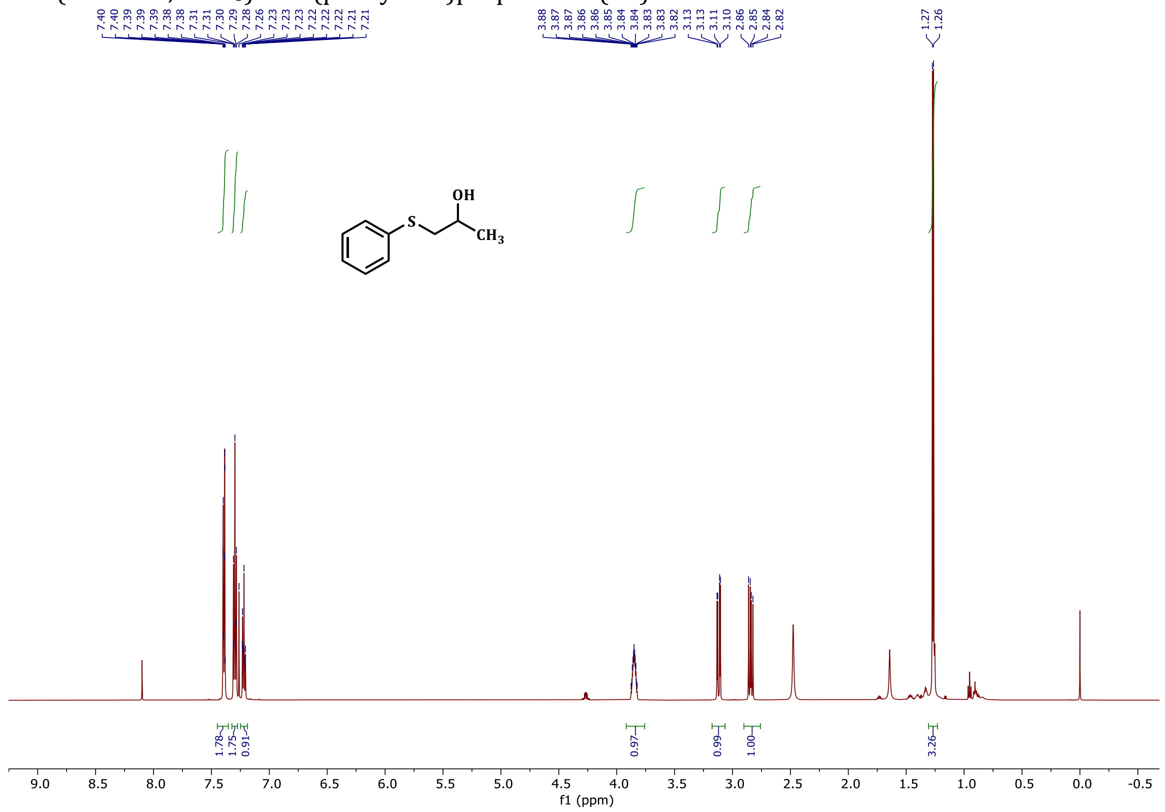

^{13}C NMR (151 MHz, $CDCl_3$) of 1-(phenylthio)propan-2-ol (**2L**)

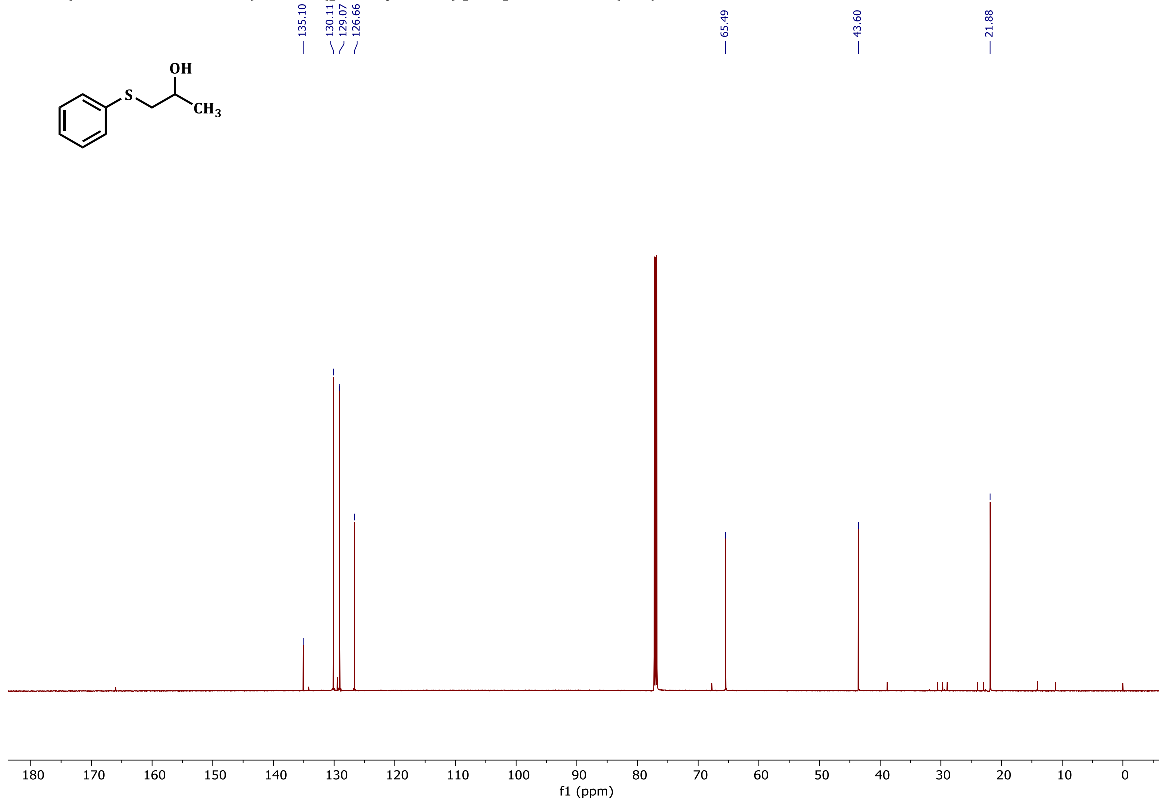

^{1}H NMR (600 MHz, $CDCl_3$) of 3-hydroxy-1-phenyl-butan-1-one (**2M**)

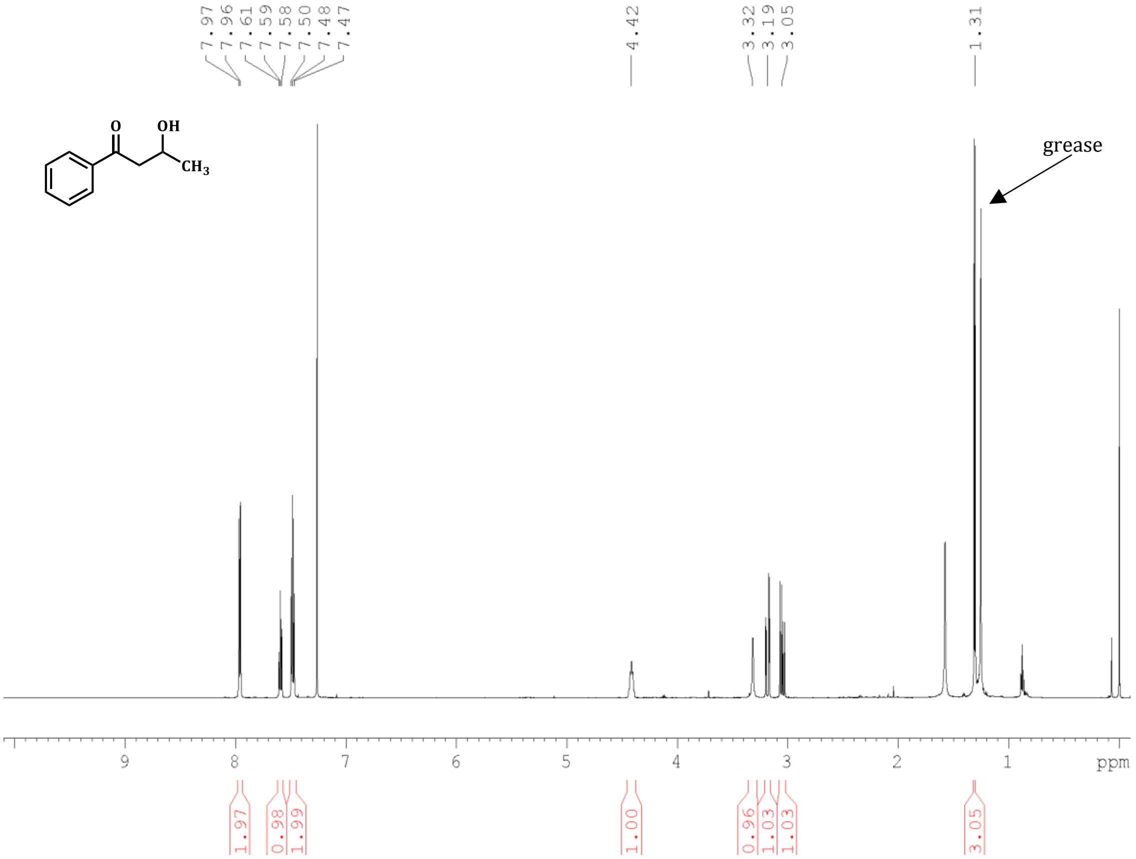

^{13}C NMR (151 MHz, $CDCl_3$) of 3-hydroxy-1-phenyl-butan-1-one (**2M**)

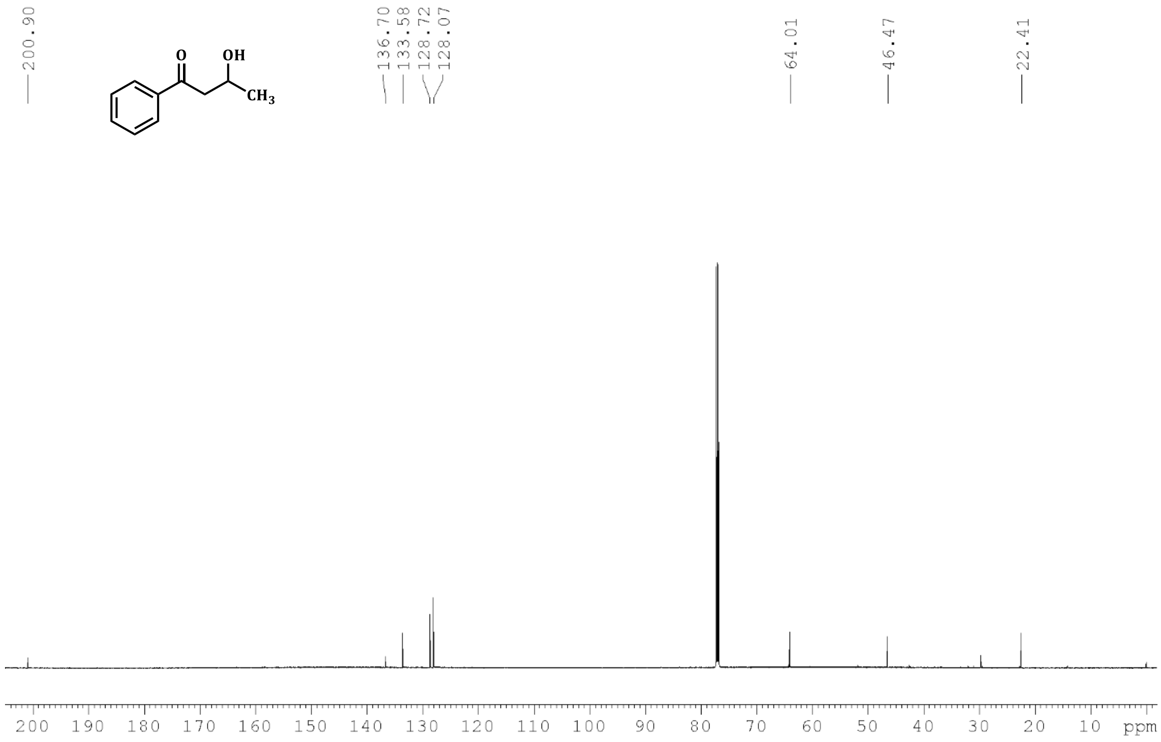

^{1}H NMR (600 MHz, $CDCl_3$) of 1-(methyl(phenyl)amino)propan-2-ol (**2N**)

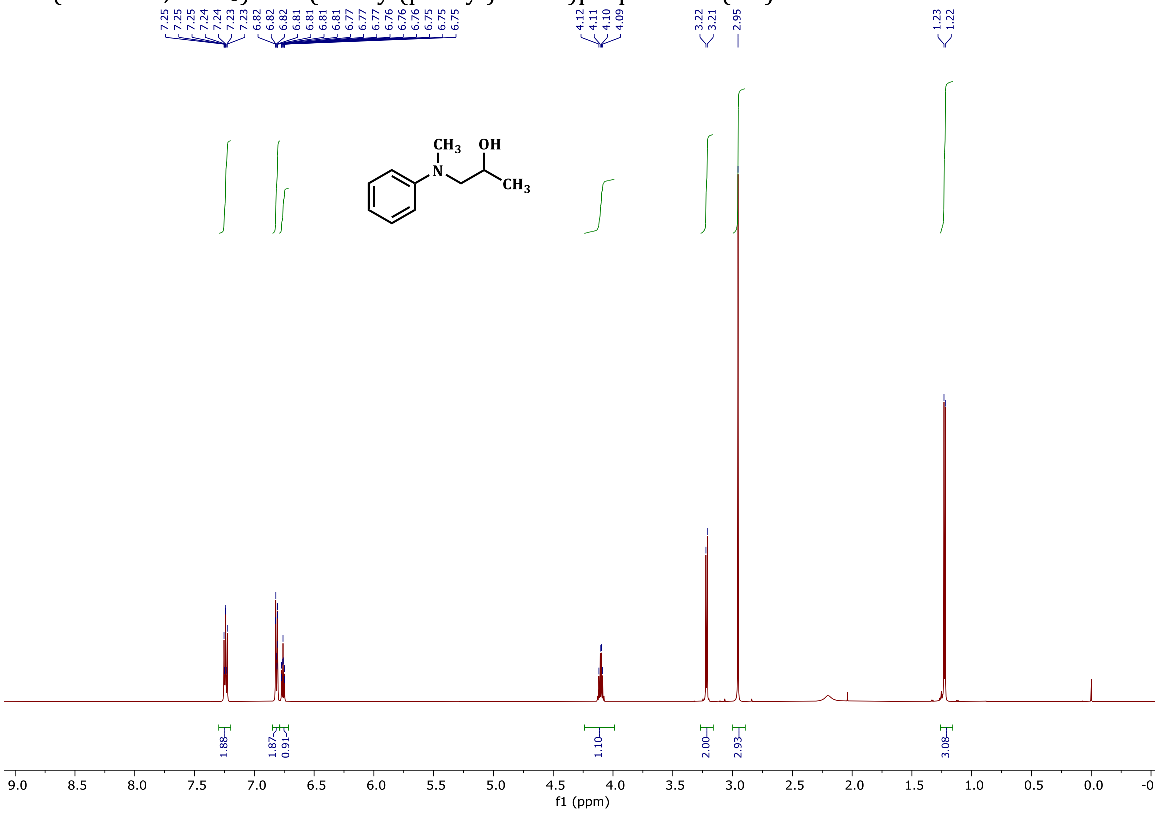

^{13}C NMR (151 MHz, $CDCl_3$) of 1-(methyl(phenyl)amino)propan-2-ol (**2N**)

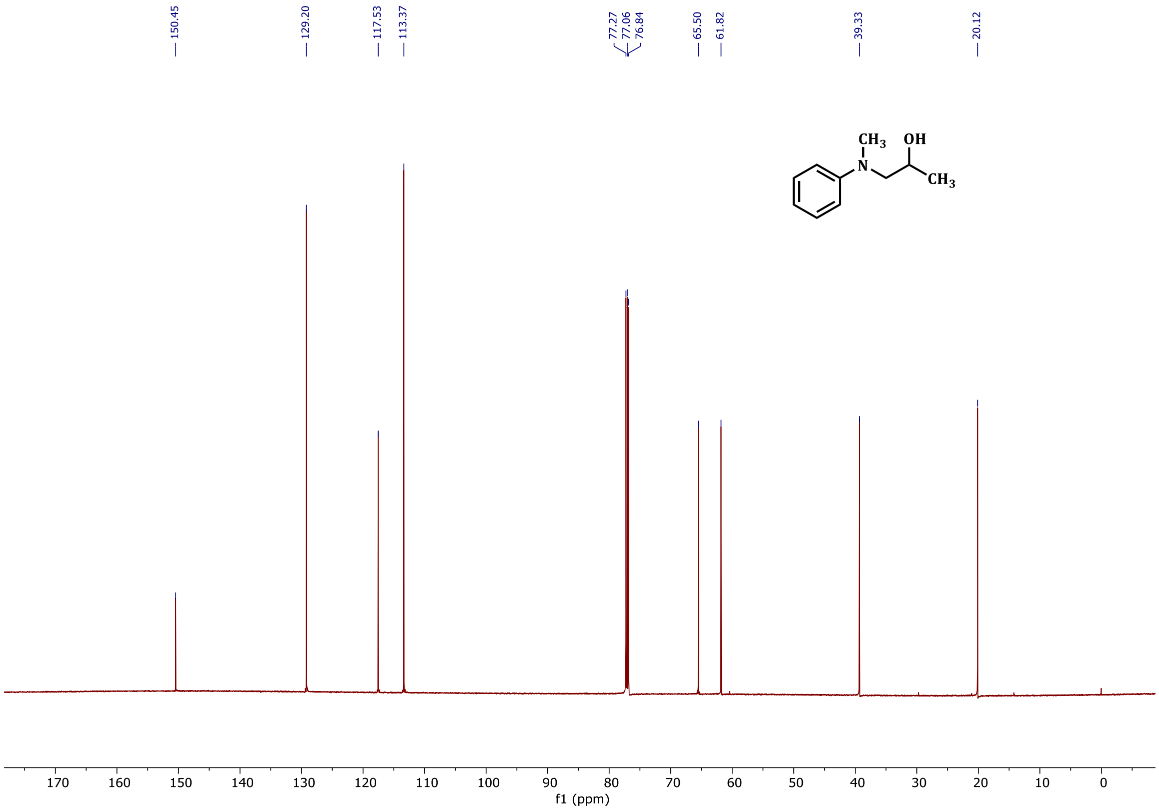

^1H NMR (600 MHz, $CDCl_3$) of 1-(1H-indol-1-yl)propan-2-ol (**2O**)

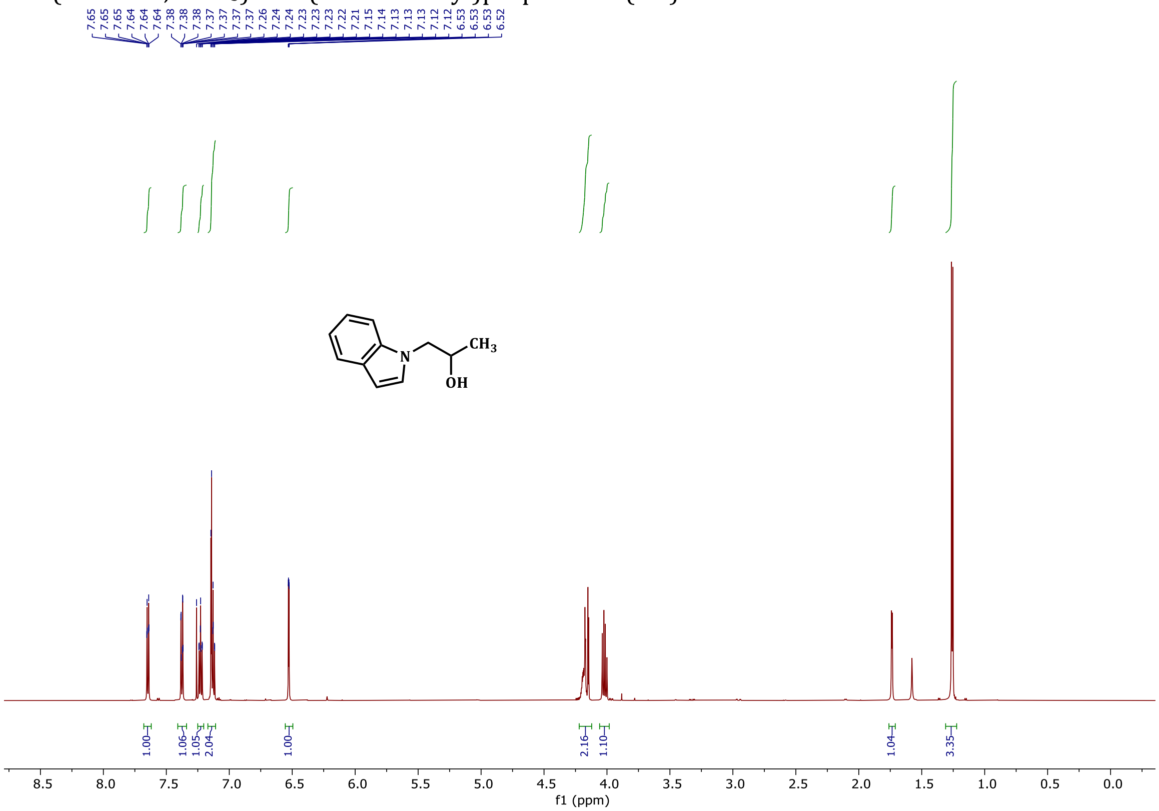

^{13}C NMR (151 MHz, $CDCl_3$) of 1-(1H-indol-1-yl)propan-2-ol (**2O**)

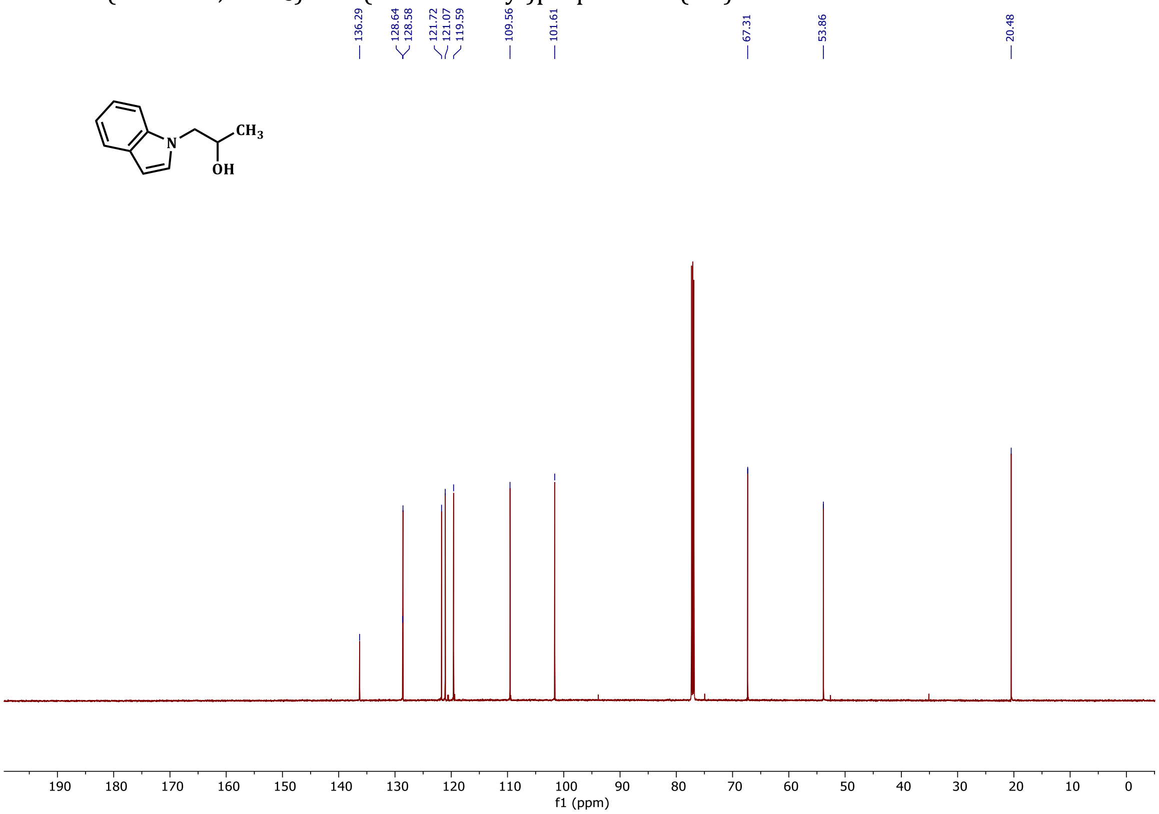

^{1}H NMR (600 MHz, $CDCl_3$) of decan-2-ol (**2P**)

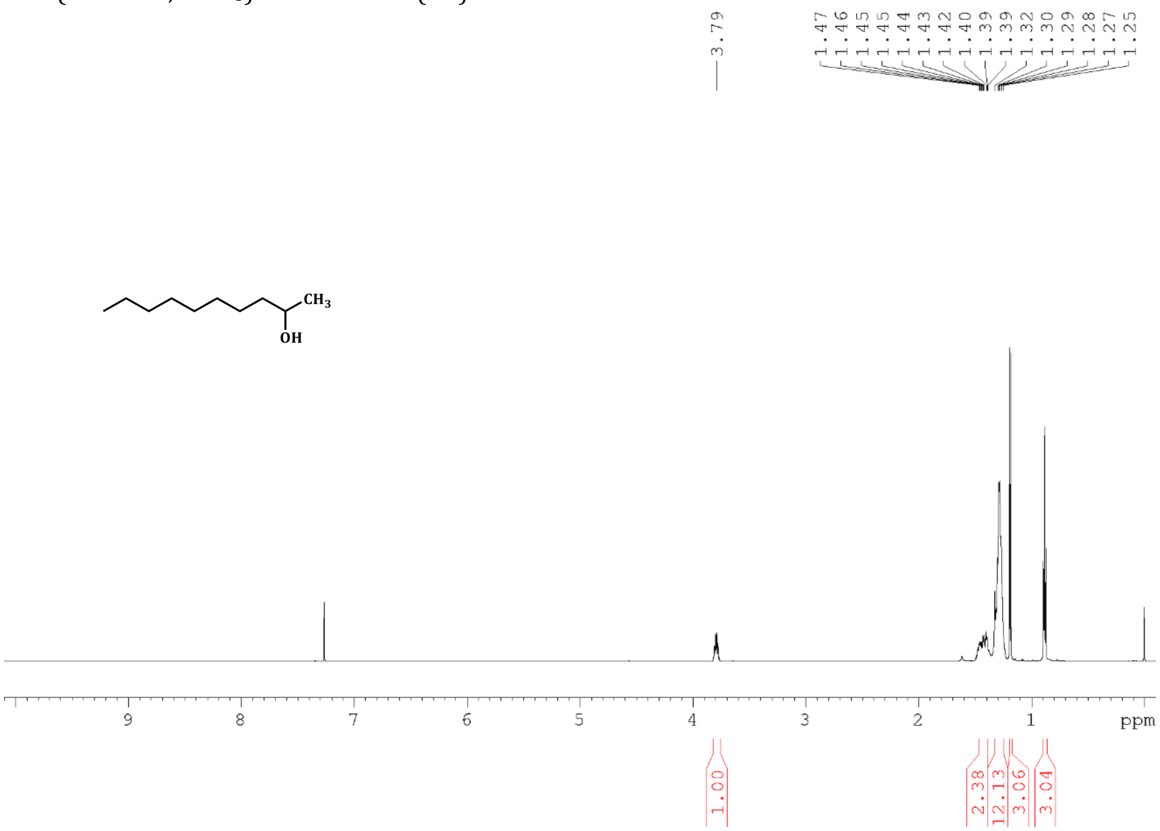

^{13}C NMR (151 MHz, $CDCl_3$) of decan-2-ol (**2P**)

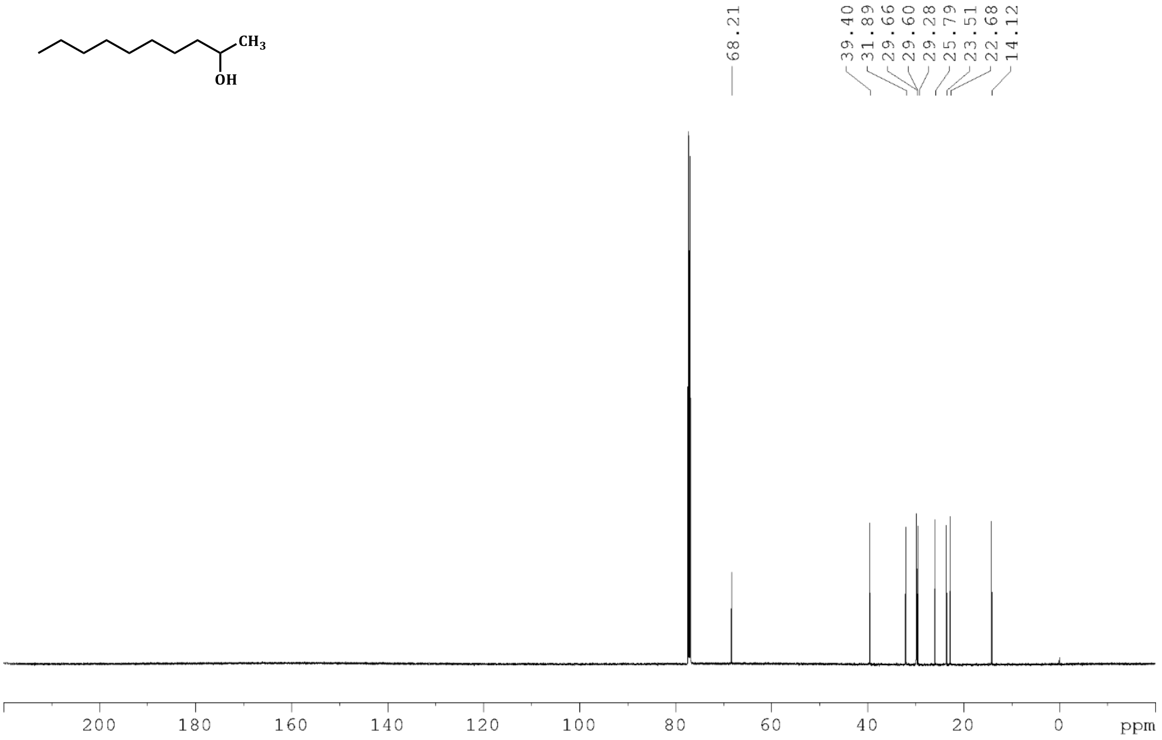

^1H NMR (600 MHz, $CDCl_3$) of 1-cyclohexylpropan-2-ol (**2Q**)

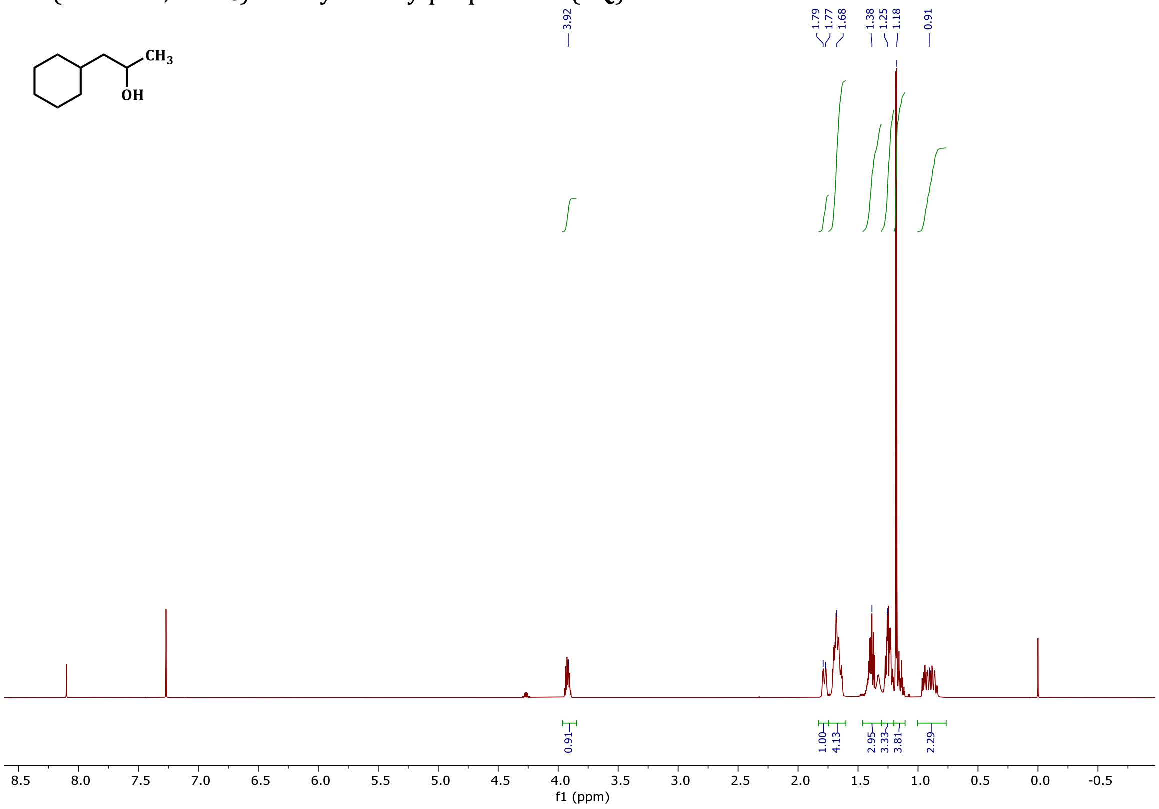

^{13}C NMR (151 MHz, $CDCl_3$) of 1-cyclohexylpropan-2-ol (**2Q**)

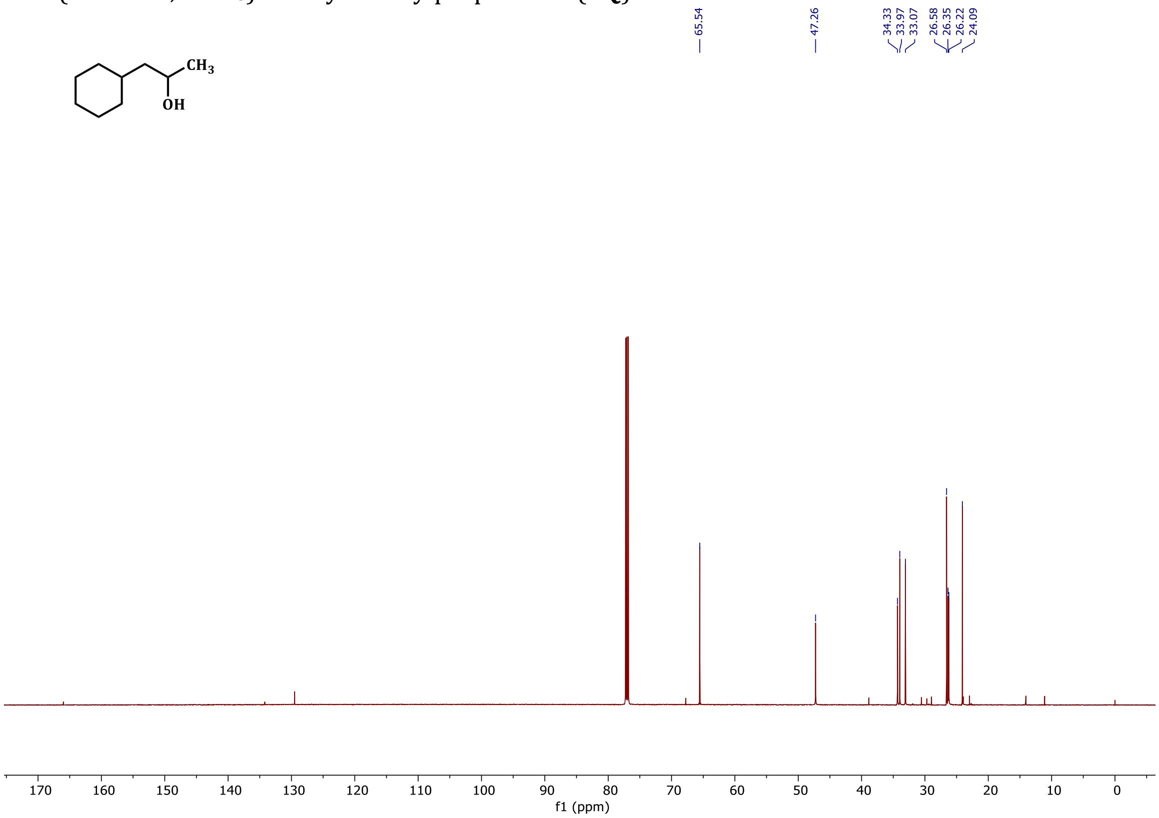

Supplemental References

1. Albéniz, A. C.; Espinet, P.; López-Fernández, R.; Sen, A. (2002). A Warning on the Use of Radical Traps as a Test for Radical Mechanism: They React with Palladium Hydrido Complexes. J. Am. Chem. Soc. 124 (38), 11278-11279. 10.1021/ja0271126.
2. National Institute of Standards and Technology (NIST) Webbook. Mass spectrum: CAS 18729-48-1. https://webbook.nist.gov/cgi/cbook.cgi?ID=C18729481&Units=SI&Mask=200#Mass-Spec.
3. Huang, C.; Ma, W.; Zheng, X.; Xu, M.; Qi, X.; Lu, Q. (2022). Epoxide Electroreduction. J. Am. Chem. Soc. 144 (3), 1389-1395. 10.1021/jacs.1c11791.
4. Jensen, K. L.; Standley, E. A.; Jamison, T. F. (2014). Highly Regioselective Nickel-Catalyzed Cross-Coupling of N-Tosylaziridines and Alkylzinc Reagents. J. Am. Chem. Soc. 136 (31), 11145-11152. 10.1021/ja505823s.
5. Breuillac, A.; Caffy, F.; Vialon, T.; Nicolay, R. (2020). Functionalization of polyisoprene and polystyrene via reactive processing using azidoformate grafting agents, and its application to the synthesis of dioxaborolane-based polyisoprene vitrimers. Polym. Chem. 11, 6479–6491. 10.1039/D0PY00164C.
6. Ai, C.; Zhu, F.; Wang, Y.; Yan, Z.; Lin, S. (2019). SO_2F_2-mediated epoxidation of olefins with hydrogen peroxide. J. Org. Chem. 84 (18), 11928-11934. 10.1021/acs.joc.9b01784.
7. Louka, A.; Stratakis, M. (2021). Deoxygenation of Epoxides with Hexamethyldigermane Catalyzed by Au Nanoparticles on TiO_2. Asian J. Org. Chem. 12, 3364-3369. 10.1002/ajoc.202100581.
8. Aida, K.; Hirao, M.; Funabashi, A.; Sugimura, N.; Ota, E.; Yamaguchi, J. (2022). Catalytic reductive ring opening of epoxides enabled by zirconocene and photoredox catalysis. Chem. 8, 1762-1774. 10.1016/j.chempr.2022.04.010.
9. Garbaccio, R. M.; Brnardic, E. J.; Fraley, M. E.; Hartman, G. D.; Hutson, P. H.; O'Brien, J. A.; Magliaro, B. C.; Uslaner, J. M.; Huszar, S. L.; Fillgrove, K. L.; Small, J. H.; Tang, C. Kuo, Y.; Jacobsen, M. A. (2010). Discovery of oxazolobenzimidazoles as positive allosteric modulators for the mGluR2 receptor. ACS Med. Chem. Lett. 1, 406–410. 10.1021/ml100115a.
10. Shen, C.; Guo, X.; Yu, J.; Zeng, X.-G.; Peng, L.; Zhao, C.-M.; Zhang, F.-L. (2017). Synthesis of 2-(phenoxymethyl)oxirane derivatives through unexpected rearrangement of oxiran-2-ylmethyl benzenesulfonates. Synth. Comm. 47, 273–278. 10.1080/00397911.2016.1258579.
11. Martinez, J.; de la Cruz-Martinez, F.; Martinez de Sarasa Buchaca, M.; Fernandez-Baeza, J.; Sanchez-Barba, L. F.; North, M.; Castro-Osma, J. A.; Lara-Sanchez, A. (2021). Efficient synthesis of cyclic carbonates from unsaturated acids and carbon dioxide and their application in the synthesis of biobased polyurethanes. ChemPlusChem. 86 (3), 460–468. 10.1002/cplu.202100079.
12. Williams, D. B. G.; Cullen, A. (2009). $Al(OTf)_3$-mediated epoxide ring-opening reactions: toward piperazine-derived physiologically active products. J. Org. Chem. 74 (24), 9509-9512. 10.1021/jo9020437.
13. Kumar, A. B.; Anderson, J. M.; Melendez, A. L.; Manetsch, R. (2012). Synthesis and structure-activity relationship studies of 1,3-disubstituted 2-propanols as BACE-1 inhibitors. Bioorg. Med. Chem. Lett. 22 (14), 4740-4744. 10.1016/j.bmcl.2012.05.072.
14. Moriyama, K.; Takemura, M.; Togo. H. (2014). Selective oxidation of alcohols with alkali metal bromides as bromide catalysts: experimental study of the reaction mechanism. J. Org. Chem. 79 (13), 6094–6104. 10.1021/jo5008064.
15. Do, J. Y.; Shin, S. B.; Jeong, S. M.; Jung, M.-Y. (2020). Ring-opening polymerization of cyclic 1,3-oxathiolane-2-thione promoted by neighboring sulfide group and ring contraction. Eur. Polym. J. 131, 109689. 10.1016/j.eurpolymj.2020.109689.
16. Kwon, D. W.; Kim, Y. H.; Lee, K. (2002). Highly Regioselective Cleavages and Iodinations of Cyclic Ethers Utilizing SmI_2. J. Org. Chem. 67 (26), 9488-9491. 10.1021/jo020179r.
17. Redwan, I. N.; Grotli, M. (2012). Method for activation and recycling of trityl resins. J. Org. Chem. 77 (16), 7071-7075. 10.1021/jo300598d.
18. Schabel, T.; Belger, C.; Plietker, B. (2013). A mild chemoselective Ru-catalyzed reduction of alkynes, ketones, and nitro compounds. Org. Lett. 15 (11), 2858-2861. 10.1021/ol401185t.
19. Zhao, Y.; Weix, D. J. (2014). Nickel-catalyzed regiodivergent opening of epoxides with aryl halides: Co-catalysis controls regioselectivity. J. Am. Chem. Soc. 136 (1), 48-51. 10.1021/ja410704d.

20. Bhunia, A.; Bergander, K.; Daniliuc, C. G.; Studer, A. (2021). Fe-catalyzed anaerobic Mukaiyama-type hydration of alkenes using nitroarenes. Angew. Chem. Int. Ed. 60 (15), 8313 –8320. 10.1002/anie.202015740.
21. Thiyagarajan, S.; Gunanathan, C. (2019). Ruthenium-catalyzed selective hydrogenation of epoxides to secondary alcohols. Org. Lett. 21 (23), 9774-9778. 10.1021/acs.orglett.9b03995.
22. Shailaja, M.; Manjula, A.; Rao, B. V. (2010). (Bromodimethyl)sulfonium bromide-mediated thiolysis of epoxides: an easy access to β-hydroxy sulfides and benzoxathiepinones in solvent-free conditions. Synth. Comm. 40 (24), 3629-3639. 10.1080/00397910903458595.
23. Cheng, J.-K.; Loh, T.-P. (2015). Copper- and cobalt-catalyzed direct coupling of sp^3 α-carbon of alcohols with alkenes and hydroperoxides. J. Am. Chem. Soc. 137 (1), 42-45. 10.1021/ja510635k.
24. Pariyar, A.; Asl, H. Y.; Choudhury, A. (2016). Tetragonal vs. hexagonal: structure-dependent catalytic activity of Co/Zn bimetallic metal-organic frameworks. Inorg. Chem. 55 (18), 9250–9257. 10.1021/acs.inorgchem.6b01288.
25. Borowiecki, P.; Dranka, M.; Ochal, Z. (2017). Lipase-catalyzed kinetic resolution of N-substituted 1-(β-hydroxypropyl)indoles by enantioselective acetylation. Eur. J. Org. Chem. 36, 5378–5390. 10.1002/ejoc.201700889.
26. Du, X.; Zhang, Y.; Peng, D.; Huang, Z. (2016). Base-metal-catalyzed regiodivergent alkene hydrosilylations. Angew. Chem. Int. Ed. 55 (23), 6671-6675. 10.1002/anie.201601197.
